# Mendelian randomization while jointly modeling *cis* genetics identifies causal relationships between gene expression and lipids

**DOI:** 10.1038/s41467-020-18716-x

**Published:** 2020-10-01

**Authors:** Adriaan van der Graaf, Annique Claringbould, Antoine Rimbert, BIOS Consortium, Bastiaan T. Heijmans, Peter A. C.’t Hoen, Joyce B. J. van Meurs, Rick Jansen, Lude Franke, Harm-Jan Westra, Yang Li, Cisca Wijmenga, Serena Sanna

**Affiliations:** 1 University of Groningen, University Medical Centre Groningen, Department of Genetics, Antonius Deusinglaan 1, 9713 Groningen, AV The Netherlands; 2 Oncode institute, Office Jaarbeurs Innovation Mile (JIM), Jaarbeursplein 6, 3521 Utrecht, AL The Netherlands; 3 University of Groningen, University Medical Centre Groningen, Department of Pediatrics, Section Molecular Genetics, Antonius Deusinglaan 1, 9713 Groningen, AV The Netherlands; 4 Université de Nantes, CNRS, INSERM, l’institut du thorax, F-44000 Nantes, France; 5 Department of Computational Biology for Individualised Infection Medicine, Centre for Individualised Infection Medicine (CiiM) & TWINCORE, joint ventures between the Helmholtz-Centre for Infection Research (HZI) and the Hannover Medical School (MHH), 30625 Hannover, Germany; 6 Department of Internal Medicine and Radboud Center for Infectious Diseases, Radboud University Medical Center, 6525 Nijmegen, HP The Netherlands; 7 Istituto di Ricerca Genetica e Biomedica (IRGB), Consiglio Nazionale delle Ricerche (CNR), Cittadella Universitaria di Monserrato, 09042 Monserrato, Italy; 8 Molecular Epidemiology, Department of Biomedical Data Sciences, Leiden University Medical Center, Einthovenweg 20, 2333 ZC Leiden, The Netherlands; 9 Department of Human Genetics, Leiden University Medical Center, Einthovenweg 20, 2333 ZC Leiden, The Netherlands; 10 Department of Internal Medicine, Erasmus MC, Dr. Molewaterplein 40, 3015 GD Rotterdam, The Netherlands; 11 Department of Psychiatry, VU University Medical Center, Neuroscience Campus Amsterdam, De Boelelaan 1118, 1081 HV Amsterdam, The Netherlands

**Keywords:** Statistical methods, Gene expression, Genomics

## Abstract

Inference of causality between gene expression and complex traits using Mendelian randomization (MR) is confounded by pleiotropy and linkage disequilibrium (LD) of gene-expression quantitative trait loci (eQTL). Here, we propose an MR method, MR-link, that accounts for unobserved pleiotropy and LD by leveraging information from individual-level data, even when only one eQTL variant is present. In simulations, MR-link shows false-positive rates close to expectation (median 0.05) and high power (up to 0.89), outperforming all other tested MR methods and coloc. Application of MR-link to low-density lipoprotein cholesterol (LDL-C) measurements in 12,449 individuals with expression and protein QTL summary statistics from blood and liver identifies 25 genes causally linked to LDL-C. These include the known *SORT1* and ApoE genes as well as *PVRL2*, located in the *APOE* locus, for which a causal role in liver was not known. Our results showcase the strength of MR-link for transcriptome-wide causal inferences.

## Abstract (web summary)

Mendelian randomization is a useful tool to infer causal relationships between traits, but can be confounded by the presence of pleiotropy. Here, the authors have developed MR-link, a Mendelian randomization method which accounts for unobserved pleiotropy and linkage disequilibrium between instrumental variables.

## Introduction

Mendelian randomization (MR) is a method that can infer causal relationships between two heritable complex traits from observational studies^1,2^. In recent years, MR has gained popularity in the epidemiological field and its application has provided valuable insights into the risk factors that cause diseases and complex traits^1–3^. MR studies have, for example, successfully identified causal relationships between low-density lipoprotein cholesterol (LDL-C) and coronary artery disease, in turn informing therapeutic strategies^4,5^. MR studies have also shown that a causal relationship between high-density lipoprotein cholesterol (HDL-C) and coronary artery disease is unlikely, which is in contrast to previous epidemiological associations^6^. The same approach has been applied to identify molecular marks that are causal to disease^7–10^. Since gene expression is one of these marks, investigating its causal role in complex traits is of particular interest given that complex trait loci are enriched for expression quantitative trait loci (eQTLs)^11^.

MR infers a causal relationship between an exposure (e.g., a risk factor) and an outcome (e.g., a complex trait) by leveraging QTL variants of the exposure as instrumental variables (IVs). The mathematical model behind MR relies on three main assumptions to correctly infer causality: the IVs have to be (i) associated with the exposure, (ii) independent of any confounder of the exposure-outcome association, and (iii) conditionally independent of the outcome given the exposure and confounders. One major challenge of applying MR to gene expression is correcting for deviations from the third assumption, which can occur in the presence of linkage disequilibrium (LD) between the eQTL variants used as IVs, or in the presence of pleiotropy, i.e., when IVs affect the outcome through pathways other than the exposure of interest. Accounting for LD is necessary when gene expression is the exposure trait in MR because, in contrast to the majority of complex traits, the genetic architecture of gene expression is characterized by the presence of strong-acting eQTLs located proximal to their transcript (in *cis*), which are often correlated through LD^12,13^. On top of this, the presence of pleiotropy cannot be excluded a priori given that the majority of variants in our genome are likely to affect one or multiple phenotypes^14–16^. There are MR methods^7,17–21^ that extend standard MR analysis to correct for LD and pleiotropy, however, the application of these methods is not optimal because they require either the removal of pleiotropic IVs from the statistical model^7,19,20^, that all sources of pleiotropy are measured and incorporated into the model^22,23^, or that both the exposure and the outcome are measured in the same cohort^21^. These constraints limit robust inference of gene-expression traits as there are often only a limited number of IVs (i.e., eQTL variants) available, and subsequent removal of outliers will substantially reduce power. Likewise, it is not always possible to measure all sources of pleiotropy because it could come from expression of a gene in a different tissue or even from other unobserved molecular marks or phenotypes.

**Fig. 1:**
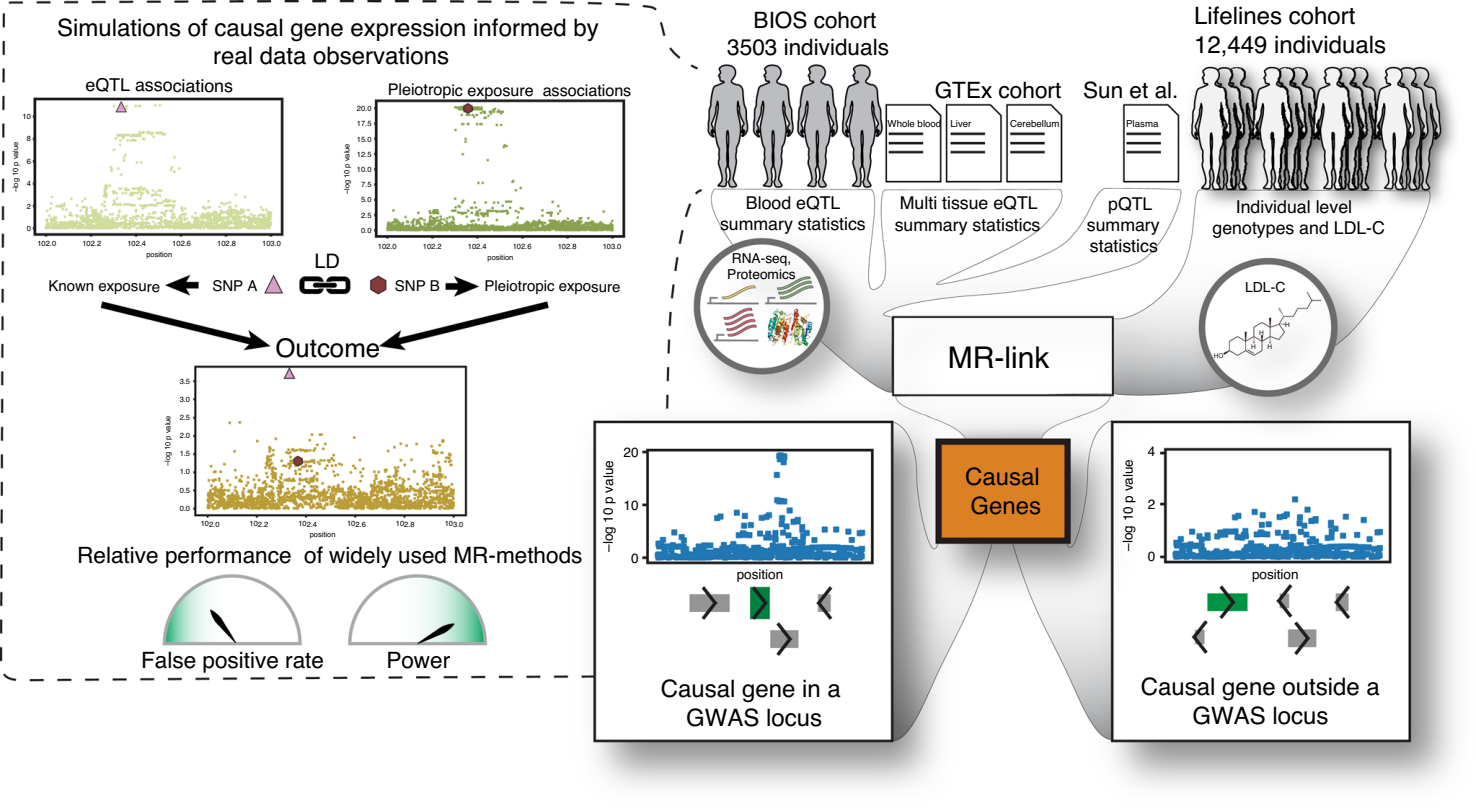
Graphical representation of the study. The Biobank Integrative Omics Study (BIOS) cohort was used to identify expression quantitative trait loci (eQTLs) and characterize the genetic architecture of gene expression. Dashed outbox: Knowledge used in a simulation scheme that mimicked gene-expression traits, including linkage disequilibrium (LD) between eQTL single nucleotide polymorphism (SNPs). We used this simulation to assess the false positive rates and power for widely used Mendelian randomization (MR) methods. We applied our MR method, MR-link, to both the simulations and to individual-level data of low-density lipoprotein cholesterol (LDL-C) in 12,449 individuals (Lifelines) combined with BIOS and GTEx eQTL as well as protein quantitative trait loci (pQTL) summary statistics to identify gene-expression changes and protein level changes that are causally linked to LDL-C within or outside a genome-wide association study (GWAS) locus.

Here we introduce MR-link, an MR method that allows for causal inference in the presence of LD and an unobserved pleiotropic effect, without requiring the removal of pleiotropic IVs or measuring all sources of pleiotropy. MR-link uses summary statistics of an exposure combined with individual-level data on the outcome to estimate the causal effect of an exposure from IVs (i.e., eQTLs if the exposure is gene expression), while at the same time correcting for pleiotropic effects using genetic variants that are in LD with these IVs (*cis-*genetics) (Fig. 1).

We assess the performance of MR-link using simulated data in 100 different scenarios that mimic the genetic architecture of gene expression. We derive this information from eQTL association patterns in a large cohort of samples with genetic and transcriptomics data^13^. Subsequently, we apply MR-link to individual-level data for LDL-C measurements in 12,449 individuals with four different eQTL summary statistic datasets: blood eQTLs identified in the BIOS cohort (Fig. 1) and eQTLs from blood, liver, and cerebellum from the GTEx Consortium^24^ (Fig. 1). We further explore the performance of MR-link on another molecular layer, protein levels, through the application of MR-link on protein quantitative trait loci (pQTL) summary statistics from Sun et al. combined with our LDL-C measurements^25^. Our results in simulated and real data show that MR-link can robustly identify causal relationships between molecular traits—such as gene expression and protein levels—and an outcome (e.g., a complex trait), even when the information for causal inference is very limited.

## Results

### eQTL variants between different genes are often in LD

**Fig. 2:**
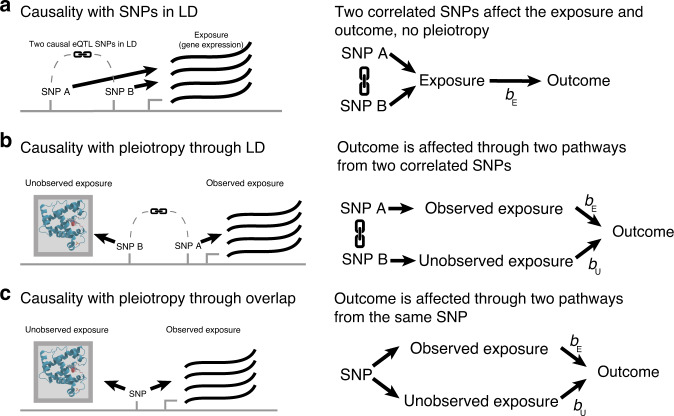
Typical scenarios of pleiotropy in causal inference of gene expression changes as an exposure. Typical scenarios to consider when performing causal inference in gene expression: **a** expression quantitative trait locus (eQTL) single nucleotide polymorphisms (SNPs) used as instrumental variables (IVs) for the same gene (exposure) are in linkage disequilibrium (LD) and pleiotropic effects are absent, **b** pleiotropy is present through LD between IVs for different exposures (pleiotropy through LD), and **c** pleiotropy is present through overlap of the IVs (pleiotropy through overlap). In each panel, the left image shows the genomic context while the right image is a schematic diagram of the corresponding causal effects. Please note that the unobserved exposure trait does not necessarily need to be a protein product: it could be any measured or unmeasured phenotype that is regulated by the genetic locus.

In a standard MR analysis, IVs need to be independent (not in LD) and have to affect the outcome only through the exposure (absence of pleiotropy). Even in absence of pleiotropy, correlated IVs in the *cis* locus may negatively influence an MR analysis (Fig. 2a). In the presence of pleiotropy, we distinguish two scenarios: (i) pleiotropic variants that are in LD with an IV (pleiotropy through LD, Fig. 2b) and (ii) when the IV and the pleiotropic variant are the same and affect the outcome through two distinct mechanisms (pleiotropy through overlap Fig. 2c). If pleiotropy through LD is prevalent, genetic variants in the *cis*-region other than those selected as IVs can be used to explain the pleiotropic effects. Incorporating these variants in an MR model can then account for this pleiotropy through LD (Fig. 2b).

We investigated how often pleiotropy through LD occurs in gene expression by looking at how frequently eQTL variants are shared between genes in *cis*. Using data from the BIOS Consortium, a cohort of 3503 Dutch individuals whose genome and whole-blood transcriptome has been characterized (Fig. 1), we searched for eQTLs located 1.5 megabases (Mb) on both sides of the translated region of 19,960 genes (see “Methods”)^13^. We then applied a summary statistics-based stepwise linear regression approach (GCTA-COJO) to identify jointly significant variants, e.g., one or more variants that jointly associate significantly with expression changes of a gene^26^ (“Methods”). We observed that 54% of the genes with an eQTL at *p* < 5 × 10^−8^ (13,778 genes) had two or more jointly significant eQTL variants at *p* < 5 × 10^−8^ (“Methods”) (Fig. 1 and Fig. 2a). These genetic effects were mostly non-overlapping: only 13.4% of the genes have overlapping (*r*^2^ > 0.99) top eQTL variants. In contrast, genetic variants regulating gene expression of a gene were very often in LD with other eQTLs: 40.6% of top variants are in LD (*r*^2^ > 0.5) between genes, and this percentage increased to 60.3% if all jointly significant eQTL variants were considered (“Methods”).

To strengthen our inferences on the genetic regulation of gene expression in *cis*, we performed statistical fine-mapping using FINEMAP v1.3.1^27^ on 13,276 genes (“Methods”). Only 373 (2.8%) genes have full eQTL overlap (all variants in the top configuration of a gene are identical or in high LD (*r*^2^ > 0.99)), while 33.2% of the genes have at least one variant in *r*^2^ > 0.5 LD with a variant in the top configuration of another gene. These percentages are higher for configurations with larger posterior inclusion probabilities (“Methods”) (Supplementary Data 1), but overall the results are similar to our observations from the GCTA-COJO analysis, i.e., the genetics of gene expression in whole blood is mostly regulated by variants that do not overlap but are in moderate LD with variants associated with gene expression changes of another gene. Based on these results, it seems likely that pleiotropy through LD is more common than pleiotropy through overlap in gene-expression traits.

### MR-link outperforms other methods in discriminative ability

We have developed an MR method, MR-link, that uses the genetic region surrounding IVs as a covariate to correct for pleiotropic effects (“Methods”, Fig. 2 and Supplementary Note 1). The model underlying MR-link is informed by the observation that the genetic regulation of gene expression is characterized mostly by eQTLs that are in LD, but not overlapping, between genes. This suggests that the variants in the genetic vicinity of the IVs can be used to correct for pleiotropic effects.

MR-link gathers information from all genetic variants in LD with an IV to jointly model the outcome through the IVs and their genetic vicinity (“Methods”). Compared to other MR methods that require summary statistics of both the exposure and the outcome (two-sample MR), our approach adds a requirement of individual-level data for the outcome, but has the advantage that it can perform causal inference even when only a single IV is available. Strictly speaking, MR-link corrects for pleiotropy under the assumption that pleiotropy can be better explained by variants in LD with the IV (pleiotropy through LD) (Fig. 2b) and that pleiotropy through overlap is absent (Fig. 2c). In the case of a single IV, this assumption needs to be fully accounted for, but when multiple IVs are available, this assumption can be relaxed somewhat. Differences in effect sizes between IVs can be used to distinguish the causal effect of interest from a pleiotropic effect in the same way that multivariable MR corrects for pleiotropy^22^. Of note, MR-link does not require the source of pleiotropy to be specified in the model; MR-link can account for pleiotropic effects arising from, for instance, gene expression in other tissues or from other molecular layers or phenotypes.

We assessed the performance of MR-link under different scenarios and compared it to four other MR methods: Inverse variance weighting (IVW), which assumes the absence of LD and pleiotropy, and the pleiotropy-robust methods MR-Egger, LDA-MR-Egger, and MR-PRESSO (Table 1)^17–19,28^. In addition, we compared MR-link to the widely used Bayesian colocalization method *coloc*^29^, although this is not a formal test for assessing causal relationships, but rather a way to evaluate if two traits share the same causal variant(s) in a locus^29^.

**Table 1:** MR methods assessed in this study.

MR method	Data required	Pleiotropy correction	LD correction	Minimum no. of IVs required
MR-link	Sumstats: E; ILD: O	Yes	Yes	1
Inverse variance weighted (IVW)^28^	Sumstats: O, E	No	No	1
LDA-MR-Egger^17^	Sumstats: O, E, LDR	Yes	Yes	3
MR-Egger^18^	Sumstats: O, E	Yes	No	3
MR-PRESSO^a,^^19^	Sumstats: O, E	Yes	No	3^a^

MR methods assessed in our simulation with information about the type of data needed to make a causal estimate, the ability to correct for pleiotropy or linkage disequilibrium, and the minimum number of instrumental variables required.

*IVW* inverse variance weighting, *Sumstats* summary statistics, *ILD* individual-level data, *O* outcome, *E* exposure, *LDR* linkage disequilibrium reference panel. For more details, see “Methods”.

^a^We refer here to the MR-PRESSO test that reports estimates after identifying and removing outliers, as the test without outliers generalizes to IVW estimates.

We simulated causal relationships between an exposure and an outcome in a 5 Mb region, based on LD structure estimated for 403 European samples from the 1000 Genomes project^30^ (“Methods”). All tested MR methods were assessed in 1500 simulated datasets for 100 different scenarios that varied with respect to the absence or presence of causality, the absence or presence of pleiotropy, and the number of causal eQTL variants. We initially evaluated two approaches to select QTL variants as IVs: GCTA-COJO (v1.26.0) and *p* value clumping (“Methods”)^26,31^. We observed that GCTA-COJO was best suited for IV selection because: (i) the median number of IVs identified by GCTA-COJO better represented the number of simulated causal variants (Supplementary Data 2) and (ii) the false-positive rates (FPRs) in the MR analysis using the IVW method were lower (median FPR was 0.057 using GCTA-COJO versus 0.115 using clumping) (Supplementary Fig. 1 and Supplementary Data 2). We therefore selected IVs for the exposure using the GTCA-COJO approach in subsequent analyses.

**Fig. 3:**
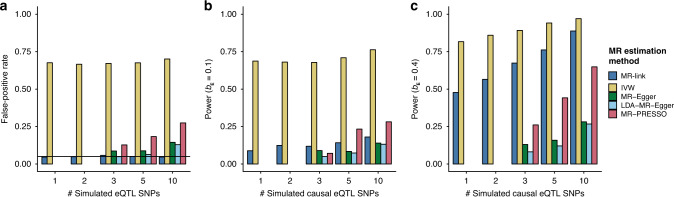
Relative performance of different MR methods. The figure shows performance of MR methods on simulations representing the pleiotropy through linkage disequilibrium (LD) scenario (depicted in Fig. 2b) when 1, 3, 5, or 10 causal expression quantitative trait locus (eQTL) single nucleotide polymorphisms (SNPs) were simulated (“Methods”). **a** False positive rates (at alpha = 0.05) in scenarios where no causal relationship is simulated. **b** Power to detect a small causal effect (at alpha = 0.05). **c** Power to detect a large causal effect (at alpha = 0.05). Note that MR-link is the only MR method that can adjust for pleiotropy when only one or two instrumental variables are available. MR methods that had fewer than 100 out of 1500 estimates in a scenario are not shown (“Methods”). Extended results, including those that are not shown in the figure, can be found in Supplementary Data 3.

When we simulated pleiotropy through LD with no causal effect of the known exposure on the outcome (Figs. 2b, 3a, Supplementary Data 3 and “Methods”), all existing MR-methods showed inflated FPRs (up to 0.71, 0.15, 0.13, and 0.27 for IVW, MR-Egger, LDA-MR-Egger, and MR-PRESSO, respectively), whereas MR-link presented an FPR close to expectation (median: 0.05, maximum: 0.058). In addition, for LDA-MR-Egger, MR-Egger, and MR-PRESSO, the FPR was undesirably dependent on the number of causal SNPs simulated (Fig. 3a).

In the scenarios of pleiotropy through LD and non-null causal effects (*b*_E_ = 0.05, *b*_E_ = 0.1, *b*_E_ = 0.2, and *b*_E_ = 0.4), MR-link has high detection power (up to 0.89) and strongly outperforms all other pleiotropy-robust methods (maximum detected power was 0.28 for MR-Egger, 0.26 for LDA-MR-Egger and 0.65 for MR-PRESSO) (Fig. 3b, c, Supplementary Data 3 and “Methods”). Among all the methods tested, including MR-link, and for all scenarios, IVW had the greatest detection power but also an inflated FPR (minimum FPR: 0.63), making this MR method unreliable in such pleiotropic scenarios (“Methods”).

When we simulated increasing levels of pleiotropy through overlap (Fig. 2c and “Methods”), a situation we expect to be rare in real-world scenarios based on our observation in the BIOS cohort, we observed that all methods including MR-link have increased FPRs (up to 0.22 for MR-link, 0.77 for IVW, 0.10 for LDA-MR-Egger, 0.13 for MR-Egger, and 0.30 for MR-PRESSO) (Supplementary Data 4). Nonetheless, MR-link remains a powerful method when a causal effect is simulated: maximum power was 0.79 for MR-link, 0.98 for IVW, 0.29 for MR-Egger, 0.28 for LDA-MR-Egger, and 0.65 for MR-PRESSO (Supplementary Data 4). Although IVW again had the highest power (0.98) here, the FPR was likewise highly inflated (0.77).

Finally, we compared MR-link to the *coloc* package using the area under the receiver operator characteristic curve (AUC) metric as well as FPRs and power (calculated using *coloc* PP4 > 0.9 as a threshold) (“Methods”). We used the AUC metric because *coloc* provides posterior probabilities of causal variant sharing and not *p* values (“Methods”). As *coloc* assumes that the exposure and the outcome share only one causal variant, we also included the recently implemented *coloc* variations (*coloc-cond* and *coloc-masked*) in our comparison. These variations are expected to perform better in scenarios with multiple causal variants^32^. When comparing MR-link to the *coloc* variations through the AUC metric, we find that MR-link consistently outperforms *coloc* and *coloc-masked* in all scenarios, and *coloc-cond* in pleiotropic scenarios. In non-pleiotropic scenarios, MR-link and *coloc-cond* have approximately the same performance (Supplementary Fig. 2 and Supplementary Data 5). As expected, *coloc-cond* has better discriminative performance compared to the original *coloc* when multiple causal variants are simulated (Supplementary Fig. 2 and Supplementary Data 5).

To illustrate detection rates in standard coloc settings as they may be used in a real-world analysis, we determined power and FPR for all coloc variations at a PP4 threshold of > 0.9 (Supplementary Fig. 3 and Supplementary Data 6). In the non-pleiotropic case, *coloc* and *coloc-cond* have the best detection power (up to 0.79 for *coloc* and 0.76 for *coloc-cond*), combined with near zero FPRs (max: 0 for *coloc* and 0.0006 for *coloc-cond*) while *coloc-masked* has lower power (up to 0.40) with a zero FPR (Supplementary Fig. 3a–c) (Supplementary Data 6). In simulations of pleiotropy through LD, all *coloc* methods have increased FPRs (medians: 0.026 for *coloc*, 0.142 for *coloc-cond*, and 0.0037 for *coloc-masked*) with a decrease in power relative to the non-pleiotropic simulations (max: 0.37 for *coloc*, 0.43 for *coloc-cond*, and 0.14 for *coloc-masked*) (Supplementary Fig. 3d–f and Supplementary Data 6). These patterns were even more apparent in cases of pleiotropy through overlap (Supplementary Fig. 3g–i and Supplementary Data 6). This comparison through FPRs and power indicates again that MR-link has superior discriminative ability over *coloc* variations, especially in the presence of pleiotropy.

### MR-link identifies gene expression causal to LDL-C levels

We applied MR-link to four separate summary statistics-based eQTL datasets combined with individual-level genotype data and LDL-C measurements in 12,449 individuals from the Lifelines cohort^33^ (Fig. 1). We assessed the causal effect of gene expression changes in (i) whole blood (using eQTLs from BIOS (*n* = 3503) and GTEx (*n* = 369)), (ii) liver as the main tissue important for cholesterol metabolism (using eQTLs from GTEx, *n* = 153), and (iii) cerebellum tissue (using eQTLs from GTEx, *n* = 154) as a tissue not involved in cholesterol metabolism but with similar sample size (and thus power) to liver tissue^24,34^.

Transcriptome-wide application of MR-link to these eQTL datasets identified 24 significant genes whose variation in blood (18 using BIOS eQTLs, 2 using GTEx eQTLs) or liver (4 genes) was causally related to LDL-C (Tables 2, 3, Supplementary Tables 1 and  2). No significant genes were found in the cerebellum (Supplementary Table 2).

**Table 2:** MR-link results using BIOS blood eQTLs.

Gene name	Causal effect	*p* Value	#IVs	Biological function and link to LDL-C
*IGLC5*	−0.05313	2.08E−09	1	Immunoglobulin lambda constant 5 (*IGLC5*) is a pseudogene; three other genes in the same locus and belonging to the same family appear in this table (*IGLV-70*, *IGLV4-69*, and *IGLC6*). *IGLC5* does not have a known function in LDL-C metabolism
*KB-1460A1.5*	0.15354	1.35E−08	1	*KB-1460A1.5* is an RNA gene with unknown function in LDL-C metabolism
*ABO*	−0.08224	4.84E−08	4	Alpha 1-3-N-Acetylgalactosaminyltransferase And Alpha 1-3-Galactosyltransferase (*ABO*) is a protein-coding blood group gene. *ABO* is located in LDL-C GWAS locus^35,36,72^. *ABO* is part of the KEGG pathway *Glycosphingolipid biosynthesis* although the direct link between *ABO* and LDL-C remains unclear.
*UNC5B*	−0.01235	4.87E−08	1	Unc-5 Netrin Receptor B (*UNC5B*) is a protein-coding gene and a receptor for the *NETRIN1* protein. It is associated to the disease Hyperinsulinemic Hypoglycemia familial 3. *UNC5B* is localized in lipid rafts, membrane compartments that contain high levels of cholesterol and lipids^73^. The direct link of this gene to LDL-C remains unclear.
*TMEM176B*	−0.0287	1.11E−07	4	Transmembrane protein 176B (*TMEM176B*) is a protein-coding gene located in the *TMEM176B*-*TMEM176A*-*AOC1* GWAS locus for HDL-C^35^. Two IVs for *TMEM176B* are overlapping with IVs for *TMEM176A*.
*REEP1*	−0.02183	1.25E−07	1	Receptor Accessory Protein 1 (*REEP1*) is a protein-coding gene. Mutations of the N-terminal of REEP1 lead to accumulation in lipid droplets in the endoplasmic reticulum^74^. *REEP1* does not have a known function in LDL-C metabolism.
*KRT79*	−0.05904	1.54E−07	2	Keratin 79 (*KRT79*) is a protein-coding gene, that promotes sebaceous gland maintenance in mice hair follicles^75^. The sebaceous gland produces up to 90% of the lipids present in the epidermis. Although a direct link to LDL-C levels remains unclear.
*IGLC6*	−0.07662	2.21E−07	3	Immunoglobulin lambda constant 6 (*IGLC6*) is a pseudogene; three other genes in the same locus belonging to the same family appear in this table (*IGLV-70*, *IGLV4-69*, and *IGLC5*). *IGLC6* does not have a known function in LDL-C metabolism.
*MAP1LC3A*	0.037236	2.32E−07	1	Microtubule Associated Protein 1 Light Chain 3 Alpha (*MAP1LC3A*) is a protein-coding gene. *MAP1LC3A* has no known function in cholesterol metabolism.
*AOC1*	−0.00861	2.48E−07	1	Amine Oxidase, Copper Containing 1 (*AOC1*) is a protein-coding gene located in the *TMEM176A-TMEM176B-AOC1* GWAS locus for HDL-C^35^.
*IGLV4-69*	0.08767	3.1E−07	1	Immunoglobulin Lambda Variable 4-69 (*IGLV4-69*) is a protein-coding gene; three other genes in the same locus and belonging to the same family appear in this table (*IGLV-70*, *IGLC5*, and *IGLC6*). *IGLV4-69* does not have a known function in cholesterol metabolism
*SYCP2L*	−0.01941	4.09E−07	2	Synaptonemal Complex Protein 2 Like (*SYCP2L*) is a protein-coding gene, located in a GWAS locus for antiphospholipid syndrome and fatty acid measurements. *SYCP2L* does not have a known function in LDL-C metabolism^38,76^.
*C10orf10*/*DEPP1*	−0.06893	4.74E−07	1	*DEPP1* (also known as *C10orf10*) is an autophagy regulator highly expressed in adipose tissue. *DEPP1* overexpression in mice reduces glucose and triglyceride levels^77^, although a direct link of this gene to LDL-C metabolism remains unclear.
*TMEM176A*	−0.02242	4.77E−07	3	Transmembrane protein 176A (*TMEM176A*) is a protein-coding gene located in the *TMEM176B*-*TMEM176A*-*AOC1* GWAS locus for HDL-C^35^. Two IVs for *TMEM176A* are overlapping with *TMEM176B*.
*RP11-18H21.1*	0.029575	5.02E−07	2	*RP11-18H21.1* is a non-coding RNA gene without a known function in LDL-C metabolism.
*TACSTD2*	−0.01865	8.65E−07	2	Tumor Associated Calcium Signal Transducer 2 (*TACSTD2*) is a protein-coding gene located on the cell membrane involved in the superpathway *Ca, cAMP,* and *Lipid Signaling*. The function of *TACSTD2* in LDL-C metabolism is unclear.
*MSLN*	−0.02566	1.39E−06	4	Mesothelin (*MSLN*) is a protein-coding gene, its link to LDL-C is unclear.
*IGLVI-70*	0.112435	3.49E−06	2	Immunoglobulin Lambda Variable (I)-70 (*IGLVI-70*) is a pseudogene; three other genes in the same locus and belonging to the same family appear in this table (*IGLV4-69*, *IGLC5*, and *IGLC6*). *IGLVI-70* does not have a known function in cholesterol metabolism

This table shows 18 Bonferroni-significant genes identified by MR-link as causal for LDL-C levels in the analysis that included eQTLs from the BIOS cohort. Gene names are according to ENSEMBL GENES 96 database (human Genome build 37). The causal effect estimate represents the changes in LDL-C (mg per dL) per standard deviation increase in gene expression. *p* values listed in this table are not adjusted for multiple testing (MR-link calibrated two-sided *p* value, see Supplementary Note 1). Full summary statistics of the genes are shown in Supplementary Table 1.

*LDL-C* low-density lipoprotein cholesterol, *IV* instrumental variable, *HDL-C* high-density lipoprotein cholesterol, *GWAS* genome-wide association study, *siRNA* small interfering RNA.

**Table 3:** MR-link results using GTEx liver eQTLs.

Gene name	Causal effect	*p* Value	#IVs	Biological function and link to LDL-C
*PVRL2*	0.3177	3.0E−14	1	Poliovirus receptor-related 2 (*PVRL2*) is a protein-coding gene also known as *NECTIN2*. It is a cell-membrane protein located in the LDL-C GWAS locus *APOE*. siRNA experiments show that LDL-C uptake is increased in cells upon its downregulation^48^. *PVRL2* knockout mice also had less atherosclerosis^46^. Both studies indicate a reduction in LDL-C upon downregulation of *PVRL2*.
*PSRC1*	−0.0847	3.99E−09	1	Proline and serine rich coiled-coil 1 (*PSRC1*) is a protein-coding gene located in an LDL-C GWAS locus^35^. *PSRC1* has not been found to have an effect on cholesterol despite being targeted in a specific functional study^43^. The IV for *PSRC1* is overlapping with the IV for *CELSR2* and *SORT1*.
*SORT1*	−0.0865	5.89E−09	1	Sortilin (*SORT1*) is a protein-coding gene located in an LDL-C GWAS locus^35^. siRNA and knockdown experiments have functionally validated that *SORT1* has a negative effect on LDL-C levels^41,42^. The IV for *SORT1* is overlapping with the IV for *PSRC1* and *CELSR2*.
*CELSR2*	−0.0993	6.8E−08	1	Cadherin EGF LAG Seven-Pass G-Type Receptor 2 (*CELSR2*) is a protein-coding gene located in an LDL-C GWAS locus^35^. *CELSR2* has not been found to have an effect on cholesterol despite being targeted by a specific functional study^43^. The IV for *CELSR2* is overlapping with the IV for *PSRC1* and *SORT1*.

This table lists four Bonferroni-significant genes that were identified using GTEx liver eQTLs. Gene names are according to ENSEMBL GENES 96 database (human Genome build 37). The causal effect estimate represents changes in LDL-C (mg per dL) per standard deviation increase in gene expression. *p* Values listed in this table are not adjusted for multiple testing (MR-link calibrated two-sided *p* values, see Supplementary Note 1). Full summary statistics of these genes are shown in Supplementary Table 2. *LDL-C* low-density lipoprotein cholesterol, *GWAS* genome-wide association study, *siRNA* small interfering RNA, *IV* instrumental variable.

MR analysis that used whole-blood eQTLs from GTEx was, as expected, underpowered compared to the analysis using BIOS eQTLs. Only two genes were found to be significant here, but they were not significant in the analysis that used BIOS eQTLs, where a more robust estimate could be made thanks to higher number of IVs identified (Supplementary Fig. 4a). Despite the limited power, we observed high concordance between effect sizes from the two analyses for all genes that showed nominal significance (*p* < 0.05) in the analysis that used BIOS eQTLs, with 94.8% of genes showing the same effect direction (Supplementary Fig. 4b).

Several genes located in genome-wide association study (GWAS) loci for cholesterol metabolism were found significant in the MR analysis that used blood eQTLs from BIOS, using a Bonferroni threshold that accounted for 13,778 genes being tested (0.05/13778 = 3.6 × 10^−6^). These include *ABO*, located in a LDL-C locus, *AOC1*, *TMEM176A*, and *TMEM176B*, which are all located in the same HDL-C-associated locus^35,36^, and *SYCP2L*, which is located in a GWAS locus for polyunsaturated fatty acids and related to LDL-C levels^37,38^. For the other genes identified, there was no evidence in the literature for a direct role in cholesterol metabolism, although some interesting patterns were evident. For example, we observed multiple genes involved in immunoglobulin production (*IGLC5*, *IGLC6*, *IGLV4-69*, and *IGLVI-70*) and insulin metabolism (*UNC5B*, *DEPP1*), mechanisms that are consistent with the role of cholesterol in inflammation and insulin resistance^39,40^. For all 18 genes, the effect direction estimated by MR-link was concordant with the direction estimated by other MR-methods when they were available, except in the case of *MSLN*, where only LDA-MR-Egger gave discordant results compared to all other methods (Table 1, Supplementary Fig. 5, and Supplementary Table 3). Interestingly, 17 of the 18 genes did not pass significance after multiple testing correction using the other tested methods: only *ABO* passed Bonferroni significance and only when using the IVW method (Table 1, Supplementary Fig. 5, and Supplementary Table 3). In 13 genes, a causal effect could not be estimated by MR-Egger, LDA-MR-Egger, and MR-PRESSO because there were too few IVs. Furthermore, MR-PRESSO did not make a causal estimate in the remaining 5 genes as it identified too many outliers (Table 1, Supplementary Fig. 5, and Supplementary Table 3).

In the MR analysis using eQTLs from liver, all the genes identified at the Bonferroni significance level of 3.2 × 10^−5^ (0.05/1557) fall within LDL-C GWAS loci. Among these, we found a negative causal effect for the well-known *SORT1* gene (MR-link calibrated two-sided *p* = 5.9 × 10^−9^). Multiple functional studies have shown that this gene encodes the protein Sortilin (encoded by *SORT1*) and that it affects plasma LDL-C levels by acting on clearance of LDL-C and on secretion of very-LDL (VLDL) by the liver^41–43^ (Table 3 and Supplementary Table 2). We also found two other genes in the same GWAS locus, *PSRC1*, and *CELSR2*, but the IV (only one was found) for these genes was identical to that of *SORT1* due to the high correlation between expression levels of these genes. Full overlap of a single IV in this locus makes it is impossible to discern causal from pleiotropic genes using MR-methods, including MR-link. The fourth gene found to be significant using liver eQTLs is *PVRL2* (MR-link calibrated two-sided *p* = 3 × 10^−14^), which is located in the *APOE* locus associated to LDL-C (Table 3)^35,36^. For *PVRL2*, we estimated a positive causal effect; higher expression of *PVRL2* is causally related to higher LDL-C (Table 3). *PVRL2* is 17.5 kb downstream of the *APOE* gene, and two common missense polymorphisms in *APOE* account for a large fraction of the association signal^36,44^. Interestingly, in the most recent GWAS meta-analysis for lipids, 19 jointly significant LDL-C variants were found spanning a 162 kb region that encompasses *PVRL2*^36^. This indicates that, while missense mutations in *APOE* play a major role, other genes in this locus are also likely involved in LDL-C regulation and that pleiotropic effects are to be expected. Our analyses indicate that *PVRL2* is one of the causal genes at this locus. The positive effect of *PVRL2* on LDL-C was also seen in the analysis that used blood eQTLs from BIOS (MR-link calibrated two-sided *p* = 4.3 × 10^−5^), although it did not pass our significance threshold in that analysis. Likewise, variation in gene expression of *PVRL2* in blood has been found to be associated with LDL-C in a transcriptome-wide association analysis carried out in a very large genetic association study^36^. Of note, since the LD between IVs used in the analysis of blood and liver eQTLs was low (*r*^2^ < 0.2), the results potentially indicate a dual causal role for *PVRL2* across these two tissues.

**Fig. 4:**
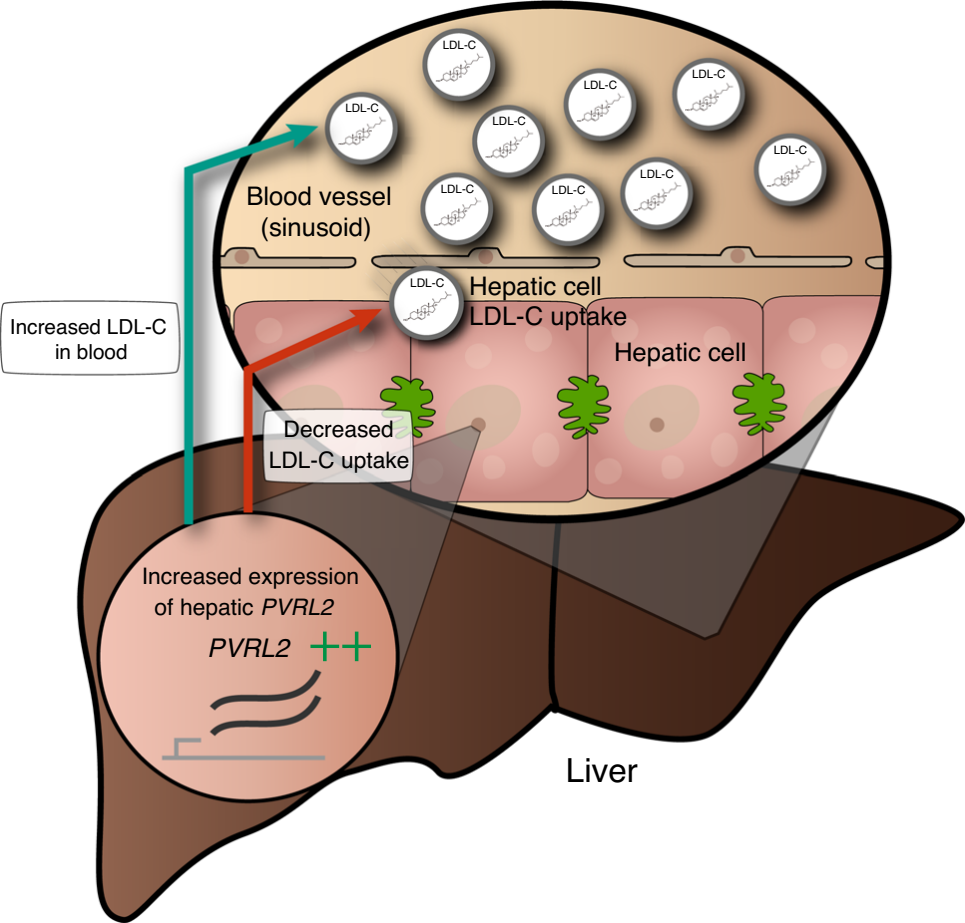
Biological interpretation of *PVRL2*. Functional and statistical evidence for the causal effect of *PVRL2* on low-density lipoprotein cholesterol (LDL-C) levels. The teal arrow indicates a positive causal relationship between *PVRL2* expression in liver and LDL-C levels in plasma—this relationship was detected in our MR analysis. The red arrow indicates a negative causal relationship between *PVRL2* expression and LDL-C uptake in hepatic cells—this relationship was detected in small interfering RNA (si) experiments described in Blattman et al.^48^.

*PVRL2* has mostly been studied in the context of atherosclerosis, where it has been shown to act as cholesterol-responsive gene involved in *trans*-endothelial migration of leukocytes in vascular endothelial cells, a key feature in atherosclerosis development^45–47^. Our results indicate a role for *PVRL2* in modulating plasma levels of LDL-C via its expression variation in the liver. Biologically the role in liver could be explained by increased production of very-LDL or decreased LDL-C uptake (Fig. 4). In line with this hypothesis, a siRNA screen in hepatic cell lines of genes in the *APOE* locus showed that downregulation of *PVRL2* gene expression promotes LDL-C uptake^48^ (Fig. 4). Overall, our results and existing functional evidence support that *PVRL2* expression is correlated with LDL-C levels and show a causal effect in liver (Fig. 4).

### MR-link confirms ApoE changes affect LDL-C levels

To assess the effectiveness of MR-link in proteomics measurements, we combined the aforementioned LDL-C measurements in the Lifelines cohort with *cis*-pQTL summary statistics of 471 plasma protein measurements (measured using the SOMAscan platform in a cohort of 3301 individuals) (“Methods”)^25,49^. One protein passes the Bonferroni multiple testing threshold (*p* < 1.05 × 10^−4^): ApoE3, an isoform of ApoE (causal effect: 0.40 (+/−0.13 s.e.), MR-link calibrated two-sided *p* = 4.65 × 10^−5^, SOMAmer ID: APOE.2937.10.2). pQTLs were also available for ApoE2 (SOMAmer ID: APOE.5312.49.3), another isoform of ApoE but the causal effect was weaker and did not pass the Bonferroni threshold (causal effect = 0.56 (+/−0.24 s.e.), MR-link calibrated two-sided *p* = 0.002)^44^. These results are in line with the well-known causal relationship between increased ApoE plasma levels and LDL-C, and the widely described stronger impact of the E3 isoform compared to the E2 isoform^44^. Interestingly, MR-link did not estimate BGAT, the protein product of *ABO*, to be significant in this dataset (SOMAmer ID: ABO.9253.52.3, MR-link calibrated two-sided *p* = 0.18) We compared the IVs identified for BGAT (rs9411463 and rs72775494) with those used in the *ABO* blood eQTL analysis and found that only one IV for the BGAT protein was in LD (rs9411463) with any of the four IVs for *ABO* expression in BIOS. This scenario is in line with the overall patterns observed in the proteomics study—only a small fraction of eQTLs in blood also affect protein levels, but our results could also reflect targeting of the SOMAmer to a specific ABO protein isoform^25^. Unfortunately, further isoform information for BGAT was not available in the original study.

## Discussion

Identification of genes whose changes in expression are causally linked to a phenotype is crucial for understanding the mechanisms behind complex traits. While several methods exist that infer causal relationships between two phenotypes, these rely on a set of assumptions that are often violated when gene expression is the exposure. Specifically, the presence of LD and pleiotropy between the genetic variants chosen as IVs are the main cause of violations of such assumptions^17–19,28^. Here we interrogated a large gene-expression dataset and showed that the eQTLs of a gene, which can be used as IVs, are very likely to be in LD, but not overlapping, with eQTLs of other genes, indicating that potential sources of pleiotropy in transcriptome-wide MR analyses are likely to come from variants in LD with the IVs.

We therefore developed MR-link, a causal inference method that is robust to unobserved pleiotropy. Our in silico results show that MR-link has the best discriminative ability compared to all other MR methods we tested, as well as to the Bayesian colocalization method *coloc*. MR-link jointly models the outcome using jointly significant eQTLs as IVs, combined with variants in LD, to correct for all potential sources of pleiotropy. To our knowledge, this approach has never been used in a causal inference method.

We applied MR-link to real data by applying it to LDL-C cholesterol measurements and eQTLs derived from blood, cerebellum and liver. This identified known and previously unknown causal genes within and outside GWAS loci. For example, in liver we identified the well-known negative causal relationship between expression of *SORT1* in liver and LDL-C^41–43^. In liver, and suggestively in blood, we detected a causal effect for *PVRL2*, a gene located in the *APOE* locus. While a role for this gene is mostly known for immune and endothelial cells and in the context of atherosclerosis^45,47^, our results indicate that regulation of expression of this gene in both blood and liver causally affects LDL-C levels. Given its established role in atherogenesis, *PVRL2* has been proposed as a potential therapeutic target for atherosclerosis. Our study indicates that such strategies should not only take into account the effect on atherosclerotic plaques, but also consider the hepatic function of *PVRL2* in regulating plasma LDL-C levels in humans.

All the genes identified in the analyses that used eQTLs from blood were different from those identified using eQTLs from liver. While this is partly due to statistical power, as the BIOS cohort is more than 20 times larger than the GTEx cohort used to derive eQTLs in liver, this may also be related to tissue-specific mechanisms. We expect that causal genes found in whole blood will affect LDL-C through pathways that signal for lipid changes or regulate lipid binding to erythrocytes, as hypothesized for the *ABO* gene, whereas genes found in liver are more likely to be involved in lipid metabolism^50,51^.

MR-link has several advantages over other recent MR methods developed to overcome bias from LD and pleiotropy^17,23^. First, MR-link can model unobserved pleiotropy, whereas sources of pleiotropy need to be specified in multivariate MR methods. This is particularly important because sources of pleiotropy may be context-dependent and may arise from a phenotype other than those being measured in a cohort^14,34^. Second, MR-link can derive robust causal estimates even when only one or two IVs are available. The majority of genes tested in our large eQTL dataset have fewer than three IVs (68%), which makes it impossible for MR-PRESSO, MR-Egger, and LDA-MR-Egger to make causal estimates^17–19^.

One of the MR-link assumptions is that the IVs affect the outcome only through the exposure, conditional on the unmeasured pleiotropic effect. This assumption is violated when the IVs of the exposure and of the pleiotropic effect are fully overlapping. This assumption must not be violated when a single IV is available, but can be relaxed when multiple IVs are used in the model, as the relative effects of the IVs help to discriminate between a true causal effect and a pleiotropic effect, similar to multivariable Mendelian randomization methods^22^. In the case of multiple IVs that are fully overlapping, we have shown that MR-link has an increased FPR, yet still maintains higher power compared to other MR-methods and superior discriminative ability compared to coloc.

The application of MR-link is not restricted to gene expression or proteomics datasets; it can also be applied to other molecular layers that are known to have a similar genetic architecture to gene expression, such as metabolites. Given the increases in sharing of summary statistics from functional genomics QTL studies, coupled with the development of very large biobanks such as the UK biobank, the Estonian Biobank, the Lifelines cohort study, and the Million Veteran Program cohort^33,52–54^, we foresee many opportunities for applications of MR-link to individual-level data for the identification of the molecular mechanisms underlying complex traits. Of note, while we have limited our simulations to quantitative traits as an outcome in this paper, MR-link could be applied to binary traits such as human diseases. However, we have not investigated its performance in detail for binary outcome phenotypes. Furthermore, as for all MR studies, our method can be applied to populations of any ethnicity, provided that the summary statistics of the exposure are derived from a population that is ethnically-matched with the outcome cohort.

We foresee that many causal relationships will be discovered if highly powered causal inference methods such as MR-link are applied to many human traits. This could make it possible to build extensive causal networks similar in size and complexity to metabolic networks of small molecules, which would provide valuable insights into the mechanisms behind human traits and diseases.

## Methods

### BIOS consortium cohort genotype and expression analysis

We used genotype and expression measurements on 3746 Dutch individuals from the Biobank-based Integrative Omics Study (BIOS; http://www.bbmri.nl/acquisition-use-analyze/bios/), a collection of six different data cohorts: Lifelines DEEP^55^, Prospective ALS Study Netherlands^56^, Leiden Longevity Study^57^, Netherlands Twin Registry^58^, The Cohort on Diabetes and Atherosclerosis Maastricht^59^, and the Rotterdam Study^60^. All cohorts from the BIOS consortium were approved by their ethical committees, as follows: the LLDEEP was approved by the medical ethics committee of the University Medical Center Groningen; the Prospective ALS Study Netherlands was conducted with the approval of the institutional review board of the University Medical Centre Utrecht; the Leiden Longevity Study was approved by the Medical Ethical Committee of the Leiden University Medical Center; the Netherlands Twin Registry was approved by Central Ethics Committee on Research Involving Human Subjects of the VU University Medical Center, Amsterdam, an Institutional Review Board certified by the US Office of Human Research Protections (IRB number IRB-2991 under Federal-wide Assurance-3703; IRB/institute codes, NTR 03-180); the Rotterdam Study was approved by the institutional review board (Medical Ethics Committee) of the Erasmus Medical Center and by the review board of The Netherlands Ministry of Health, Welfare and Sports; the CODAM study was approved by the medical ethics committee of Maastricht University. An informed consent form was obtained from all the participants. Genotyping was performed separately per cohort (see references). All combined genotypes were imputed to the Haplotype reference consortium dataset^61^ using the Michigan imputation server^62^. We retained only biallelic SNPs and confined our analyses to variants with minor allele frequency (MAF) > 0.01, Hardy–Weinberg equilibrium (HWE) *p* value >10^−6^ and an imputation quality RSQR > 0.8. A genetic relationship matrix (GRM) was derived based on LD-pruned genotypes using the Plink 1.9 command --*indep 50 5 2*, and one individual was kept from all pairs of individuals that had a GRM value > 0.1 using the --*rel-cutoff* Plink 1.9 command^31^. Population outliers were identified using a principal component analysis of the GRM, and individuals more distant than three standard deviations from the mean of principal component 1 and principal component 2 were removed.

RNA-seq gene-expression quality control and processing are the same as those of Zhernakova et al.^13^. RNA extracted from whole blood was paired-end sequenced using the Illumina HiSeq 2000 instrument. RNA-seq read alignment was performed using STAR (version 2.3.0e)^63^. During alignment, variants with MAF < 0.01 from the Genome of the Netherlands were masked^64^. Gene expression was quantified using HTSeq (version v0.6.1p1)^65^. Samples with < 80% of reads mapping to exons were considered of low quality and removed. Samples were also removed if they had < 85% of mapped reads, or if they had a median 3′ bias larger than 70% or smaller than 45%. To further account for unobserved confounders, the expression matrix was corrected for the first 25 principal components as well as 5′ bias, 3′ bias, GC content, intron base-pair percentage, and sex following the procedure of Zhernakova et al.^13^. After genotype and expression quality control filters, 3503 individuals with expression data of 19,960 transcripts and genotype information of 7,838,327 SNPs were available for analyses. In this set, 57% were female and the average age was 52.8 years (±16.0 Stand. Dev.). eQTL association analysis was performed for SNPs located ±1.5 Mb of the transcript using Plink 1.9 and the --*assoc* command^31^. For 13,778 genes, at least one eQTL at *p* < 5 × 10^−8^ was identified, and those genes were used for all the analyses described in this manuscript.

We quantified how many genetic variants are necessary to explain gene expression using a conditional joint analysis approach. We identified jointly significant eQTLs by applying GCTA-COJO (v1.26.0)^26^ to eQTL summary statistics, using the BIOS cohort as LD reference panel, and selecting jointly significant variants that showed a *p* < 5 × 10^−8^ in this analysis step. To infer how often eQTLs are shared between genes, we assessed the percentage of genes with top eQTLs (or jointly significant variants) that have LD *r*^2^ > 0.99. We used the *r*^*2*^ > 0.5 threshold to see how often eQTL variants were in LD with each other.

We performed statistical fine-mapping of all genes using the FINEMAP v1.3.1 program^27^. First, we searched for associated eQTL variants (*p* < 5 × 10^−8^) in the *cis*-associated region. We then padded the associated regions with 100 kb and only looked for variants in this extended region. FINEMAP requires the same number of individuals across all variants, therefore we analyzed only the genes with the associated variants available in all subcohorts. We ran FINEMAP on these genes with the --*sss* option, using LD computed with Plink v1.9, with the --*r* command. Furthermore, genes were not run if they had less than 25 variants available in the region, or if a combination of variants led to an invalid posterior probability, leaving 13,276 genes which were successfully fine-mapped.

FINEMAP provides several configurations of statistically fine-mapped variants, along with their posterior probability of being causal. Studies that identify causal variants usually use a high posterior inclusion probability of multiple causal variant configurations to make sure the causal variant is captured in analysis. In MR studies it is not necessary to identify true causal variants, as the IV only needs to explain the exposure signal the best. In our analysis of LD between FINEMAP variants, we have therefore only considered the most likely configuration identified by FINEMAP, as these variants better explain the exposure variation.

### Lifelines cohort genotype data and LDL-C levels

Lifelines is a multi-generational cohort study of 167,000 individuals from the north of The Netherlands. It was approved by the medical ethics committee of the University Medical Center Groningen and conducted in accordance with Helsinki Declaration Guidelines. All participants signed an informed consent form prior to enrollment. A subset of 13,436 Lifelines samples were genotyped with the cytoSNP array and underwent the quality control steps described in Scholtens et al.^33^: Genotyped variants were retained based on three criteria: MAF > 0.001, HWE *p* > 10^−4^, and a genotyping call rate > 0.95. After genotype quality control, samples were imputed using the Genome of the Netherlands reference panel^64^ and Minimac version 2012.10.3^66^. Variants were further excluded if they were of bad imputation quality (RSQR < 0.3), showed deviation from HWE (*p* < 10^−6^), or if they were absent in the set of quality controlled genotyped and imputed variants of the BIOS cohort.

Low-density lipoprotein cholesterol (LDL-C) was estimated using the Friedewald equation^67^, based on triglycerides, high-density lipoprotein, and total cholesterol levels^33^. Total cholesterol levels of individuals who were prescribed cholesterol-lowering medication were divided by 0.8 prior to calculating LDL-C. Individuals with >4.52 mmol per liter total triglycerides were removed^67^. In addition, LDL-C levels were corrected for age, age squared, and sex. After genotype and LDL-C quality control, 12,449 individuals (of which 58.8% were female and the average age was 48.7 years (±11.5 Stand. Dev.)) and 7,336,374 variants remained for analyses. Association analysis for additive effects on LDL-C was performed using linear regression on standardized genotypes, e.g., transforming genotypes into a distribution with mean 0 and variance 1. Summary statistics of this analysis were used to perform MR analyses using the existing MR methods listed in Table 1.

### GTEx download and analysis

We downloaded GTEx version 7 eQTL summary statistics, including non-significant results, from the GTEx website (https://gtexportal.org/home/datasets/)^24^. For every gene with at least one eQTL at *p* < 5 × 10^−8^, conditional analysis using GCTA-COJO was performed to select secondary variants at the same threshold, using the BIOS cohort as an LD reference. This resulted in 4028, 1557, and 1726 genes with at least one jointly significant eQTL for whole blood, liver, and brain (cerebellum) tissues, respectively.

### pQTL summary statistics download and analysis

We downloaded the proteomics summary statistics of Sun et al.^25^ from the GWAS catalog (ftp.ebi.ac.uk/pub/databases/gwas/summary_statistics/SunBB_29875488_GCST005806). We isolated *cis-*regions by selecting variants within +/−1.5 Mb from each transcript. These variants already passed the quality control steps of Sun et al.^25^: (i) INFO score > = 0.7; (ii) minor allele count > = 8; (iii) Hardy–Weinberg equilibrium *p* > = 5 × 10^−6^. For all these variants we used UK10K minor allele frequencies (ftp://ngs.sanger.ac.uk/production/uk10k/UK10K_COHORT/REL-2012-06-02/UK10K_COHORT.20160215.sites.vcf.gz) as this information was not provided in the summary statistics but it is required for GCTA-COJO IV selection. We selected IVs using Lifelines genotypes as an LD reference^33^. To run MR-link, we first selected proteins with significantly (*p* < 5 × 10^−8^) associated variants that were shared between the *cis* summary statistics and the Lifelines cohort. This resulted in 471 proteins with significantly associated variants (*p* < 5 × 10^−8^) that are overlapping with the variants in the Lifelines cohort and for which GCTA-COJO was able to identify IVs.

### Simulation of genotypes

Four hundred and three non-Finnish European individuals were isolated from the 1000 Genomes phase 3 release and used as a starting point for genotype simulation^30^. We simulated genotype data for 25,000 individuals in a chromosomal region (Chromosome 2, 100–105 Mb, human genome build 37) using the HAPGEN2 program (v.2.2.0), combined with interpolated HAPMAP3 recombination rates^68^. The region was then reduced to 1 Mb in length: between 102 Mbp and 103 Mb. Only biallelic SNPs with MAF < 0.01 were retained from simulated genotypes, leaving 3101 variants in this region. Simulated individuals were separated into an outcome cohort of 15,000 individuals, and into an exposure cohort and an LD reference cohort of 5000 individuals each. These cohort sizes were chosen to roughly represent the sizes of BIOS and Lifelines cohorts.

### Simulation of phenotypes

We simulated quantitative phenotypes representing the exposures by randomly selecting SNPs from the simulated genetic region, and subsequently assigning these an effect. Causal SNPs were selected to represent both pleiotropy through LD (Fig. 2b) and pleiotropy through overlap (Fig. 2c). For the scenario of pleiotropy through LD (Fig. 2b), one to ten causal SNPs (subset **s**_E_) for the exposure were randomly selected from the entire simulated genetic region, and the same number of causal SNPs (subset **s**_U_) for the unobserved (pleiotropic) exposure was randomly selected from all SNPs in moderate LD (0.25 < *r*^2^ < 0.95) with SNPs in **s**_E_.

When pleiotropy through overlap was simulated (Fig. 2c), the causal SNPs for the observed and unobserved exposure were selected to be identical: **s**_E_ = **s**_U_. A combination of pleiotropy through overlap and pleiotropy through linkage was simulated by choosing some or all of the SNPs of the unobserved exposure (subset **s**_U_) to be overlapping and some being in LD (0.25 < *r*^2^ < 0.95) with SNPs in **s**_E_.

The mathematical framework for the simulation of phenotypes is as follows. For each selected causal SNP of the exposure (subset **s**_E_), we simulated an effect-size from the uniform distribution *U*(−0.5,0.5) and then simulated the observed exposure **y**_E_ as:

1 $${\mathbf{y}}_{\mathrm{E}} = {\mathbf{X}}{\mathbf{\beta}}_{\mathrm{E}} + {\mathbf{C}} + {\mathbf{\epsilon }}_{\mathrm{E}},$$

where **X** is a genotype matrix of size *n* × *m*, with *n* being the number of individuals (5000) and *m* the number of variants in the region (3101 in the simulated data), **β**_E_ is the vector of effects

$${\mathbf{\beta}}_{{\mathrm{E}},j} = \left\{ {\begin{array}{*{20}{c}} { \sim U\left( { - 0.5,0.5} \right)} & {{\mathrm{if}}\,j \in {\mathbf{s}}_{\mathrm{E}}} \\ 0 & {{\mathrm{otherwise}}} \end{array}} \right.,\forall j \in \{ 1, \ldots ,m\}$$, and ***C*** ~ *N*(0,0.5)^*n*^ is an *n*-vector of independent scalar draws from *N*(0,0.5), representing a cohort-specific confounder value per individual. Finally, ${\mathbf{\epsilon }}_{\mathrm{E}} \sim N\left( {0,1} \right)^n$ is an *n*-vector of the measurement error of the exposure. Similarly, the unobserved exposure **y**_U_ was simulated as:

2 $${\mathbf{y}}_{\mathrm{U}} = {\mathbf{X}}{\mathbf{\beta}}_{\mathrm{U}} + {\mathbf{C}} + {\mathbf{\epsilon }}_{\mathrm{U}},$$

where **β**_U_ is the vector of effects defined as: $${\mathbf{\beta}}_{{\mathrm{U}},j} = \left\{ {\begin{array}{*{20}{c}} { \sim U\left( { - 0.5,0.5} \right)} & {{\mathrm{if}}\;j \in {\mathbf{s}}_{\mathrm{U}}} \\ 0 & {{\mathrm{otherwise}}} \end{array}} \right.,\forall \;j \in \{ 1, \ldots ,m\}$$, **s**_U_ is the selection of SNPs for the unobserved exposure and ${\mathbf{\epsilon }}_{\mathrm{U}}$ are measurement errors distributed as ${\mathbf{\epsilon }}_{\mathrm{E}}$. The outcome phenotype **y**_o_ was then simulated as a linear combination of the observed and unobserved exposures:

3 $${\mathbf{y}}_{\mathrm{O}} = {\mathbf{y}}_{\mathrm{E}}b_{\mathrm{E}} + {\mathbf{y}}_{\mathrm{U}}b_{\mathrm{U}} + {\mathbf{C}} + {\mathbf{\epsilon }}_{\mathrm{O}},$$

where the causal effect of interest is parameterized per simulation run as $b_{\mathrm{E}} \in \{ 0,0.05,0.1,0.2,0.4\}$ and the (unknown) pleiotropic effect is the parameter $b_{\mathrm{U}} \in \left\{ {0,0.4} \right\}$ reflecting absence and presence of a pleiotropic effect in a locus. Again, the measurement error ${\mathbf{\epsilon }}_{\mathrm{O}}$ is drawn from *N*(0,1)^*n*^.

The genetic variants of the exposures (**s**_E_, **s**_**U**_) and their effect sizes **β**_E_, **β**_U_ were drawn and used in both cohorts (exposure and outcome), while the other random variables **C**, ${\mathbf{\epsilon }}_{\mathrm{U}},{\mathbf{\epsilon }}_{\mathrm{E}},{\mathbf{\epsilon }}_{\mathrm{O}}$ were randomly drawn in a cohort-specific manner. Since our model was built to account for unobserved pleiotropy, the observed and unobserved exposure were used to generate the outcome phenotype as in Eq. (3), but only the outcome phenotypes and the summary statistics of the (observed) exposure phenotype were used in the causal inference analysis.

### Simulation parameters and scenarios

We simulated 1500 runs per scenario, each with a unique outcome (O) and two exposures (E and U). The scenarios differed in the number of causal SNPs (which varied from one to ten for both the observed and unobserved exposure), the strength of the causal relationship of interest (varied from no causal effect up to a large effect ($b_{\mathrm{E}} \in \left\{ {0,0.05,0.1,0.2,0.4} \right\}$) and the presence (*b*_U_ = 0.4) or absence ((*b*_U_ = 0.0) of the pleiotropic effect. This resulted in 10 × 5 × 2 = 100 different scenarios.

In certain cases, an estimate cannot be made by an MR method, for instance when insufficient IVs are identified or a solution is not found in the estimation method. As a result, there are sometimes fewer estimates than expected in the final results. To ensure the stability of our FPR and power estimates, we have only reported results for a MR method in a specific scenario if we had more than 100 estimates out of the 1500 simulated runs.

### Instrumental variable selection

IV selection can be difficult when there is LD between association signals. In simulations, we used two IV selection techniques: GCTA-COJO^26^ and *p* value clumping, using standard settings of Plink 1.9 except for the *r*^2^ threshold, which was set to 0.1^31^. Both selection methods used a *p* value threshold of *p* < 5 × 10^−8^. When selecting IVs for BIOS and GTEX, we only used the GCTA-COJO technique.

### MR-link

MR-link is a method for causal inference that is robust to the presence of LD and unobserved pleiotropy. It is an MR approach that requires individual-level data from the outcome cohort and summary statistics (effect sizes, standard errors and MAFs) from an exposure. Conceptually, MR-link jointly models a known exposure with SNPs that are in LD with the exposure IVs (tag-SNPs). Tag-SNPs are used to account for the unobserved pleiotropic effect present in a locus.

We defined our model in the following manner. Let **X** be a genotype matrix of *n* × *m* where *n* is the number of individuals in the outcome study and *m* are all the SNPs in a *cis*-region around the transcript (±1.5 Mb of the transcript), in which SNPs at indices **s**_E_ are the causal genetic variants (IVs) for the exposure E. If we define the exposure E and the unobserved (pleiotropic) exposure U as in Eqs. (1) and (2), then the outcome phenotype **y**_o_ from Eq. (3) can be represented as a function of E and U with the following equation:

4 $${\mathbf{y}}_{\mathrm{O}} = {\mathbf{X}}{\mathbf{\beta }}_{\mathrm{E}}b_{\mathrm{E}} + {\mathbf{X}}{\mathbf{\beta }}_{\mathrm{U}}b_{\mathrm{U}} + {\mathbf{C}}_{\mathrm{O}} + {\mathbf{\epsilon }}_{\mathrm{O}},$$

where *b*_E_ is the causal effect of interest of the exposure on the outcome, *b*_U_ is the causal effect of the unobserved exposure, **C**_o_ is a *n*-vector of independent scalars representing specific confounder per individual and ${\mathbf{\epsilon }}_{\mathrm{O}}$ is the measurement error of the outcome. In the hypothetical case that the genetic effects for both the exposure E and the pleiotropic exposure U are known, we can estimate *b*_E_ by solving Eq. (4) in an analysis that is similar to multivariate MR^22^. In a real-world scenario, only the IV(s) for the exposure are known, while the variants that contribute to the unobserved (pleiotropic) exposure and their effect on the outcome are unknown.

Under Eq. (4), MR-link relies on the assumption that SNPs on **s**_E_ influence the outcome **y**_O_ only through their effect on **y**_E_, when conditioning on **s**_U_.

MR-link uses the following procedure to estimate causal effects:

1. A selection ${\hat{\mathbf{s}}}_{\mathrm{E}}$ of IVs for the exposure and conditional effect sizes $\widehat {\mathbf{\beta }}_{\mathrm{E}}$ for these IVs are determined using the GCTA-COJO method^26^. A vector of effect sizes $\widehat {\mathbf{\beta }}_{\mathrm{E}}$ for all SNPs in the region is thus defined as: $$\widehat {\mathbf{\beta }}_{{\mathrm{E}},j} = \left\{ {\begin{array}{*{20}{c}} { \ne 0} & {{\mathrm{if}}\,j \in {\hat{\mathbf{s}}}_{\mathrm{E}}} \\ 0 & {{\mathrm{otherwise}}} \end{array}} \right.,\forall j \in \{ 1, \ldots ,m\}$$.
2. All SNPs in LD 0.1 < *r*^2^ < 0.99 with the exposure IVs are potential tag-SNPs. These variants are iteratively pruned for high LD so that tag-SNPs, **s**_T_, are always *r*^2^ < 0.95 with each other in order to reduce collinearity and computation time.
3. The following equation is solved for *b*_E_ using ridge regression:
  5 $$y_O = \left( {\begin{array}{*{20}{c}} \vdots & \vdots \\ {\frac{{{\mathbf{X}}\widehat {\mathbf{\beta }}_{\mathrm{E}}}}{{m_{\mathrm{E}}}}} & {\frac{{{\mathbf{X}}_{\mathrm{T}}}}{{\surd m_{\mathrm{T}}}}} \\ \vdots & \vdots \end{array}} \right)\left( {\begin{array}{*{20}{c}} {b_{\mathrm{E}}} \\ \vdots \\ {{\mathbf{\beta }}_{\mathrm{U}}b_{\mathrm{U}}} \\ \vdots \end{array}} \right) + {\it{\epsilon }},$$
  where **X**_T_ is the genotype matrix of the outcome containing only tagging variants as defined in step (2), *m*_T_ is the number of tagging variants and is used to normalize for the number of tags in the region, and *m*_E_ represents the number of IVs selected by the selection method and is a parameter used to remove the dependency of the model on the number of IVs. The resulting coefficient vector contains the causal effect of interest *b*_E_, and the vector **β**_U_*b*_U_ of length *m*_T_ is a nuisance parameter that captures pleiotropic effects.
  Because individual-level data of the outcome is modeled by MR-link, MR-link does not use any summary statistics of the outcome.

We also considered solving the Eq. (5) using ordinary least squares (OLS). However, due to the multicollinear nature of the $$\left( {\begin{array}{*{20}{c}} \vdots & \vdots \\ {\frac{{{\mathbf{X}}\widehat {\mathbf{\beta }}_{\mathrm{E}}}}{{m_{\mathrm{E}}}}} & {\frac{{{\mathbf{X}}_{\mathrm{T}}}}{{\surd m_{\mathrm{T}}}}} \\ \vdots & \vdots \end{array}} \right)$$ matrix, this approach leads to very low detection power (Supplementary Figs. 6–9; Supplementary Data 2–4, 7, and Supplementary Note 1). We therefore applied ridge regression to solve the equation and determined a T statistic and subsequent Wald test two-sided *p* value for ridge regression^69^. Due to the over-conservative nature of the resulting *p* value in simulations and real data (Supplementary Figs. 6–8, 10; Supplementary Data 2–4, 7, and Supplementary Note 1), we calibrated the *p* value distribution of each different scenario by fitting a beta distribution to null estimates to derive the final *p* values (Supplementary Note 1). When we report results for MR-link, it is these calibrated *p* values that we are referring to.

### Mendelian randomization analyses

Causal relationships were estimated with MR-link and four other existing methods: Inverse variance weighting (IVW)^28^, LDA-MR-Egger regression^17^, MR-Egger regression^18^, and MR-PRESSO^19^. All methods were (re-)implemented in Python and compared to present equal results when compared with their original implementation. The corresponding code is available at https://github.com/adriaan-vd-graaf/genome_integration.

The IVW method is a weighted meta-analysis of causal estimates from single IVs. Specifically, a causal estimate *b*_i_ for an IV i is estimated as $b_{\mathrm{i}}^\prime = \frac{{\beta _{{\mathrm{E}},i}^\prime }}{{\beta _{{\mathrm{O}},i}^\prime }}$, where *β*′_O,*i*_ is the marginal effect of SNP *i* on the outcome and *β*′_E,*i*_ is the marginal effect of the exposure. For the estimation of the causal effect, single IV causal estimates are combined using weights proportional to the inverse variance of such estimates using the two-terms definition of standard error: $se\left( {b_i^\prime } \right) = \sqrt {\frac{{se\left( {\beta _{{\mathrm{O,}}i}^\prime } \right)^2}}{{\beta _{{\mathrm{E}},i}^{\prime 2}}} + \frac{{\beta _{{\mathrm{O}},i}^{\prime 2}se\left( {\beta _{E,i}^\prime } \right)^2}}{{\beta _{{\mathrm{E}},i}^{\prime 2}}}}$ as following Burgess and Thompson^70^.

MR-Egger regression adjusts for average pleiotropy by fitting a weighted linear regression between the exposure SNP-effects and the outcome SNP-effects^18^. It assumes that <50% of the variants have a pleiotropic effect. MR-Egger can be applied when three or more instruments are available.

LDA-MR-Egger is similar to MR-Egger but also recognizes LD. LDA-MR-Egger can only be used when LD information between the IVs is available^17,18^.

MR-PRESSO is a method of causal inference that implements an approach to identify and remove outliers from the IVW framework^19^. It assumes that <50% of the variants have a pleiotropic effect. MR-PRESSO is unable to adjust for the presence of pleiotropy if fewer than three IVs are available, of if fewer than two IVs are left after outlier correction.

We applied these four methods to both simulated and real data. For real data, we used the LDL-C full GWAS summary statistics derived from the association carried out in the Lifelines study, as described above.

Prior to MR analyses, for each IV, we select the allele with positive effect on the exposure.

### Colocalization analyses

We have run colocalization analyses on the simulated data using the R package *coloc* v4, git commit *6f3cbb1e5e90f07de772339d6e4af362140affc3*, specifically its *coloc.abf()* function for the original *coloc* functionality and the *coloc.signals()* function for the masked (*coloc-masked*) and conditional (*coloc-cond*) estimates^29,32^. We used marginal effect sizes, standard errors and the MAFs as input that were calculated separately for the exposure and outcome. The LD for the conditional and masked *coloc* analysis was derived from the simulated reference cohort. For original *coloc*, we used the H4 test statistic of the *coloc.abf()* function as our result metric, which provides the posterior probability of sharing of the causal variants between the two traits being tested. For the *coloc-cond* and *coloc-masked* results, we have used the maximum PP4 reported by the *coloc.signals()* function, as this represents the largest posterior probability that a causal variant is shared between traits. We compared the discriminative ability of all *coloc* variations with that of MR-link using (i) false-positive rate and power when using a PP4 > 0.9 to declare colocalization and (ii) an area under the curve (AUC) statistic of the receiver operator curve, where scenarios with *b*_E_ = 0 (null causal effect of the exposure) were considered true negative observations and $b_{\mathrm{E}} \ne 0$ were considered the true positive observations. We determined the AUC using the sklearn library^71^.

### Reporting summary

Further information on research design is available in the Nature Research Reporting Summary linked to this article.

## Supplementary information

Supplementary Information

Peer Review

Reporting Summary

Description of Additional Supplementary Files

Supplementary Data 1

Supplementary Data 2

Supplementary Data 3

Supplementary Data 4

Supplementary Data 5

Supplementary Data 6

Supplementary Data 7

## Data Availability

Individual-level data of Lifelines cohorts are available to all bona-fide researchers upon request to the Lifelines biobank (https://www.lifelines.nl/researcher). Individual-level data (genotypes and RNA-seq data) of the BIOS Consortium cohorts can be downloaded by researchers of Dutch Institutes, or analyzed (but not downloaded) by any non-Dutch researcher in a Cloud environment (https://www.bbmri.nl/acquisition-use-analyze/bios). GTEx summary statistics can be downloaded from the GTEx website (https://gtexportal.org/home/datasets). pQTLs summary statistics can be downloaded from GWAS Catalog (ftp.ebi.ac.uk/pub/databases/gwas/summary_statistics/SunBB_29875488_GCST005806/). Simulated data can be recreated using the code at the link provided in Code availability statement. Imputation with the Haplotype Reference Consortium dataset can be done at the following link: https://imputationserver.sph.umich.edu/index.html#!. Raw data used to draw Fig. 3 can be found in Supplementary Data 3.

## Code availability

An implementation of MR-link, the methods to recreate the simulated data, and instructions on usage can be found at https://github.com/adriaan-vd-graaf/genome_integration. This repository also includes implementation of the other MR methods and the *coloc* method used in this paper.
