# Supplementary material for: Mendelian randomization while jointly modeling cis genetics identifies causal relationships between gene expression and lipids
Source: Nat Commun. 2020 Oct 1;11:4930. doi: 10.1038/s41467-020-18716-x (PMC7530717; doi:10.1038/s41467-020-18716-x)
Supplement: Supplementary file 1 — Supplementary Information [file 41467_2020_18716_MOESM1_ESM.pdf]

# SUPPLEMENTARY INFORMATION

**Mendelian randomization while jointly modeling *cis* genetics identifies causal relationships between gene expression and lipids**

van der Graaf et al.

**Supplementary Note 1. Calibration of *p* values resulting from MR-link analysis**

In a preliminary version of MR-link we used Ordinary Least Squares (OLS) to solve equation (5) (**Methods**) (MR-link, OLS). Here we observed that MR-link with OLS implementation had FPR close to expectations (**Supplementary Figure 6, Supplementary Data 2**), but lacked sufficient power to detect a causal effect (power ranged from 0.13 to 0.23 for scenarios with 1 to 10 causal variants and $b_E$ = 0.4). We hypothesized that this was likely due to multicollinearity in the MR-link model. To overcome this issue, we replaced OLS with ridge regression, which is relatively robust to multicollinearity compared to OLS and LASSO regression, especially when $n < m$ as is the often the case in our analyses[1,2]. We used a previously described[3] method to estimate the standard error of the ridge estimate and subsequently derived a T statistic. However, using MR-link with ridge regression implementation resulted in conservative *p* values (**Supplementary Data 2-4, Supplementary Figure 6** and **7**). To ensure that the MR-link ridge test statistic *p* value followed a uniform distribution in the case of the null scenario, we calibrated the *p* values using a beta distribution fit. The beta distribution estimate was made using a Markov Chain Monte Carlo approach, with the parameters of the beta distribution having a uniform prior between 0 and 20. These parameters are estimated using PYMC3 combined with a No-U-Turn sampler, drawing 100,000 samples after tuning for 1,000,000 steps, using two parallel chains[4]. This estimation scheme for the beta distribution parameters is preferable to maximum likelihood (ML) estimates because ML estimates are prone to convergence issues when a distribution is heavily shifted toward one.

In simulations the beta distribution is estimated separately in each null scenario (no causal effect without pleiotropy, no casual effect and pleiotropy through LD) and then applied to the corresponding causal scenarios. The results shown in **Supplementary Figure 6** and **Supplementary Figure 7** demonstrate that calibration successfully restored *p* values to follow the expected distribution. In the application to real data, the estimation of the beta distribution for calibration was done on all observations assuming that

the majority of tests in a transcriptome-wide analysis represent a null causal effect. This concept is similar to that behind the genomic control correction approach used in GWAS studies[5]. When we applied this approach to the results obtained using eQTLs from BIOS and LifeLines individual-level data, we saw similar behavior to that observed in simulations, although slightly inflated compared to what was observed in simulations, likely reflecting a subset of genes with small to moderate causal effects (**Supplementary Figure 8**). All the results presented for simulations and application to real data in the main text refer to MR-link with ridge regression implementation and calibration of *p* values.

It is worth to mention that our calibration procedure in simulations assumes that *p* values used for calibration are homogeneous to the tested scenario, or in other words - they are drawn from the null of the corresponding non-pleiotropic/pleiotropic scenario. In real data, we don't know *a priori* what is the corresponding null scenario for proper calibration, but given our observations in the BIOS cohort, we expect this to be a mixture of non-pleiotropic and pleiotropy through LD. Therefore, we evaluated the impact of calibration by fitting the beta distribution based on the combined null distributions of the non-pleiotropic and pleiotropy through LD simulation scenario. This *p* value calibration approach increases the false positive rates in the pleiotropy through LD scenario, while the false positive rates decreases in the non-pleiotropic scenarios (**Supplementary Figure 9**) (**Supplementary Data 7**). Similar patterns are also seen in power for these scenarios (**Supplementary Figure 9**) (**Supplementary Data 7**). The ordering of significant effects is not affected by *p* value calibration and therefore the same discriminative ability in terms of AUC is retained.

**Supplementary Note 2. BIOS Consortium Members and affiliations**

*Management Team*

Bastiaan T. Heijmans (chair)[1], Peter A.C. ’t Hoen[2], Joyce van Meurs[3], Rick Jansen[5], Lude Franke[6].

*Cohort collection*

Dorret I. Boomsma[7], René Pool[7], Jenny van Dongen[7], Jouke J. Hottenga[7] (Netherlands Twin Register); Marleen MJ van Greevenbroek[8], Coen D.A. Stehouwer[8], Carla J.H. van der Kallen[8], Casper G. Schalkwijk[8] (Cohort study on Diabetes and Atherosclerosis Maastricht); Cisca Wijmenga[6], Lude Franke[6], Sasha Zhernakova[6], Ettje F. Tigchelaar[6] (LifeLines Deep); P. Eline Slagboom[1], Marian Beekman[1], Joris Deelen[1], Diana van Heemst[9] (Leiden Longevity Study); Jan H. Veldink[10], Leonard H. van den Berg[10] (Prospective ALS Study Netherlands); Cornelia M. van Duijn[4], Bert A. Hofman[11], Aaron Isaacs[4], André G. Uitterlinden[3] (Rotterdam Study).

*Data Generation*

Joyce van Meurs (Chair)[3], P. Mila Jhamai[3], Michael Verbiest[3], H. Eka D. Suchiman[1], Marijn Verkerk[3], Ruud van der Breggen[1], Jeroen van Rooij[3], Nico Lakenberg[1].

Data management and computational infrastructure

Hailiang Mei (Chair)[1][2], Maarten van Iterson[1], Michiel van Galen[2], Jan Bot[1][3], Dasha V. Zhernakova[6], Rick Jansen[5], Peter van ’t Hof[1][2], Patrick Deelen[6], Irene Nooren[1][3], Peter A.C. ’t Hoen[2], Bastiaan T. Heijmans[1], Matthijs Moed[1].

*Data Analysis Group*

Lude Franke (Co-Chair)[6], Martijn Vermaat[2], Dasha V. Zhernakova[6], René Luijk[1], Marc Jan Bonder[6], Maarten van Iterson[1], Patrick Deelen[6], Freerk van Dijk[1][4], Michiel van Galen[2], Wibowo

Arindrarto[1][2], Szymon M. Kielbasa[1][5], Morris A. Swertz[1][4], Erik. W van Zwet[1][5], Rick Jansen[5], Peter-Bram ’t Hoen (Co-Chair)[2], Bastiaan T. Heijmans (Co-Chair)[1].

[1] Molecular Epidemiology, Department of Biomedical Data Sciences, Leiden University Medical Center, Leiden, The Netherlands

[2] Department of Human Genetics, Leiden University Medical Center, Leiden, The Netherlands

[3] Department of Internal Medicine, ErasmusMC, Rotterdam, The Netherlands

[4] Department of Genetic Epidemiology, ErasmusMC, Rotterdam, The Netherlands

[5] Department of Psychiatry, VU University Medical Center, Neuroscience Campus Amsterdam, Amsterdam, The Netherlands

[6] Department of Genetics, University of Groningen, University Medical Centre Groningen, Groningen, The Netherlands

[7] Department of Biological Psychology, VU University Amsterdam, Neuroscience Campus Amsterdam, Amsterdam, The Netherlands

[8] Department of Internal Medicine and School for Cardiovascular Diseases (CARIM), Maastricht University Medical Center, Maastricht, The Netherlands

[9] Department of Gerontology and Geriatrics, Leiden University Medical Center, Leiden, The Netherlands

[10] Department of Neurology, Brain Center Rudolf Magnus, University Medical Center Utrecht, Utrecht, The Netherlands

[11] Department of Epidemiology, ErasmusMC, Rotterdam, The Netherlands

[12] Sequence Analysis Support Core, Department of Biomedical Data Sciences, Leiden University Medical Center, Leiden, The Netherlands

[13] SURFsara, Amsterdam, the Netherlands

[14] Genomics Coordination Center, University Medical Center Groningen, University of Groningen, Groningen, the Netherlands

[15] Medical Statistics, Department of Biomedical Data Sciences, Leiden University Medical Center, Leiden, The Netherlands

## Supplementary Figures

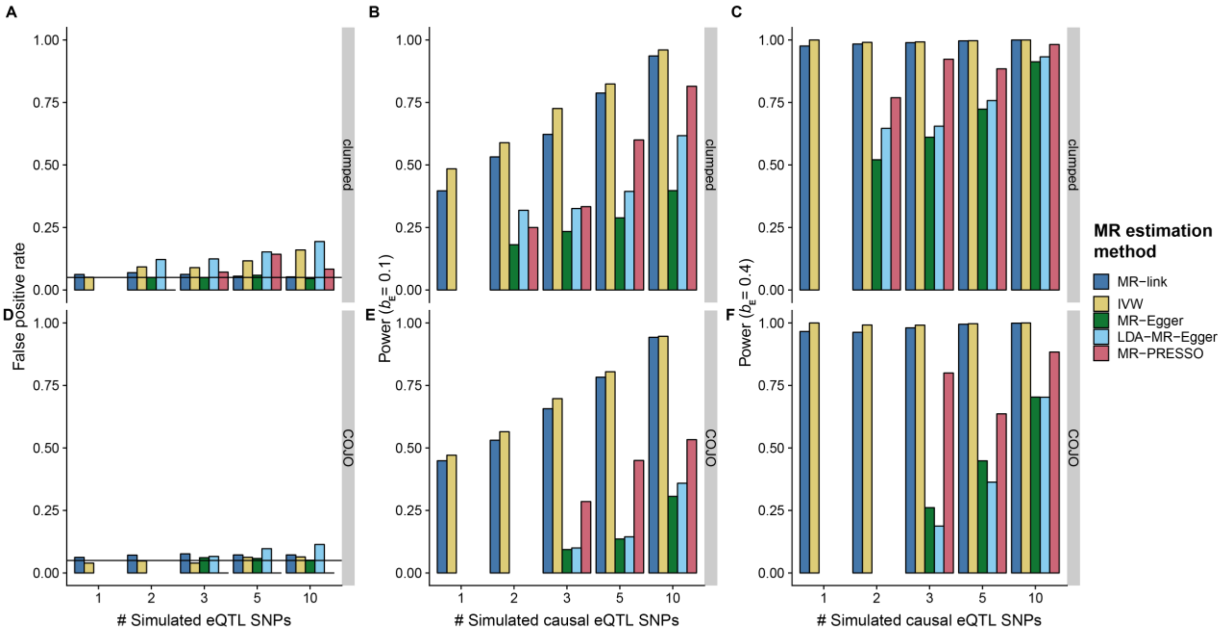


**Supplementary Figure 1. Simulation results depicting Type I error rates (at 0.05 significance) for two different IV selection methods**

Type I error rates (at 0.05 significance) in simulations when two different IV selection methods, *p* value clumping (clumped) (panels **A** to **C**) and GCTA-COJO (COJO) (panels **D** to **F**), were used in a non-pleiotropic scenario ($b_U = 0$) (**Methods**), with both selecting IVs at a threshold of $p < 5x10^{-8}$. (**A, D**) False positive rates in a scenario where no causal relationship is simulated. (**B, E**) Power to detect an effect in a scenario where a small causal effect ($b_E$=0.1) is simulated. (**C, F**) Power to detect an effect in a scenario where a large causal effect ($b_E$=0.4) is simulated. Note that MR-link and IVW are the only MR methods that can derive a causal estimate when just one or two IVs are available. Extended results for these and other scenarios are given in **Supplementary Data 2**.

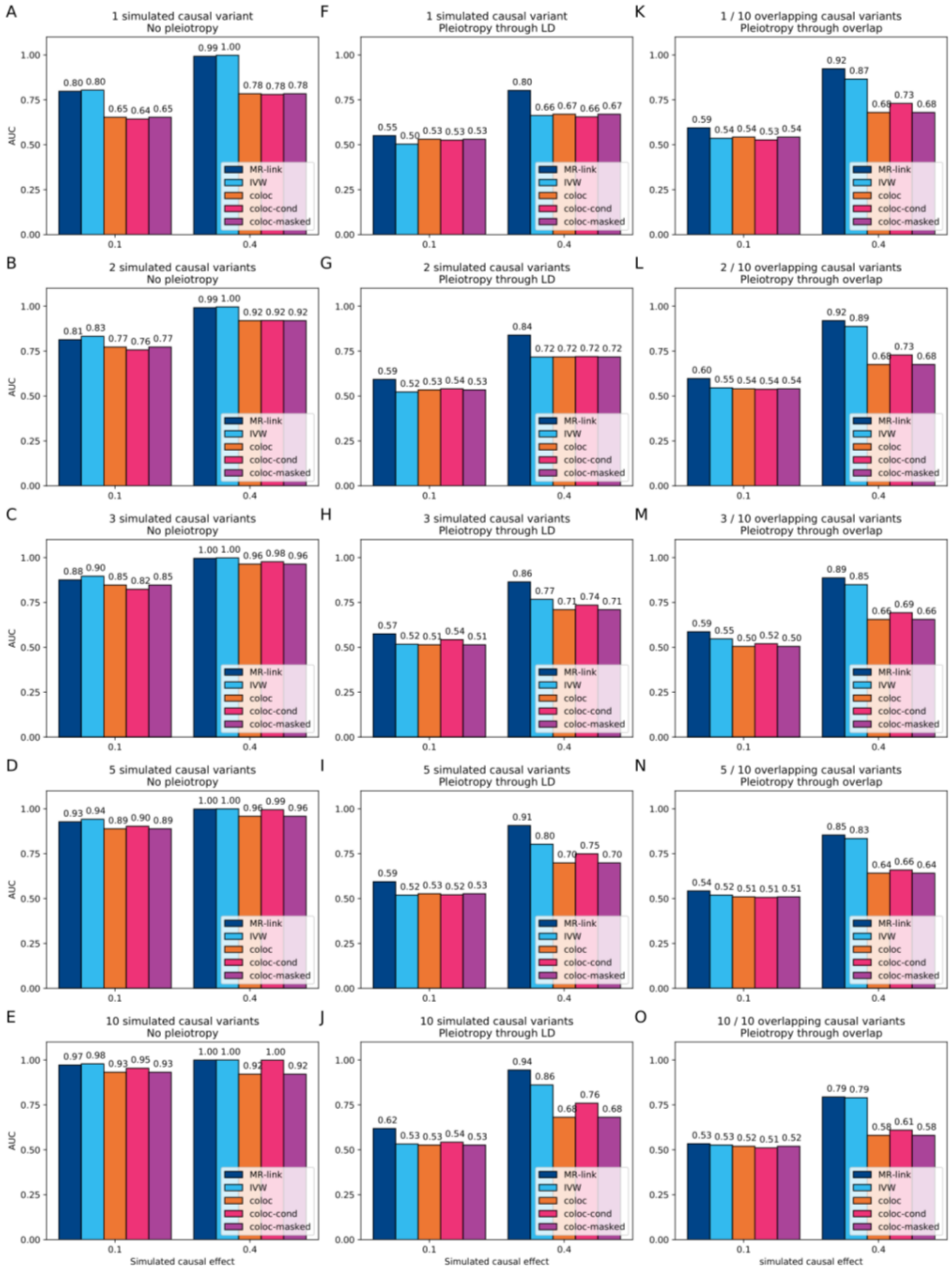


**Supplementary Figure 2. Representation of the discriminative ability of coloc, MR-link and IVW through area under the receiver operator characteristic curve**

The associated area under the receiver operator characteristic curve (AUC) for different simulation scenarios comparing the discriminative performance of MR-link, IVW and three variations of coloc when the observed exposure has a small ($b_E$ = 0.1) or large ($b_E$ = 0.4) causal effect. (**A-E**) Simulation scenarios without pleiotropy and increasing number of causal variants. (**F-J**) Simulation scenarios where pleiotropy through LD is simulated and increasing number of causal variants. (**K-0**) Simulation scenarios of gradually increasing pleiotropy through overlap and 10 causal variants. AUC numbers are rounded. Extended results are presented in **Supplementary Data 5**.

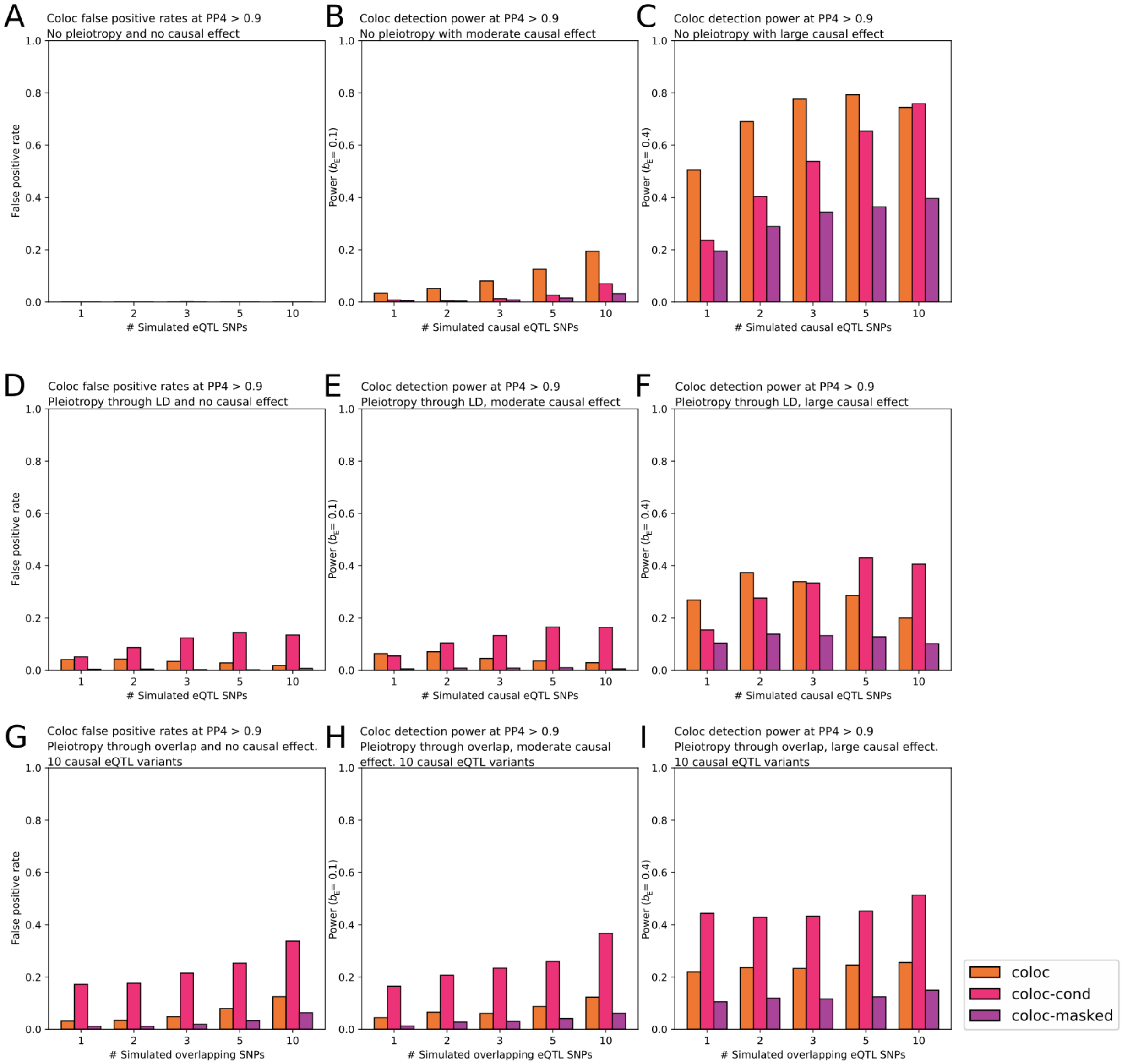


**Supplementary Figure 3. False positive rate and power for coloc methods**

This figure shows detection performance of coloc variations based on simulations representing no pleiotropy (panels **A** to **C**), pleiotropy through linkage disequilibrium (LD) scenarios (panels **D** to **F**) when 1, 3, 5 or 10 causal SNPs were simulated and increasing levels of pleiotropy through overlap combined with 10 causal variants (**G-I**) (**Methods**). (**A, D, G**) False positive rates (at coloc PP4 > 0.9) when no causal effect is simulated. (**B, E, H**) Detection power when a moderate causal effect is simulated ($b_{\mathrm{E}} = 0.1$) (at coloc PP4 > 0.9)). (**C, F, I**) Detection power when a large causal effect is simulated ($b_{\mathrm{E}} = 0.4$) (at coloc PP4 > 0.9). Extended results can be found in **Supplementary Data 6**.

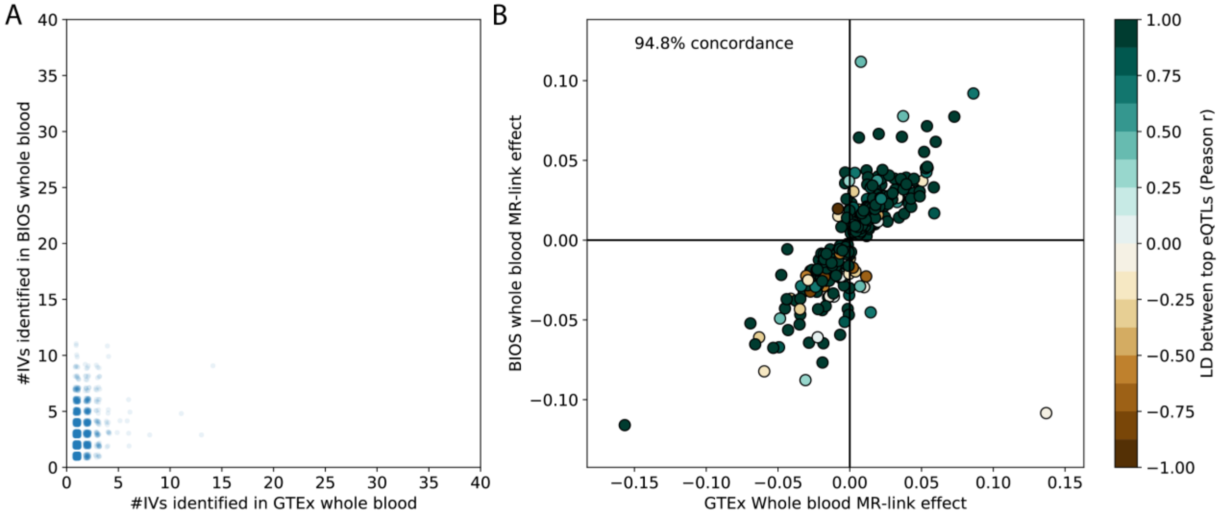


**Supplementary Figure 4. Comparison of IVs and effect-sizes identified when using eQTLs from BIOS and GTEx whole blood**

Panel **A** depicts the number of conditionally independent eQTL variants identified for all the genes with at least one eQTL ($p < 5x10^{-8}$) in both the BIOS and GTEx cohorts. To represent the number of overlapping datapoints, all points are randomly jittered in both the x and y direction and have some transparency. Panel **B** depicts a scatterplot of causal effect sizes identified by MR-link when using GTEx whole blood and BIOS eQTLs to identify causal genes for LDL-C for all the marginally significant ($p < 0.05$) genes detected using the BIOS cohort eQTLs. Colors indicate the Pearson *r* (squared root of linkage disequilibrium (LD)) between the most significant eQTL variants of the gene.

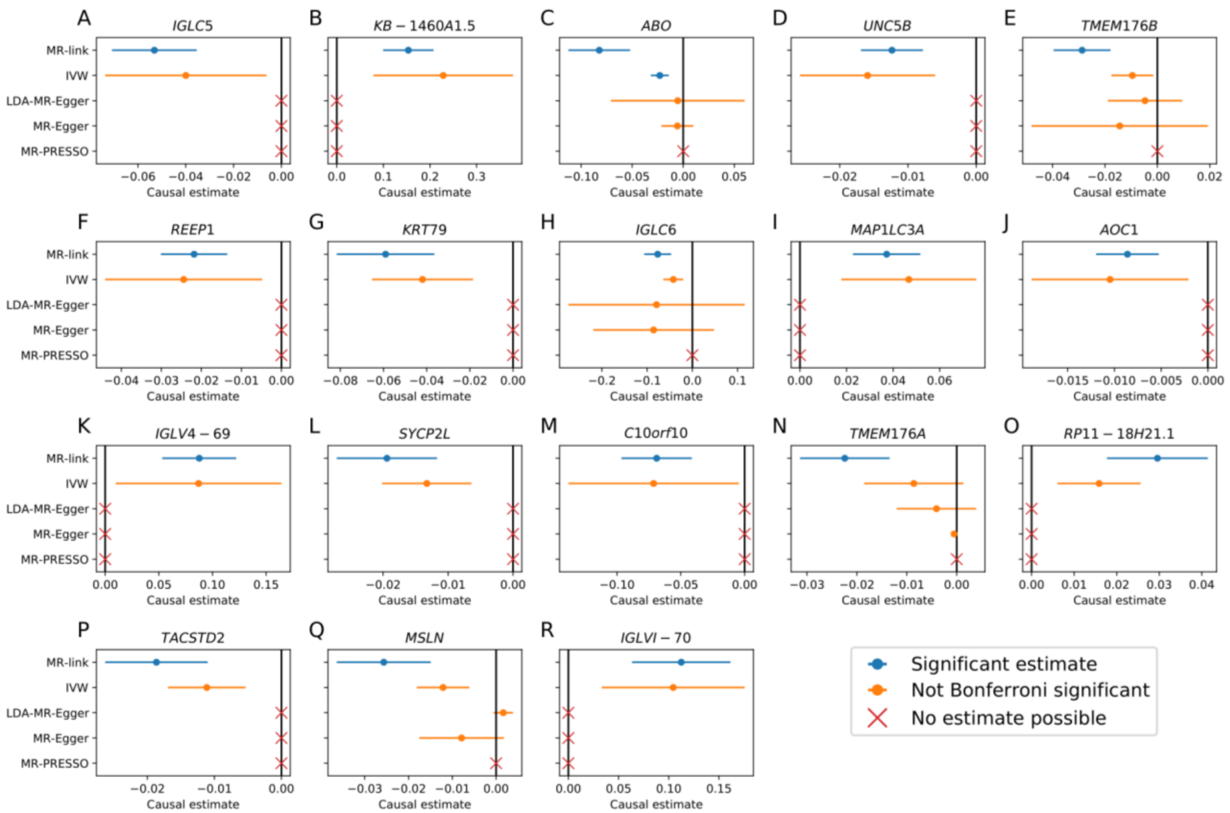


**Supplementary Figure 5. Forest plots of causal effect estimates for the 18 significant genes identified by MR-link using BIOS blood eQTLs**

This figure depicts the estimated causal effect size by the used MR-methods (large dot at the center of each bar) and their 95% confidence interval for the 18 genes (panels **A** to **R**) identified as significant by MR-link using BIOS whole-blood eQTLs and the Lifelines Cohort (N=12,449) (genes are those listed in **Table 2**). Causal effect estimates by MR-PRESSO, MR-Egger and LDA-MR-Egger were only possible for genes with more than two IVs (**C**, **E**, **H**, **N**, **Q**). No estimates were made by MR-PRESSO for any of these 5 genes because it identified too many outliers (**C**, **E H**, **N**, **Q**); these are depicted with a red cross. Full summary statistics of the methods can be found in **Supplementary Table 3.** For MR-link the confidence interval has been estimated based on the standard error corresponding to the calibrated *p* value (**Supplementary Note 1)**.

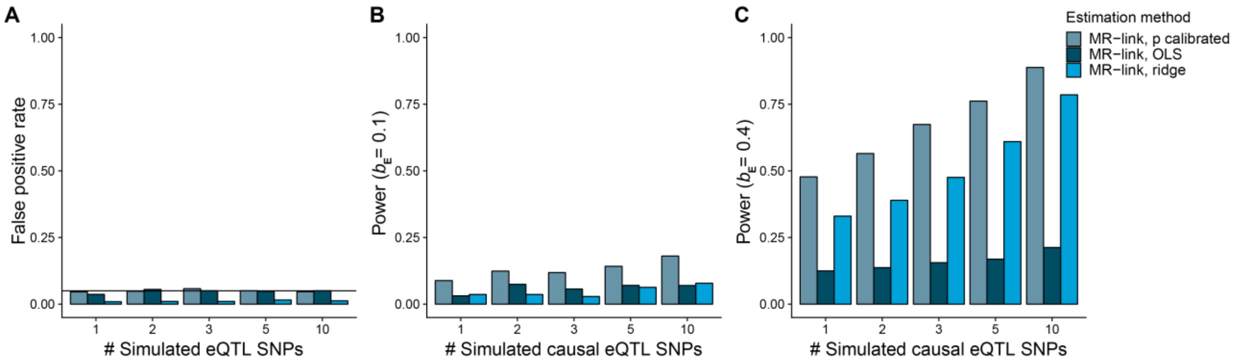


**Supplementary Figure 6. Simulation results using different solvers for MR-link**

Simulation results depicting false positive rates and detection power (at 0.05 significance) for MR-link when different equation solvers are used: OLS, ridge regression, and ridge regression followed by *p* value calibration (**Supplementary Note 1**). Results are from the same pleiotropic scenarios simulated in **Figure 3**. (**A**) False positive rates in a scenario where no causal relationship is simulated. (**B**) Power to detect an effect in a scenario where a small causal effect ($b_E = 0.1$) is simulated. (**C**) Power to detect an effect in a scenario where a large ($b_E = 0.4$) causal effect is simulated.

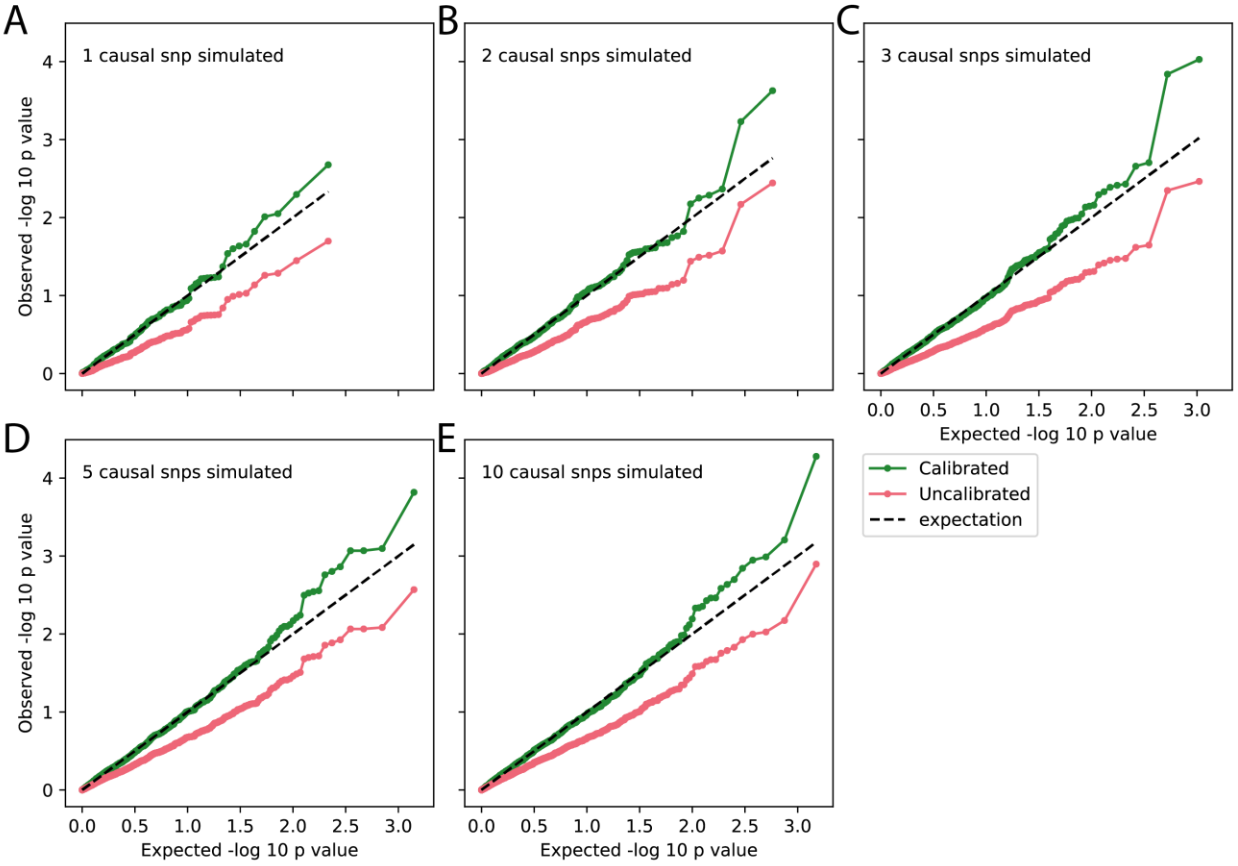


**Supplementary Figure 7. Calibration of *p* values in null scenarios**

Quantile-quantile plot of the p values obtained with MR-link ridge before and after calibration (beta distribution fit, see **Supplementary Note 1**) in the simulation scenario of pleiotropy through linkage disequilibrium (LD) (depicted in **Figure 2B**) with 1 (**A**), 2 (**B**), 3 (**C**), 5 (**D**) and 10 (**E**) simulated causal SNPs . Expected *p* values are calculated as the inverse of the rank of the *p* value sorted by significance. Dashed line is the diagonal.

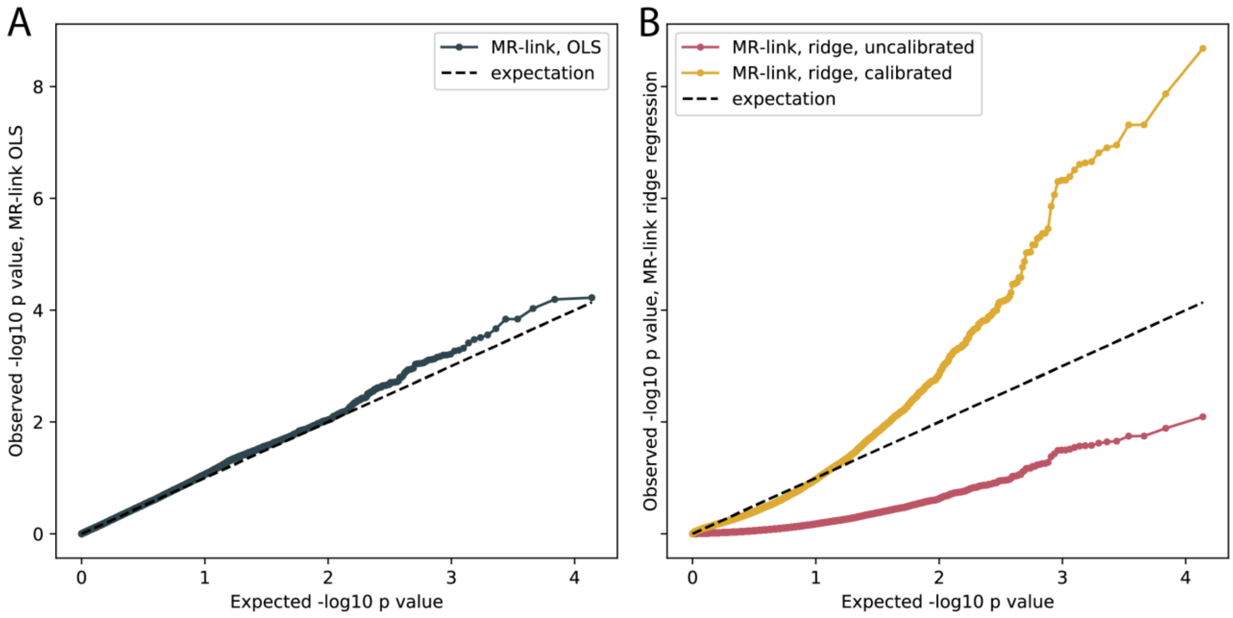


**Supplementary Figure 8. Comparison of MR-link *p* values obtained using the BIOS eQTL data and LDL-C individual data**

*p* values of MR-link obtained after application to LifeLines individual-level data and eQTLs from the BIOS cohort compared to the expected *p* value distribution (dashed line). (**A**) Using ordinary least squares (OLS) regression to solve equation 4 (**Methods**). (**B**) Using ridge regression to solve equation 5 (red) and ridge regression combined with *p* value calibration (yellow) (**Methods**) (**Supplementary Note 1**).

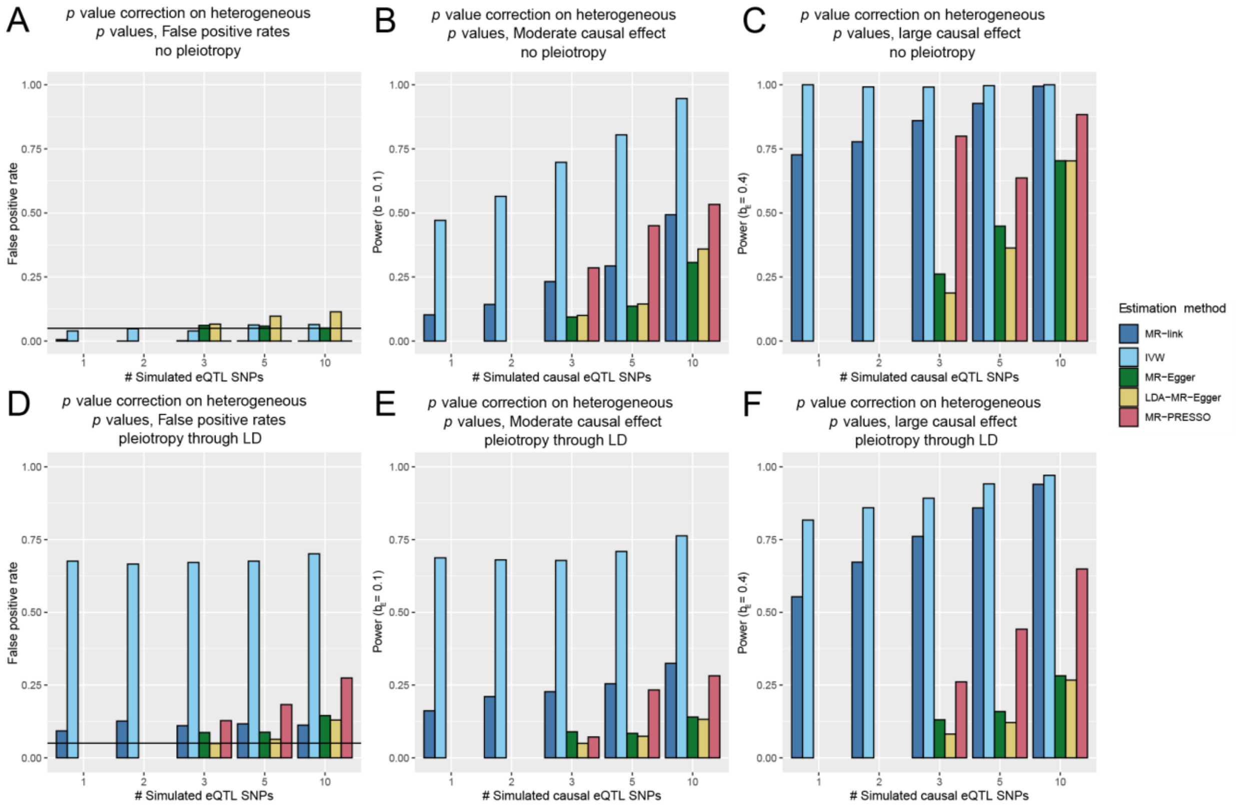


**Supplementary Figure 9. False positive rates and power of MR-link when using different *p* value calibration procedures**

The figure shows performance of MR methods compared to MR-link when *p* values are calibrated on a *p* distribution that is heterogeneous (The scenarios without pleiotropic effect and pleiotropy through linkage disequilibrium combined) (**Supplementary Note 1**). Performance is based on simulations representing no pleiotropy (**A-C**) and pleiotropy through linkage disequilibrium (LD) scenarios (depicted in **Figure 2B**) (**D-F**) when 1, 3, 5 or 10 causal SNPs were simulated (**Methods**). (**A, D**) False positive rates (at alpha 0.05) when no causal effect is simulated. (**B, E**) Detection power when a moderate causal effect is simulated ($b_{\mathrm{E}} = 0.1$) (at alpha 0.05). (**C, F**) Detection power when a large causal effect is simulated ($b_{\mathrm{E}} = 0.4$) (at alpha 0.05). MR methods that had fewer than 100 out of 1,500 estimates in a scenario are not shown (**Methods**). Extended results depicted here can be found in **Supplementary Data 7**.

## Supplementary Tables

**Supplementary Table 1. Full results of the genes that were significant using MR-link and the eQTLs from the BIOS cohort**

| ensg_id | gene_id | estimated beta | SE | calibrated_p | IVs_identified |
|---|---|---|---|---|---|
| ENSG00000254030 | *IGLC5* | -0.05313 | 0.020056 | 2.08E-09 | 1 |
| ENSG00000261087 | *KB-1460A1.5* | 0.153536 | 0.061786 | 1.35E-08 | 1 |
| ENSG00000175164 | *ABO* | -0.08224 | 0.034726 | 4.84E-08 | 4 |
| ENSG00000107731 | *UNC5B* | -0.01235 | 0.005216 | 4.87E-08 | 1 |
| ENSG00000106565 | *TMEM176B* | -0.0287 | 0.012538 | 1.11E-07 | 4 |
| ENSG00000068615 | *REEP1* | -0.02183 | 0.00958 | 1.25E-07 | 1 |
| ENSG00000185640 | *KRT79* | -0.05904 | 0.026144 | 1.54E-07 | 2 |
| ENSG00000222037 | *IGLC6* | -0.07662 | 0.034466 | 2.21E-07 | 3 |
| ENSG00000101460 | *MAP1LC3A* | 0.037236 | 0.016789 | 2.32E-07 | 1 |
| ENSG00000002726 | *AOC1* | -0.00861 | 0.003893 | 2.48E-07 | 1 |
| ENSG00000211637 | *IGLV4-69* | 0.087668 | 0.040044 | 3.1E-07 | 1 |
| ENSG00000153157 | *SYCP2L* | -0.01941 | 0.008981 | 4.09E-07 | 2 |
| ENSG00000165507 | *C10orf10* | -0.06893 | 0.032106 | 4.74E-07 | 1 |
| ENSG00000002933 | *TMEM176A* | -0.02242 | 0.010448 | 4.77E-07 | 3 |
| ENSG00000245954 | *RP11-18H21.1* | 0.029575 | 0.013813 | 5.02E-07 | 2 |
| ENSG00000184292 | *TACSTD2* | -0.01865 | 0.008943 | 8.65E-07 | 2 |
| ENSG00000102854 | *MSLN* | -0.02566 | 0.012603 | 1.39E-06 | 4 |
| ENSG00000253239 | *IGLVI-70* | 0.112435 | 0.058012 | 3.49E-06 | 2 |

In this table we list the full results for the 18 genes that pass significance in the MR-link analysis that used eQTLs from BIOS and the Lifelines cohort genotypes and LDL-C levels (N=12,449). We report the following information per gene: the Ensembl ID (*ensg_id*), gene ID (*gene_id*), causal estimate (*estimated beta*), standard error of the causal estimate (*SE*), 2 sided *p* value of the estimate from MR-link after calibration (*calibrated_p*) (see **Supplementary Note 1** on how the p value was calibrated) and finally the number of IVs identified by GCTA-COJO (*IVs_identified*).

**Supplementary Table 2. Significant genes identified in the MR-link analysis that used GTEx eQTL data**

| tissue | ensg_id | gene_id | estimated_beta | SE | calibrated_p | IVs_identified |
|---|---|---|---|---|---|---|
| Liver | ENSG00000130202.5 | *PVRL2* | 0.318 | 0.052 | 3.24E-14 | 1 |
| Liver | ENSG00000134222.12 | *PSRC1* | -0.085 | 0.018 | 4.17E-09 | 1 |
| Liver | ENSG00000143126.7 | *CELSR2* | -0.099 | 0.024 | 7.04E-08 | 1 |
| Liver | ENSG00000134243.7 | *SORT1* | -0.087 | 0.019 | 6.15E-09 | 1 |
| Whole Blood | ENSG00000204920.6 | *ZNF155* | -0.069 | 0.034 | 9.8E-06 | 2 |
| Whole Blood | ENSG00000134222.12 | *PSRC1* | -0.157 | 0.069 | 1.69E-06 | 1 |

This table lists the full results for the 6 genes found to be significant in the MR-link analysis that used GTEx eQTL summary statistics. It contains the following information per gene: the eQTL tissue (*tissue*), Ensembl ID (*ensg_id*), gene ID (*gene_id*), causal estimate (*estimated_beta*), standard error of the causal estimate (*SE*), 2-sided *p* value of the estimate from MR-link after calibration (*calibrated_p*) (see **Supplementary Note 1** on how the *p* values were calibrated), and finally the number of IVs identified by GCTA-COJO (*IVs_identified*).

**Supplementary Table 3. Results of all other MR methods tested for the 18 genes identified using MR-link and the BIOS cohort**

| ensg_id | gene_id | method | beta | SE | p value | IVs identified |
|---|---|---|---|---|---|---|
| ENSG00000245954 | *RP11-18H21.1* | IVW | 0.016 | 0.005 | 0.000729 | 2 |
| ENSG00000261087 | *KB-1460A1.5* | IVW | 0.228 | 0.072 | 0.001402 | 1 |
| ENSG00000068615 | *REEP1* | IVW | -0.024 | 0.009 | 0.007291 | 1 |
| ENSG00000175164 | *ABO* | IVW | -0.023 | 0.0044 | 1.50E-07 | 4 |
| ENSG00000175164 | *ABO* | MR-Egger | -0.006 | 0.0034 | 2.37E-01 | 4 |
| ENSG00000175164 | *ABO* | MR-PRESSO | NA | NA | NA | NA |
| ENSG00000175164 | *ABO* | LDA-MR-Egger | -0.005 | 0.0055 | 4.36E-01 | 4 |
| ENSG00000002726 | *AOC1* | IVW | -0.010 | 0.004 | 0.007236 | 1 |
| ENSG00000222037 | *IGLC6* | IVW | -0.042 | 0.011 | 9.09E-05 | 3 |
| ENSG00000222037 | *IGLC6* | MR-Egger | -0.086 | 0.014 | 0.103933 | 3 |
| ENSG00000222037 | *IGLC6* | MR-PRESSO | NA | NA | NA | NA |
| ENSG00000222037 | *IGLC6* | LDA-MR-Egger | -0.079 | 0.027 | 0.212663 | 3 |
| ENSG00000184292 | *TACSTD2* | IVW | -0.011 | 0.003 | 8.15E-05 | 2 |
| ENSG00000101460 | *MAP1LC3A* | IVW | 0.0467 | 0.0139 | 7.85E-04 | 1 |
| ENSG00000102854 | *MSLN* | IVW | -0.0121 | 0.0029 | 3.81E-05 | 4 |
| ENSG00000102854 | *MSLN* | MR-Egger | -0.0079 | 0.0019 | 5.45E-02 | 4 |
| ENSG00000102854 | *MSLN* | MR-PRESSO | NA | NA | NA | NA |
| ENSG00000102854 | *MSLN* | LDA-MR-Egger | 0.0016 | 0.0005 | 7.38E-02 | 4 |
| ENSG00000153157 | *SYCP2L* | IVW | -0.013 | 0.003 | 7.59E-05 | 2 |
| ENSG00000107731 | *UNC5B* | IVW | -0.016 | 0.005 | 0.001 | 1 |
| ENSG00000211637 | *IGLV4-69* | IVW | 0.087 | 0.035 | 0.014 | 1 |
| ENSG00000253239 | *IGLVI-70* | IVW | 0.104 | 0.034 | 0.0020 | 2 |
| ENSG00000185640 | *KRT79* | IVW | -0.0419 | 0.0114 | 2.23E-04 | 2 |
| ENSG00000002933 | *TMEM176A* | IVW | -0.009 | 0.004 | 0.045 | 3 |
| ENSG00000002933 | *TMEM176A* | MR-Egger | -0.001 | 1.85E-05 | 0.022 | 3 |
| ENSG00000002933 | *TMEM176A* | MR-PRESSO | NA | NA | NA | NA |
| ENSG00000002933 | *TMEM176A* | LDA-MR-Egger | -0.004 | 0.001 | 0.159 | 3 |
| ENSG00000254030 | *IGLC5* | IVW | -0.040 | 0.016 | 0.010 | 1 |
| ENSG00000165507 | *C10orf10* | IVW | -0.071 | 0.030 | 0.018 | 1 |
| ENSG00000106565 | *TMEM176B* | IVW | -0.010 | 0.004 | 0.010 | 4 |
| ENSG00000106565 | *TMEM176B* | MR-Egger | -0.014 | 0.008 | 0.201 | 4 |
| ENSG00000106565 | *TMEM176B* | MR-PRESSO | NA | NA | NA | NA |
| ENSG00000106565 | *TMEM176B* | LDA-MR-Egger | -0.005 | 0.003 | 0.258 | 4 |

This table lists the results of inverse variance weighting (IVW), MR-Egger, LDA-MR-Egger and MR-PRESSO for the 18 genes that pass significance in the MR-link analysis that used BIOS eQTL cohorts. Each row contains the following information: the Ensembl ID (*ensg_id*), gene ID (*gene_id*), method for causal estimation (method), causal estimate (*estimated beta*), standard error of the causal estimate (*SE*), *p* value of the estimate (*p_val_estimated*) (2 sided Wald test for IVW and 2 sided T statistic for the other methods) and the number of IVs identified by GCTA-COJO (*IVs_identified*). Please note that fewer than 3 IVs were identified for 13 genes, which means that MR-EGGER and LDA-MR-Egger were unable to make an estimate for these genes. For the other 5 cases, MR-PRESSO identified too many outliers and therefore MR-PRESSO estimates are not available for any of the genes.

**Supplementary References**

1. Tibshriani, R. Regression Shrinkage and Selection via the Lasso. *J. R. Stat. Soc. Ser. B* **58**, 267–288 (1996).

2. Zou, H. & Hastie, T. Regularization and variable selection via the elastic net. *J. R. Stat. Soc. Ser. B Stat. Methodol.* **67**, 301–320 (2005).

3. Cule, E., Vineis, P. & De Iorio, M. Significance testing in ridge regression for genetic data. *BMC Bioinformatics* **12**, 372 (2011).

4. Salvatier, J., Wiecki, T. V. & Fonnesbeck, C. Probabilistic programming in Python using PyMC3. *PeerJ Comput. Sci.* **2016**, e55 (2016).

5. Marchini, J., Cardon, L. R., Phillips, M. S. & Donnelly, P. The effects of human population structure on large genetic association studies. *Nat. Genet.* **36**, 512–517 (2004).
