# Supplementary material for: Mendelian randomization while jointly modeling cis genetics identifies causal relationships between gene expression and lipids
Source: Nat Commun. 2020 Oct 1;11:4930. doi: 10.1038/s41467-020-18716-x (PMC7530717; doi:10.1038/s41467-020-18716-x)
Supplement: Supplementary file 3 — Reporting Summary [file 41467_2020_18716_MOESM3_ESM.pdf]

nature research

Corresponding author(s): Serena Sanna

Last updated by author(s): Aug 3, 2020

Reporting Summary

Nature Research wishes to improve the reproducibility of the work that we publish. This form provides structure for consistency and transparency in reporting. For further information on Nature Research policies, see Authors & Referees and the Editorial Policy Checklist .

Statistics

For all statistical analyses, confirm that the following items are present in the figure legend, table legend, main text, or Methods section.

n/a	Confirmed	
☐	☒	The exact sample size (*n*) for each experimental group/condition, given as a discrete number and unit of measurement
☒	☐	A statement on whether measurements were taken from distinct samples or whether the same sample was measured repeatedly
☐	☒	The statistical test(s) used AND whether they are one- or two-sided<br>*Only common tests should be described solely by name; describe more complex techniques in the Methods section.*
☐	☒	A description of all covariates tested
☐	☒	A description of any assumptions or corrections, such as tests of normality and adjustment for multiple comparisons
☐	☒	A full description of the statistical parameters including central tendency (e.g. means) or other basic estimates (e.g. regression coefficient) AND variation (e.g. standard deviation) or associated estimates of uncertainty (e.g. confidence intervals)
☐	☒	For null hypothesis testing, the test statistic (e.g. *F*, *t*, *r*) with confidence intervals, effect sizes, degrees of freedom and *P* value noted<br>*Give P values as exact values whenever suitable.*
☐	☒	For Bayesian analysis, information on the choice of priors and Markov chain Monte Carlo settings
☒	☐	For hierarchical and complex designs, identification of the appropriate level for tests and full reporting of outcomes
☐	☒	Estimates of effect sizes (e.g. Cohen's *d*, Pearson's *r*), indicating how they were calculated

Our web collection on statistics for biologists contains articles on many of the points above.

Software and code

Policy information about availability of computer code

Data collection	No new data was collected for this study, instead we used data collected by the BIOS and Lifelines cohorts as well as Summary statistics from the GTEx consortium and from the Sun et al. study. Therefore, no software was used for data collection.
Data analysis	Quality control of genetic data and genetic association analyses in BIOS and Lifelines cohorts were performed using PLINK 1.9. Genotype imputation was carried out using the Michigan Imputation server for the BIOS cohort , and locally using minimac version 2012.10.3 for Lifelines cohort.<br><br>We used RNAseq data from the BIOS cohort that was already QCed, aligned, and adjusted for covariates from a previous study. In this previous study, RNA-seq read alignment was performed using STAR (version 2.3.0e), and gene expression quantification was carried out using HTseq (version v0.6.1p1).<br><br>Genotypes were simulated using HAPGEN2 (version 2.2.0). For simulation of phenotypes and causal inference analyses with MR-link or other MR methods, custom code was used. As described under "Code availability statement", an implementation of the novel statistical method presented in this manuscript, MR-link, the methods to re-create the simulated data and instructions on usage can be found at https://github.com/adriaan-vd-graaf/genome_integration .<br><br>Selection of jointly independent variables and of instrumental variables was performed with GCTA-COJO (version 1.26.0). Fine mapping of eQTL results was carried out using FINEMAP v1.3.1.<br><br>Colocalization analyses were performed using the coloc package v4, using the version with git commit 6f3cbb1e5e90f07de772339d6e4af362140affc3

For manuscripts utilizing custom algorithms or software that are central to the research but not yet described in published literature, software must be made available to editors/reviewers. We strongly encourage code deposition in a community repository (e.g. GitHub). See the Nature Research guidelines for submitting code & software for further information.

Data

Policy information about availability of data

All manuscripts must include a data availability statement. This statement should provide the following information, where applicable:

- Accession codes, unique identifiers, or web links for publicly available datasets
- A list of figures that have associated raw data
- A description of any restrictions on data availability

Individual level data of Lifelines cohorts are available to all bona-fide researchers upon request to the Lifelines biobank (https://www.lifelines.nl/researcher).
Individual level data (genotypes and RNA-seq data) of the BIOS Consortium cohorts can be downloaded by researchers of Dutch Institutes only, or analyzed (but not downloaded) by any non-Dutch researcher in a Cloud environment (https://www.bbmri.nl/acquisition-use-analyze/bios).
GTEx summary statistics can be downloaded from the GTEx website (https://gtexportal.org/home/datasets). pQTLs summary statistics can be downloaded from GWAS Catalog (ftp.ebi.ac.uk/pub/databases/gwas/summary_statistics/SunBB_29875488). Simulated data can be recreated using the code at the link provided in Code availability statement.
Imputation with the Haplotype Reference Consortium dataset can be done at the following link: (https://imputationserver.sph.umich.edu/index.html#!)

Raw data used to draw Figure 3 can be found in Supplementary Data 3

Field-specific reporting

Please select the one below that is the best fit for your research. If you are not sure, read the appropriate sections before making your selection.

☒ Life sciences ☐ Behavioural & social sciences ☐ Ecological, evolutionary & environmental sciences

For a reference copy of the document with all sections, see nature.com/documents/nr-reporting-summary-flat.pdf

Life sciences study design

All studies must disclose on these points even when the disclosure is negative.

Sample size	For simulations, we simulated the data sets with a sample size similar to that of the BIOS consortium cohorts, the dataset used as real data set in this study. For the BIOS and Lifelines cohorts, sample size was not predetermined; we used all available samples with genetic and phenotypic (or transcriptomic) information
Data exclusions	For the BIOS cohorts, we accessed genetic data and excluded genetically related individuals based on the genetic relationship matrix (GRM) (only one sample per pair) and ethnic outliers based on principal component analyses. For these exclusions, we used the following standard criteria: GRM value > 0.1 for relatedness, and samples with values >3 standard deviations of the mean of principal component 1 or principal component 2.<br>Genotyped variants were discarded with the following criteria: we discarded non-biallelic SNPs and indels, variants with minor allele frequency (MAF)< 0.01 or Hardy-Weinberg equilibrium (HWE) p value <10^-6 and an imputation quality RSQR <= 0.8<br>We also removed samples based on RNA-seq quality data. In particular, we discarded samples with <85% of RNA-seq reads mapping to exons or with <80% mapped RNA-seq reads, which are standard criteria used in RNAseq studies.<br>For the Lifelines cohort, we used preQCed genotyping data for imputation. In brief, variants were kept if they had a minor allele frequency > 0.001, had less than 5% missingness and had a HWE p value > 0.0001. After imputation, we excluded low quality imputed variants using the standard imputation quality RSQR < 0.3 and HWE p <10^-6. We did not exclude any individual based on genotyping data, as this quality control step was already performed. We instead excluded individuals with total triglycerides levels <= 4.52 mmol/Liter, following recommendations for a correct definition of low density lipoprotein cholesterol using the Friedewald formula.<br>When performing MR analyses on the Lifelines data together with the eQTLs derived from BIOS, we restricted analyses to variants that were present in both Lifelines and BIOS cohorts, because variants present in only one dataset cannot be incorporated in the statistical model.<br>Likewise, when performing analyses using the eQTLs derived from GTEX or the pQTLs from the protein QTL summary statistics, we restricted analyses to variants present jointly in these data sets and in the Lifelines cohort.
Replication	We did not look for replication of our estimates. However, we showed that results obtained using the large BIOS blood eQTLs and the small GTEX blood eQTL were concordant (see Supplementary Figure 4B). Furthermore, our findings on real data sets are well supported by the extended literature on the role of these genes in the phenotype being analyzed.
Randomization	This is not relevant to our study, this is an observational study
Blinding	This is not relevant to our study, this is an observational study

Reporting for specific materials, systems and methods

We require information from authors about some types of materials, experimental systems and methods used in many studies. Here, indicate whether each material, system or method listed is relevant to your study. If you are not sure if a list item applies to your research, read the appropriate section before selecting a response.

Materials & experimental systems

n/a	Involved in the study
☒	☐ Antibodies
☒	☐ Eukaryotic cell lines
☒	☐ Palaeontology
☒	☐ Animals and other organisms
☐	☒ Human research participants
☒	☐ Clinical data

Methods

n/a	Involved in the study
☒	☐ ChIP-seq
☒	☐ Flow cytometry
☒	☐ MRI-based neuroimaging

Human research participants

Policy information about studies involving human research participants

Population characteristics

This study used two data sets: BIOS Consortium cohorts and Lifelines cohort.

The BIOS consortium is a data set of 3,746 Dutch individuals from six different cohorts: Lifelines DEEP, Prospective ALS Study Netherlands, Leiden Longevity Study, Netherlands Twin Registry, The Cohort on Diabetes and Atherosclerosis Maastricht and the Rotterdam Study. The average age was 52.8 years (+/-16 Stand.Dev) and 57% were female.

The Lifelines cohort is a subset of 13,436 samples from the LifeLines biobank, a population-based study from North Netherlands. In this subset, 58.8% were female and the average age was 48.7 years (+/-11.5 Stand.Dev.)

Recruitment

Volunteers for both BIOS and Lifelines cohorts were recruited independently of this study.
Lifelines volunteers were selected from the general population and recruited on voluntary participation after an invitation letter to the general practitioners. We thus envisage that individuals with severe diseases and unable to visit the recruitment center are under-represented in this cohort. Given the large sample size and the phenotypic end point analyzed, we consider unlikely that this ascertainment bias could affect the reported results.
Volunteers from the BIOS cohorts were also mostly collected from the general population. The six cohorts are: 1) Lifelines DEEP cohort, which is a random subset of Lifelines, 2) the Prospective ALS Study Netherlands, which is a prospective population study aimed to study epidemiology of ALS 3) the Leiden Longevity Study, which is a prospective study of long-living individuals and probands 4) The Netherlands Twin Registry, which is a prospective population study of adolescents and adults 5) the Rotterdam Study which is a prospective population study of adults >45year of age and 6) The Cohort on Diabetes and Atherosclerosis Maastricht (CODAM), which is a prospective, observational study on, among others, the natural progression of IR and glucose tolerance.
None of these cohorts specifically recruited samples with hematological or immune-related diseases, and therefore is unlikely that any ascertainment bias will affect the analyses of transcriptomic data in blood.
Overall, we conclude that recruitment scheme in the data sets used in this study are unlikely to affect the results reported.

Ethics oversight

We did not collect new data for this study. We used available data from existing cohorts which were approved by their respective ethical committees. In particular:
The Lifelines study was approved by the medical ethics committee of the University Medical Center Groningen and conducted in accordance with Helsinki Declaration Guidelines
All cohorts from the BIOS consortium were approved by their ethical committees, as follow: the LLDEEP was approved by the medical ethics committee of the University Medical Center Groningen; the Prospective ALS Study Netherlands was conducted with the approval of the institutional review board of the University Medical Centre Utrecht; the Leiden Longevity Study was approved by the Medical Ethical Committee of the Leiden University Medical Center; the Netherlands Twin Registry was approved by Central Ethics Committee on Research Involving Human Subjects of the VU University Medical Center, Amsterdam, an Institutional Review Board certified by the US Office of Human Research Protections (IRB number IRB-2991 under Federal-wide Assurance-3703; IRB/institute codes, NTR 03-180) ; the Rotterdam Study was approved by the institutional review board (Medical Ethics Committee) of the Erasmus Medical Center and by the review board of The Netherlands Ministry of Health, Welfare and Sports; the CODAM study was approved by the medical ethics committee of Maastricht University.
Informed consent was obtained from all the participants of Lifelines and BIOS cohorts.

Note that full information on the approval of the study protocol must also be provided in the manuscript.
