# Supplementary material for: Mendelian randomization while jointly modeling cis genetics identifies causal relationships between gene expression and lipids
Source: Nat Commun. 2020 Oct 1;11:4930. doi: 10.1038/s41467-020-18716-x (PMC7530717; doi:10.1038/s41467-020-18716-x)
Supplement: Supplementary file 4 — Description of Additional Supplementary Files [file 41467_2020_18716_MOESM4_ESM.pdf]

Description of Additional Supplementary Files

Supplementary Data 1
Overlap between genes and variants identified by FINEMAP
This table reports how often eQTL variants of a gene are in LD with eQTL variants of another gene, based on variant configurations identified by FINEMAP (Methods) at different posterior inclusion probabilities (PIP) or at the top configuration (top_config) (column named PIP_levels). We distinguish between `gene_overlap` and `full_gene_overlap` (column named type_of_test). `gene_overlap` describes if at least one variant in the configuration(s) overlaps with at least one variant in a configuration of another gene, while `full_gene_overlap` describes how often all the variants in the selected configuration(s) for all genes are overlapping or in LD according to a given r^2 LD threshold, threshold indicated in column LD_threshold_r_2. The number of genes with overlap at that specific threshold, configuration and test is shown in column number_within_LD_range and the percentage over the total genes (column percentage_of_total).

Supplementary Data 2
False positive rates and power of different MR methods and IV selection when no pleiotropy is simulated
Each row describes a simulation scenario and the corresponding detection rate using different approaches for IVs selection and different MR methods in a non-pleiotropic scenario ($b_U = 0$) (Methods). Simulation scenarios were varied by the (1) number of simulated causal SNPs (column n_causal), (2) causal effect of the known exposure (column exposure_1_causal) and (3) MR estimation method (column estimation_method). The approach for IV selection (column selection_method) varied between GCTA COJO (COJO) and p value clumping (clumped). Results are indicated in the last three columns, specifically: the median number of IVs identified by the selection method (column median_ivs_identified), how often (out of 1,500 simulations) an estimation was made (column number_of_simulations) and how often a method identifies a significant effect at alpha = 0.05 (column detection_rate). Note that detection rate is equivalent to false positive rate when exposure_1_causal = 0 and equivalent to detection power otherwise. See the Supplementary Notes 1 for an explanation of the MR methods MR-link OLS, MR-link ridge and MR-link ridge p calibrated. Significance for the other MR methods is determined using a two sided Wald test in the case of IVW, and a two sided T test in the case of MR-Egger, MR-PRESSO and LDA-MR-Egger.

Supplementary Data 3
False positive rate and power of different MR methods when pleiotropy through linkage disequilibrium is simulated
Each row describes a simulation scenario and the corresponding detection rate for different MR methods in a pleiotropic scenario ($b_U = 0.4$) (Methods). Simulation scenarios were varied by the (1) the number of simulated causal SNPs (column n_causal), (2) causal effect of the known exposure (column exposure_1_causal) and (3) MR estimation method (column estimation_method). Other columns indicate: the median number of IVs identified by GCTA-COJO (column median_ivs_identified), how often (out of 1,500 simulations) an estimate was made (column number_of_simulations) and how often a method identifies a significant effect at alpha = 0.05 (column detection_rate). Note that detection rate is equivalent to false

positive rate when exposure_1_causal=0 and equivalent to detection power otherwise. See the Supplementary Notes 1 for an explanation of the MR methods MR-link OLS, MR-link ridge and MR-link ridge p calibrated. Significance for the other MR methods is determined using a two sided Wald test in the case of IVW, and a two sided T test in the case of MR-Egger, MR-PRESSO and LDA-MR-Egger.

Supplementary Data 4
False positive rate and power of different MR methods when pleiotropy through overlap is simulated
Each row describes a simulation scenario and the corresponding detection rate for different MR methods in a pleiotropic scenario ($b_U = 0.4$) with 10 simulated causal SNPs and an increasing number of overlapping pleiotropic SNPs (Methods). The first columns indicate (1) the causal effect of the known exposure (column exposure_1_causal), (2) the MR estimation method (column estimation_method) and (3) the number of causal variants that overlap between the known and the pleiotropic exposure (column overlapping_causal). The other columns indicate: the median number of IVs identified by the GCTA-COJO (column median_ivs_identified), how often (out of 1,500 simulations) an estimate was made (column number_of_simulations) and how often a method identifies a significant effect at alpha = 0.05 (column detection_rate). Note that detection rate is equivalent to false positive rate when exposure_1_causal = 0 and equivalent to detection power otherwise. See the Supplementary Notes 1 for an explanation of the MR methods MR-link OLS, MR-link ridge and MR-link ridge p calibrated. Significance for the other MR methods is determined using a two sided Wald test in the case of IVW, and a two sided T test in the case of MR-Egger, MR-PRESSO and LDA-MR-Egger.

Supplementary Data 5
Discriminative ability of coloc methods and MR-link in simulations
Each row describes the discriminative ability in terms of area under the receiver operator characteristic curve (AUC) comparing the scenario with no causal effect $b_E = 0$ with a scenario where there is a causal effect $b_E \neq 0$ (column exposure_1_causal). Simulations were varied in the number of simulated causal variants (column n_causal), presence ($b_U = 0.4$) or absence ($b_U = 0$) of pleiotropy (column exposure_2_causal) and the level of pleiotropy through overlap (column overlapping_causal), where zero means full pleiotropy through linkage disequilibrium (LD) and 10 means full pleiotropy through overlap between observed and unobserved exposure. MR-link and IVW were compared to three coloc methods for discriminative ability (column estimation_method) in terms of the AUC (column AUC). See Methods for specifics of the simulations and how the AUC was calculated.

Supplementary Data 6
False positive rate and power for coloc methods in simulations
Each row describes a simulation scenario and the corresponding detection rate for three different coloc methods in all simulation scenarios (Methods). The first 5 columns indicate the simulation parameters: (1) the number of simulated causal SNPs (columns n_causal) (2) the causal effect of the known exposure (column exposure_1_causal), (3) the presence of a pleiotropic effect (column exposure_2_causal), (4) the coloc estimation method (column estimation_method) and (5) the number of causal variants that overlap between the known and the

pleiotropic exposure (column overlapping_causal). The other columns indicate results: how often (out of 1,500 simulations) an estimate was made (column number_of_simulations) and how often the coloc method identifies sharing of causal variants at coloc PP4 > 0.9 (the maximum is taken if there is more than 1 coloc estimate) (column detection_rate). Note that detection rate is equivalent to false positive rate when exposure_1_causal = 0 and equivalent to power otherwise.

Supplementary Data 7
False positive rates and power of MR-link when using a different p value calibration procedure
Each row describes a simulation scenario and the corresponding detection rate for MR-link when p values are calibrated based on a heterogeneous distribution of p values (The scenarios without pleiotropic effect and pleiotropy through linkage disequilibrium combined) (Supplementary Notes 1). The first columns indicate the simulation parameters: (1) the number of causal variants simulated (column) (2) the causal effect of the known exposure (column exposure_1_causal), (3) the pleiotropic effect (column exposure_2_causal) The other columns indicate results of the simulations: the median number of IVs identified by the GCTA-COJO (column median_ivs_identified), how often (out of 1,500 simulations) an estimate was made (column number_of_simulations) and how often a method identifies a significant effect at alpha = 0.05 (column detection_rate). Note that detection rate is equivalent to false positive rate when exposure_1_causal = 0 and equivalent to detection power otherwise. Significance for MR-link was determined based on a calibrated p value as described in Supplementary Notes 1.
