# Supplementary material for: Mendelian randomization while jointly modeling cis genetics identifies causal relationships between gene expression and lipids
Source: Nat Commun. 2020 Oct 1;11:4930. doi: 10.1038/s41467-020-18716-x (PMC7530717; doi:10.1038/s41467-020-18716-x)
Supplement: Supplementary file 2 — Peer Review [file 41467_2020_18716_MOESM2_ESM.pdf]

Reviewers' comments:

Reviewer #1 (Remarks to the Author):

This is an interesting and well written paper. I would only ask the Authors to give a clearer justification of the Mendelian Randomisation (MR-link) method they propose, as I explain later in this document. That said, I find this a very good paper, which brings together substantive knowledge and methodological insight. I believe that, conditional on the Authors providing a sound reply to my points, this paper is well worth publication on Nature Communications

I have the following points:

POINT 1. I believe that the validity assumptions of the proposed method, MR-link, should be made more explicit in the text. As far as I understand, symbol X defines a set of genetic variants scattered along the cis-region that surrounds a transcript of interest. The Authors partition this set into two mutually exclusive subsets, sE and sU. Subset sE includes observed variants chosen to act as IVs, which the Authors assume NOT to influence the outcome other than through the transcript of interest and through their being in LD with variants in sU. Under this assumption, the Authors correctly conclude that violations of exclusion-restriction can be satisfactory dealt with by allowing the model to condition on sU, as done in Equation (4) of their model.
QUESTION 1.1: Could the Authors elaborate on the way they select the two subsets, sE and sU?
QUESTION 1.2: The Authors make the assumption that, conditional on sU, variants in sE exert no pleiotropic effect on the outcome. This assumption cannot be tested in a direct, statistical, way. This leads to the following question. How realistic is that assumption, and how robust is MR-link to violations of it?

POINT 2. Imagine a GWAS locus contains genes G1 and G2, and that G1 causally influences G2, resulting in a correlation between the two genes. In such a situation, any IV for G1 will be likely to be marginally associated with both G1 and G2. How would the Authors, in this situation, proceed to define a set sE of IVs for G1?

POINT 3. Line 463 reads "define the exposure and the unobserved (pleiotropic) exposure as in equation (1)". The concept of "unobserved exposure" remains vague for me. Could the Authors express it in greater detail? I would strongly suggest that the Authors do so with the aid of a diagram.

POINT 4. In their review of existing MR methods, eg. at page 3 of the manuscript, the Authors present an incomplete picture of the existing MR methodological panorama. For example, the method proposed by Berzuini and colleagues [C Berzuini, H Guo, S Burgess and L Bernardinelli: A Bayesian approach to Mendelian randomization with multiple pleiotropic variants. Biostatistics (2018) pp. 1–16, doi:10.1093/biostatistics/kxy027], deals with correlated instruments (for greater power), high uncertainty about eQTL associations, and unobserved pleiotropy, with weaker assumptions than MR-link.

MINOR POINT 1. At line 422, variable C is defined in an ambiguous way. I guess C should be defined as an n-vector of independent scalar draws from N(0,0.5). Each component of C acts as an individual-specific confounder. I think it would be worth while clarifying that the symbol C that appears in Equations (1-3) refers to the same quantity. I also believe the Authors should be explicit about the probability distribution of Co, rather than simply describing this variable as "some confounder".

MINOR POINT 2. At line 463 there is a missing parenthesis.

Carlo Berzuini
Research Professor in Biostatistics

Centre for Biostatistics
University of Manchester
Manchester
United Kingdom
https://orcid.org/0000-0001-6056-0489

Reviewer #2 (Remarks to the Author):

• Overview : The authors have created a novel method of Mendelian Randomization (MR) called MR-link which aims to address to the biggest pitfalls of MR. This novel method includes multiple aspects that that make significant improvements over standard MR approaches. While the authors demonstrate that their method outperforms some of the standard MR approaches, the authors fail to compare their method to other published non-MR methods which also aim to identify disease casual genes through xQTL data.
• The authors claim to account for pleiotropy. While they have tried to address some pleiotropy, but it is by no means a complete solution for all pleiotropy. They need to be more specific in the type of pleiotropy they are accounting for. For example, they don't address genetic pleiotropy across tissues and phenotypes.
• While this manuscript compares their MR-link to current MR methods, they fail to compare it to other methods that attempt to identify disease causal genes through xQTL data that also accounts for LD. Please compare the MR-link method to at least one type of colocalization method (e.g. coloc: https://github.com/chr1swallace/coloc) and at least one other method (e.g. MetaXcan: https://github.com/hakyimlab/MetaXcan). Please include an 1 example where your method out performs these methods, and 1 example where you method fails compared to these other methods.
• The argument in section "Genetic regulation of gene expression is often shared between genes through linkage disequilibrium" is very weak. Please use the current standard in the field of statistical finemapping and colocalization to identify shared genetic signal.
• Kudos for identifying independent signals for IV selection through GCAT-COJO. This is a much improved compared to the more standard MR LD clumping method.
• My understanding is that the authors have used the conditional Betas for the exposure in their analysis (p.27,L.452). Why would you only include the conditional Betas for the exposure and not the outcome?
• I don't find Figure 2 helpful for explaining "Causality with pleiotropy through overlap" vs "causality with pleiotropy through LD". Please try improving the figure. It may help to show "LocusZoom" like plots? This point is quite important to the paper and deserves a full figure to clearly illustrate your definitions.
• Pleiotropy can refer to the effect a genetics signal has through multiple genes or across multiple outcomes. Here, it seems like the authors have defined "unknown pleiotropic exposure" as the effect of other genes in a locus on the outcome (Figure2B; p.27,L461; p.28,L.472). After looking at the methods, I believe each model represents just 1 gene eQTL in a pairwise analysis with the 1 GWAS and is modeling the effect of the other genes as "unknown pleiotropic exposure". However, these other genes *have* data on their effect on the outcome. It seems like the effect on the outcome of the other genes in the locus effect are being estimated instead of using the actual xQTL data that is in-hand (e.g. p.28,L472). Please confirm this isn't a misunderstanding and improve the text to address the issue.
• P.26,L.468 – Should there be an additional error term for the measurement error of the exposure?

We are grateful to the reviewers for carefully reading our manuscript and for their positive comments and useful suggestions. We have addressed all the points raised in their comments, and we provide point-by-point responses to their questions below. The changes in the manuscript are marked in red. In addition, we have made some changes to the original manuscript that were not requested by the reviewers. In the interest of clarity, we have described these changes after our point-by-point responses to the reviewers. Please note, pages and line numbers always refer to the manuscript version in which changes are shown.

Reviewer #1:
This is an interesting and well written paper. I would only ask the Authors to give a clearer justification of the Mendelian Randomisation (MR-link) method they propose, as I explain later in this document. That said, I find this a very good paper, which brings together substantive knowledge and methodological insight. I believe that, conditional on the Authors providing a sound reply to my points, this paper is well worth publication on Nature Communications
We thank the reviewer for these kind words. We have provided a point-by-point response to the specific questions raised below.

I have the following points:

R1Q1.1. I believe that the validity assumptions of the proposed method, MR-link, should be made more explicit in the text. As far as I understand, symbol X defines a set of genetic variants scattered along the cis-region that surrounds a transcript of interest. The Authors partition this set into two mutually exclusive subsets, sE and sU. Subset sE includes observed variants chosen to act as IVs, which the Authors assume NOT to influence the outcome other than through the transcript of interest and through their being in LD with variants in sU. Under this assumption, the Authors correctly conclude that violations of exclusion-restriction can be satisfactory dealt with by allowing the model to condition on sU, as done in Equation (4) of their model.
Could the Authors elaborate on the way they select the two subsets, sE and sU?

R1A1.1. *We apologize for not making this sufficiently clear. We have now improved the Methods section.*

*In the section “**Simulation of phenotypes”** that starts on page 19, we have incorporated the description of the procedure for the selection of s_E and s_U subsets. Of note, these two subsets are not necessarily mutually exclusive. Specifically, in the scenario of partial or total overlap between the exposure and unobserved pleiotropic exposure, some of the variants in the two subsets are shared. We have amended the paragraph “**Simulation of phenotypes**”, page 19, lines 417-429, which now reads:*

*“We simulated quantitative phenotypes representing the exposures by randomly selecting SNPs from the simulated genetic region, and subsequently assigning these an effect. Causal SNPs were selected to represent both pleiotropy through LD (**Figure 2B**) and pleiotropy through overlap (**Figure 2C**). For the scenario of pleiotropy through LD (**Figure 2B**), one to ten causal SNPs (subset $\boldsymbol{s}_E$) for the exposure were randomly selected from the entire simulated genetic region, and the same number of causal SNPs (subset $\boldsymbol{s}_U$) for the unobserved (pleiotropic) exposure was randomly selected from all SNPs in moderate LD (0.25 < r^2 < 0.95) with SNPs in $\boldsymbol{s}_E$.”*
*We have also updated the IV and tag SNP selection procedure in the section titled “**MR-link**”. We have updated the explanation of how the IVs and variants that are tagged by these IVs are chosen in MR-link on pages 22 and 23, lines 497-513. This section now reads:*

“MR-link uses the following procedure to estimate causal effects:

(1) *A selection $\hat{\boldsymbol{s}}_E$ of IVs for the exposure and conditional effect sizes $\hat{\boldsymbol{\beta}}_E$ for these IVs are determined using the GCTA-COJO method25. A vector of effect sizes $\hat{\boldsymbol{\beta}}_E$ for all SNPs in the region is thus defined as:* $\hat{\boldsymbol{\beta}}_{E,j} = \begin{cases} \neq 0 & if j \in \hat{\boldsymbol{s}}_E \\ 0 & otherwise \end{cases}, \forall j \in \{1, \dots, m\}$.

(2) *All SNPs in LD $0.1 < r^2 < 0.99$ with the exposure IVs are potential tag-SNPs. These variants are iteratively pruned for high LD so that tag-SNPs, $\boldsymbol{s}_T$, are always $r^2 < 0.95$ with each other in order to reduce collinearity and computation time.*

(3) *The following equation is solved for b_E using ridge regression:*

$$y_O = \begin{pmatrix} \vdots & \vdots \\ \frac{X\hat{\boldsymbol{\beta}}_E}{m_E} & \frac{X_T}{\sqrt{m_T}} \\ \vdots & \vdots \end{pmatrix} \begin{pmatrix} b_E \\ \vdots \\ \boldsymbol{\beta}_U b_U \\ \vdots \end{pmatrix} + \epsilon, \quad (5)$$

where $\boldsymbol{X}_T$ is the genotype matrix of the outcome containing only tagging variants as defined in step (2), m_T is the number of tagging variants and is used to normalize for the number of tags in the region, and m_E represents the number of IVs selected by the selection method and is a parameter used to remove the dependency of the model on the number of IVs. The resulting coefficient vector contains the causal effect of interest b_E, and the vector $\boldsymbol{\beta}_U b_U$ of length m_T is a nuisance parameter that captures pleiotropic effects.

Because individual-level data of the outcome is modeled by MR-link, MR-link does not use any summary statistics of the outcome."

R1Q1.2: The Authors make the assumption that, conditional on sU, variants in sE exert no pleiotropic effect on the outcome. This assumption cannot be tested in a direct, statistical, way. This leads to the following question. How realistic is that assumption, and how robust is MR-link to violations of it?

R1A1.2 *We thank the reviewer for pointing out the need to elaborate on the main assumption of MR-link. MR-link assumes that conditional on s_U, variants in s_E exert no pleiotropic effect on the outcome. This is equivalent to the assumption that pleiotropy comes from variants in LD with the IVs (pleiotropy through LD) and that pleiotropy through overlap is absent or very limited (only a subset of s_E variants are in s_U).*

To understand how realistic this assumption is, we initially looked at gene expression in the BIOS cohort. In our original manuscript, we showed that in the BIOS cohort violations of this assumption are restricted to a limited set of genes. We also observed that the IVs of gene expression changes are rarely fully overlapping between genes. This analysis was carried out by looking at variants selected by GCTA-COJO. In the new manuscript, we have investigated this further using fine-mapping analyses. For all genes in our eQTL dataset, we evaluated the degree of sharing of likely causal variants identified using the FINEMAP method. The results of this analysis, which was also suggested by reviewer #2 (R2Q3), reiterate that sources of pleiotropy from gene expression mostly come from variants that are in LD with IVs, and are less likely to come from exactly the same variants that were chosen as IVs themselves. Therefore, both analyses suggest that the main assumption of MR-link is realistic in the case of gene expression as an exposure.

*In our simulations, we included a scenario to evaluate the robustness of MR-link to violations of the main assumption. Specifically, this is the simulation scenario of full overlap <Page 8 and 9, lines 180-188>, where all causal variants are overlapping ($s_E = s_U$). Here, the false positive rate of MR-link increases compared to other tested methods, but still retains the best power (**Supplementary Table 4**) and shows superior discriminative ability compared to coloc (**Supplementary Figure 2L**).*

*We have now improved these points of discussion in the manuscript. First, we have stated the main assumption of MR-link in the text by adding the following paragraph in the section "**MR-link outperforms other MR methods in discriminative ability**" on pages 6 and 7, lines 138 to 146*

“Strictly speaking, MR-link corrects for pleiotropy under the assumption that pleiotropy can be better explained by variants in LD with the IV (pleiotropy through LD) (Figure 2B) and that pleiotropy through overlap is absent (Figure 2C). In case of a single IV, this assumption needs to be accounted for, but when multiple IVs are available, this assumption can be relaxed somewhat. Differences in effect -sizes between IVs can be used to distinguish the causal effect of interest from a pleiotropic effect, in the same way that multivariable MR corrects for pleiotropy22. Of note, MR-link does not require the source of pleiotropy to be specified in the model; MR-link can account for pleiotropic effects arising from, for instance, gene expression in other tissues or from other molecular layers or phenotypes.”

*Second, we have added the results of the FINEMAP analysis next to the analyses performed with GTCA-COJO in the paragraph “**eQTL variants between different genes are often in LD**”, on page 6, lines 115-124. The additional paragraph reads as follows:*

*“To strengthen our inferences on the genetic regulation of gene expression in cis, we performed statistical fine-mapping using FINEMAP v1.3.1[26] on 13,276 genes (**Methods**) (**Supplementary Methods**). Only 373 (2.8%) genes have full eQTL overlap (all variants in the top configuration of a gene are identical or in high LD (r^2 > 0.99)), while 33.2% of the genes have at least one variant in r^2 > 0.5 LD with a variant in the top configuration of another gene. These percentages are higher for configurations with larger posterior inclusion probabilities (**Methods**) (**Supplementary Table 1**), but overall the results are similar to our observations from the GCTA-COJO analysis, i.e. the genetics of gene expression in whole blood is mostly regulated by variants that do not overlap but are in moderate LD with variants associated with gene expression changes of another gene. Based on these results, it seems likely that pleiotropy through LD is more common than pleiotropy through overlap in gene expression traits.“*

*We have also elaborated on violations of this assumption in the **Discussion** section on pages 13 and 14, lines 299-306.*

“One of the MR-link assumptions is that the IVs affect the outcome only through the exposure, conditional on the unmeasured pleiotropic effect. This assumption is violated when the IVs of the exposure and of the pleiotropic effect are fully overlapping. This assumption must not be violated when a single IV is available, but can be relaxed when multiple IVs are used in the model, as the relative effects of the IVs help to discriminate between a true causal effect and a pleiotropic effect, similar to multivariable Mendelian randomization methods[22]. In the case of multiple IVs that are fully overlapping, we have shown that MR-link has an increased FPR, yet still maintains higher power compared to other MR-methods and superior discriminative ability compared to coloc.“

R1Q2. Imagine a GWAS locus contains genes G1 and G2, and that G1 causally influences G2, resulting in a correlation between the two genes. In such a situation, any IV for G1 will be likely to be marginally associated with both G1 and G2. How would the Authors, in this situation, proceed to define a set sE of IVs for G1?

R1A2. *If we understand Reviewer #1 correctly, we are in a case where G1 causally influences G2 (G1 → G2) and also an outcome O (G1 → O). If G2 is also causal to the outcome (G1 → G2 → O), there will be no horizontal pleiotropic effect when looking at a causal effect between G1 and the hypothetical outcome O (G1 → O), and thus no violation of assumptions occurs. The selection of IVs for both G1 and G2 will follow the standard procedure. If all IVs (or a large fraction of IVs) for G1 are also IVs for G2, we are in a situation comparable to the scenario of pleiotropy through overlap, for which we expect a reduction in performance proportional to the degree of overlap.*

We recognize that this particular scenario will violate the main assumption of MR-link, as well as of other MR methods such as Multivariate MR, but as detailed in R1A1, our fine-mapping analyses show that, in the context of eQTLs, this is likely to be an issue in only a fraction of loci.

R1Q3. Line 463 reads "define the exposure and the unobserved (pleiotropic) exposure as in equation (1)". The concept of "unobserved exposure" remains vague for me. Could the Authors express it in greater detail? I would strongly suggest that the Authors do so with the aid of a diagram.

R1A3 *We thank the reviewer for pointing this out. The concept of "unobserved exposure" refers to any source of pleiotropy that originates from an unmeasured phenotype, which is in contrast to multivariable MR methods that require QTL variants of all pleiotropic phenotypes to be included in the model.*

Our model doesn't require the pleiotropic effect to be measured and included in the model. Unobserved pleiotropy is accounted for in MR-link as a nuisance variable, and it refers to the pleiotropic effect of any trait. We have now included the following text describing the unobserved pleiotropy in the manuscript (page 4, lines 70-72):

"Likewise, it is not always possible to measure all sources of pleiotropy because pleiotropy it could come from expression of a gene in a different tissue or even from other unmeasured unobserved molecular marks or phenotypes."

In addition, we now separated the original Figure 2 into two figures. The new Figure 2 uses graphical representations of a locus and corresponding diagrams to explain the sources of pleiotropy in a cis locus, as well as unobserved pleiotropy. The new Figure 3 shows the results of multiple MR-methods in simulations and is identical to the lower panels of the original Figure 2.

R1Q4. In their review of existing MR methods, eg. at page 3 of the manuscript, the Authors present an incomplete picture of the existing MR methodological panorama. For example, the method proposed by Berzuini and colleagues [C Berzuini, H Guo, S Burgess and L Bernardinelli: A Bayesian approach to Mendelian randomization with multiple pleiotropic variants. Biostatistics (2018) pp. 1–16, doi:10.1093/biostatistics/kxy027], deals with correlated instruments (for greater power), high uncertainty about eQTL associations, and unobserved pleiotropy, with weaker assumptions than MR-link.

R1A4 *We thank the reviewer for suggesting this Bayesian method. In our manuscript and table, we focused on 2-sample MR methods, and thus have not included this MR approach, which is based on a one-sample setting. Nevertheless, it addresses similar challenges and we have now added a reference to the paper in the manuscript (line 64 and line 68), with the added explanation that it allows for correct inference of causal effects under pleiotropy using a one-sample MR approach.*

R1Q5. At line 422, variable C is defined in an ambiguous way. I guess C should be defined as an n-vector of independent scalar draws from N(0,0.5). Each component of C acts as an individual-specific confounder. I think it would be worth while clarifying that the symbol C that appears in Equations (1-3) refers to the same quantity. I also believe the Authors should be explicit about the probability distribution of Co, rather than simply describing this variable as "some confounder".

R1A5 *We thank the reviewer for pointing out this inconsistency. We have adapted the manuscript to fix this issue at line 422 (now lines 436-437). The sentence now reads "and $C \sim N(0,0.5)^n$ a n-vector of independent scalar draws from N(0,0.5), representing cohort specific confounder per individual".*

Additionally, we indicated at lines 453-454 that the symbol C is drawn in a cohort-specific manner. Equations (1-3) refer to the same quantity.

R1Q6. At line 463 there is a missing parenthesis.

A1Q6 *We apologize for this typo. We have added the missing parenthesis and removed the 0 character.*

Reviewer #2:

Overview : The authors have created a novel method of Mendelian Randomization (MR) called MR-link which aims to address to the biggest pitfalls of MR. This novel method includes multiple aspects that make significant improvements over standard MR approaches. While the authors demonstrate that their method outperforms some of the standard MR approaches, the authors fail to compare their method to other published non-MR methods which also aim to identify disease casual genes through xQTL data.

We thank the reviewer for the positive comments about our work and for the constructive criticisms. In our response below, we describe in detail the additional analyses that we have now included to compare our method with other non-MR methods.

R2Q1: The authors claim to account for pleiotropy. While they have tried to address some pleiotropy, but it is by no means a complete solution for all pleiotropy. They need to be more specific in the type of pleiotropy they are accounting for. For example, they don't address genetic pleiotropy across tissues and phenotypes.

R2A1: *We thank the reviewer for pointing out the need to clarify the assumptions of MR-link and the pleiotropic scenarios that it can address. This issue was also raised by Reviewer #1. As detailed in our answers to R1Q3, our model doesn't require the pleiotropic effect to be measured and included in the model, because we use the genetic variants surrounding the IVs to estimate the pleiotropic effect. Therefore, we model any source of pleiotropy referring to any quantitative traits (including gene expression in different tissues or other phenotypes such as protein levels). We have now clarified this in the manuscript (lines 70-72) and included a new figure (Figure 2) that explains the sources of bias in an MR analysis.*

R2Q2: While this manuscript compares their MR-link to current MR methods, they fail to compare it to other methods that attempt to identify disease causal genes through xQTL data that also accounts for LD. Please compare the MR-link method to at least one type of colocalization method (e.g. coloc: https://github.com/chr1swallace/coloc) and at least one other method (e.g. MetaXcan: https://github.com/hakyimlab/MetaXcan). Please include an 1 example where your method out performs these methods, and 1 example where you method fails compared to these other methods.

R2A2: *We agree with that reviewer that other non-MR methods are commonly used to identify causal genes through xQTL data. While such methods are very easy to implement, these methods cannot always distinguish causal from pleiotropic effects [1]. We therefore expect that they will not outperform causal inference methods such as MR. To demonstrate this, we have now implemented the 'coloc' method [2] in our simulations. We have compared MR-link to coloc comprehensively in our simulations (page 9, lines 189-193) and found that MR-link has superior discriminative ability for all simulation scenarios for which both methods have distinctive discriminative ability (area under the receiver operator characteristic curve (AUC) > 0.55) (**Supplementary Figure 2** and **Supplementary Table 5**). In the case of pleiotropy through overlap and small causal effects, there is limited power for both approaches and their relative performance*

is similar, although MR-link still performs slightly better. While we can conclude that MR-link outperforms coloc, we acknowledge that it has the drawback that it needs individual-level data of the outcome, whereas coloc only requires summary level data. Therefore, coloc still remains a more flexible method for prioritizing genes.

[1] Wainberg, M., Sinnott-Armstrong, N., Mancuso, N. et al. Opportunities and challenges for transcriptome-wide association studies. Nat Genet 51, 592–599 (2019) doi:10.1038/s41588-019-0385-z
[2] Bayesian Test for Colocalisation between Pairs of Genetic Association Studies Using Summary Statistics Giambartolomei C, Vukcevic D, Schadt EE, Franke L, Hingorani AD, et al. (2014) Bayesian Test for Colocalisation between Pairs of Genetic Association Studies Using Summary Statistics. PLOS Genetics 10(5): e1004383. https://doi.org/10.1371/journal.pgen.1004383

R2Q3: The argument in section "Genetic regulation of gene expression is often shared between genes through linkage disequilibrium" is very weak. Please use the current standard in the field of statistical finemapping and colocalization to identify shared genetic signal.

***R2A3**: In our initial submission, we used the GCTA-COJO method to identify eQTLs and investigate the genetic architecture of gene expression because we also use this procedure to identify IVs in our MR-analyses. In this way, the initial results of the genetic architecture analysis directly inform how and when MR analysis is valid. As the reviewer indicates, GCTA-COJO is unlikely to find all the causal variants in a locus. We therefore followed the reviewer's suggestion and applied a statistical fine-mapping method (FINEMAP [1]) to the BIOS cohort and evaluated the genetic architecture of gene expression based this analysis. We applied FINEMAP [1] to 13,276 genes that pass our inclusion thresholds. The results show that likely causal variants are not often fully overlapping between genes (2.8% of all genes), while for a large fraction (33.2%), variants are in LD with variants of another genes. These results agree with our GCTA-COJO analysis, indicating that IVs of genes are much more likely to be in LD with other potentially pleiotropic eQTL variants than to be overlapping. The results of this analysis are reported on page 6, lines 115-124:*

"To strengthen our inferences on the genetic regulation of gene expression in cis, we performed statistical fine-mapping using FINEMAP v1.3.126 on 13,276 genes (Methods) (Supplementary Methods). Only 373 (2.8%) genes have full eQTL overlap (all variants in the top configuration of a gene are identical or in high LD ($r2 > 0.99$)), while 33.2% of the genes haves at least one variant in $r2 > 0.5$ LD with a variant in the top configuration of another gene. These percentages are higher for configurations with larger posterior inclusion probabilities (Methods) (Supplementary Table 1), but overall the results are similar to our observations from the GCTA-COJO analysis, i.e. the genetics of gene expression in whole blood is mostly regulated by variants that do not overlap but are in moderate LD with variants associated with gene expression changes of another gene. Based on these results, it seems likely that pleiotropy through LD is more common than pleiotropy through overlap in gene expression traits"

[1] Benner C. et al, Bioinformatics 2016, DOI: 10.1093/bioinformatics/btw018

R2Q4: Kudos for identifying independent signals for IV selection through GCAT-COJO. This is a much improved compared to the more standard MR LD clumping method.

R2A4: *We thank Reviewer #2 for the kind words in this matter, they are greatly appreciated*

R2Q5: My understanding is that the authors have used the conditional Betas for the exposure in their analysis (p.27,L.452). Why would you only include the conditional Betas for the exposure and not the outcome?

R2A5: *Thank you for this insightful question. In our MR-link analysis, we did indeed use conditional betas for our exposure, but since we use individual-level phenotype and genotype data for modeling the outcome (as described in equation 5 in the Methods section), we do not incorporate any betas from the outcome into the model. We have further specified this in the methods on page 23, line 512-513:*

"Because individual level data of the outcome is modeled by MR-link, MR-link does not use any summary statistics of the outcome."

R2Q6: I don't find Figure 2 helpful for explaining "Causality with pleiotropy through overlap" vs "causality with pleiotropy through LD". Please try improving the figure. It may help to show "LocusZoom" like plots? This point is quite important to the paper and deserves a full figure to clearly illustrate your definitions.

R2A6: *Thank you for pointing out the need for better graphical representation of pleiotropic scenarios, a concern also raised by Reviewer #1. As detailed in R1Q3, we have now separated Figure 2 into two figures. In the new Figure 2, we include a graphical representation of a locus and a diagram for each scenario to better explain the sources of pleiotropy in a cis locus. Additionally, we hope the new Figure 2 better depicts sources of unobserved pleiotropy. In the new Figure 3, we illustrate the results of the tested MR-methods on the simulations.*

R2Q7: Pleiotropy can refer to the effect a genetics signal has through multiple genes or across multiple outcomes. Here, it seems like the authors have defined "unknown pleiotropic exposure" as the effect of other genes in a locus on the outcome (Figure2B; p.27,L461; p.28,L.472). After looking at the methods, I believe each model represents just 1 gene eQTL in a pairwise analysis with the 1 GWAS and is modeling the effect of the other genes as "unknown pleiotropic exposure". However, these other genes *have* data on their effect on the outcome. It seems like the effect on the outcome of the other genes in the locus effect are being estimated instead of using the actual xQTL data that is in-hand (e.g. p.28,L472). Please confirm this isn't a misunderstanding and improve the text to address the issue.

***R2A7**: The reviewer is correct that we model pleiotropy as an effect of another phenotype that is regulated by the same locus. Although this data is "at hand" when we are simulating the actual outcome, and we could use it in our simulations, this is not the case in a real-world scenario. We cannot assume that every source of pleiotropy is measured. Pleiotropy from hard-to-measure tissues, developmental stages, and other important phenotypes cannot always be accounted for. To make a fair comparison in simulations of the pleiotropic cases, we discard the summary-level data of the unobserved exposure and assess the tested MR-methods when the source of pleiotropy is unknown. We have revised Figure 2 to make this clearer. Indeed, after reading the comments from both reviewers, we also recognize that the definition of the unobserved exposure was not always clear. We apologize for this and have tried to clarify this point. As detailed in R1A3, our concept of "unobserved exposure" refers to any source of pleiotropy that would originate from an unmeasured phenotype. We have specified this in Figure 2 and in the main text (page 4, lines 70-72).*

R2Q8: P.26,L.468 – Should there be an additional error term for the measurement error of the exposure?

R2A8: *Reviewer 2 is correct that we do not model an exposure error term in equation 4, as we do in equation 3 where we jointly model the error terms of the observed and unmeasured exposure (equations 1*

and 2). When we model the outcome in equation 4 using MR-link, we join these error terms together in a single variable because we are only interested in a causal effect driven by the genetic variants of the measured exposure.

Additional changes to the manuscript not requested by the reviewers

Change #1
We have revised our eQTL analysis in BIOS and noticed an issue in our code in selecting genes for GCTA-COJO, resulting in 2,947 genes being missed out. We have now fixed this and updated our results to include all 13,778 genes (previously 10,831) with an eQTL at $p < 5e-8$ for analysis.

This change does not affect the conclusions of the original manuscript regarding the genetic architecture, but we did identify 5 additional genes to be significant in the LDL-C analysis, while 2 genes (MIR4482-1 and PRDM5) no longer passed significance. Since this issue was only related to the BIOS cohort, it did not affect analyses and conclusions on simulations nor those on GTEx eQTLs.

Change #2
We have removed the permutation step in the MR-link analysis. In the new analysis described in change #1, all Bonferroni-significant genes passed our permutations. We therefore decided to remove this step from the manuscript and from the code implementation of MR-link. This change does not affect any of the conclusions of the manuscript nor the performance of MR-link.

Change #3
*In the section named "**eQTL variants between different genes are often in LD**" page4, line 90 we have used different thresholds for eQTL variants that are overlapping ($r^2 > 0.99$) and eQTL variants that are in LD ($r^2 > 0.5$). In the original manuscript we only reported on the latter threshold. This addition was made to better show the proportion of genes that can be sources of pleiotropy through overlap as compared to those that instead would be sources of pleiotropy through LD.*

Reviewers' comments:

Reviewer #1 (Remarks to the Author):

In their rebuttal letter, the Authors have addressed all the points I raised in my first review of the paper. Their answers, and the corresponding text amendments, appear to me to be satisfactory. I have no further comments to make. The amendments have made the manuscript clearer and the underlying method assumptions more explicit. As I said previously, the paper is valuable. As far as I am concerned, it is worth being published in its present form.

REFOMMENDATION: publish in its present form

Reviewer #2 (Remarks to the Author):

Overall this manuscript tries to improve some of the weak points of MR by addressing pleiotropy and conditional signals. The method is also appealing because it claims to be capable of analysis when only 1 IV is available, addressing a critical limitation of other popular MR methods. In addition, this submission of the manuscript is improved compared to the first. However, I still have concerns.

• Supplemental Figure 4 demonstrates that these results are largely consistent with previously available methods. While I think technically this method is an improvement over existing methods, I am not convinced that this method in practice ends up with significantly different results than available methods.
• I don’t think that he coloc analysis supporting Supplemental Figure 2 was run appropriately. One of the assumptions of coloc is the data contain independent signals with a causal variant. If IV selection for MR only used marginal GWAS results, then it would have been fair to only use coloc on the marginal GWAS. However, given that the GCAT-COJO was used for IV selection, the correct comparison should have also used the conditional beta & SE for coloc instead of the marginal results. It is also concerning that by adding simulated causal variants their data suggest that coloc increases the ability to detect causal variants (Supplemental Figure 2 A-D). Given the assumption of independent signals, adding variants should have decreased the true positive rate for coloc.
• The manuscript relies almost exclusively on simulated data. I question the utility and validity of these simulated data given that their method is able to predict with an AUC of 1.00 under non-pleiotropy conditions (Supplemental Figure 2 A-D).
• For a method that claims to identify disease causal genes with such precision, and with the availability of resources such as UKBiobank, I believe the method should be benchmarked using some of the ‘gold standard’ disease causal gene lists that are available (e.g. rare disease, successful drug targets, metabolomics, etc).

Reviewer #1 (Remarks to the Author):

In their rebuttal letter, the Authors have addressed all the points I raised in my first review of the paper. Their answers, and the corresponding text amendments, appear to me to be satisfactory. I have no further comments to make. The amendments have made the manuscript clearer and the underlying method assumptions more explicit. As I said previously, the paper is valuable. As far as I am concerned, it is worth being published in its present form.

RECOMMENDATION: publish in its present form

We thank this reviewer for his comments that helped us substantially improve the manuscript.

Reviewer #2 (Remarks to the Author):

Question 1

Q1. Overall this manuscript tries to improve some of the weak points of MR by addressing pleiotropy and conditional signals. The method is also appealing because it claims to be capable of analysis when only 1 IV is available, addressing a critical limitation of other popular MR methods. In addition, this submission of the manuscript is improved compared to the first. However, I still have concerns.

A1. We thank the reviewer for the positive feedback and the constructive criticisms which have helped to improve the manuscript. We would like also to remark that our method is capable of analysis when only 1 IV is available, assuming however that there isn't a pleiotropic effect with an overlapping ($r^2 > 0.95$) instrumental variable (IV). We have taken care to always mention this limitation in the text, this is mentioned specifically on page 6, lines 125-137 and on page 15, lines 327-334.

Question 2

Q2 Supplemental Figure 4 demonstrates that these results are largely consistent with previously available methods. While I think technically this method is an improvement over existing methods, I am not convinced that this method in practice ends up with significantly different results than available methods.

A2. As the reviewer pointed out, we show in Supplementary Figure 4 that the results obtained with MR-link and other MR methods are indeed very consistent in terms of effect direction and also often comparable in magnitude. However, the main message from Supplementary Figure 4 is the increased power of the MR-link method. In fact, in the majority of the cases the other methods are either underpowered or are unable to make a causal estimate at all due to lack of available IVs. Only one gene and only with one method (IVW) is significant after multiple testing correction. Therefore MR-link identifies significantly different results than other available methods.

We recognize that this was not immediately apparent in Supplementary Figure 4, and have therefore modified the figure (which is now Supplementary Figure 5) to display significance of each gene-method combination and also marked cases where a specific method was unable to make a causal estimate. Moreover, we have modified the text at page 10-11, lines 226-235 to specify the cases when a certain

method was unable to make an estimate due to the limited number of IVs (3 methods for up to 18 genes). The new section now reads:

"

*For all 18 genes, the effect direction estimated by MR-link was concordant with the direction estimated by other MR-methods when they were available, except in the case of MSLN, where only LDA-MR-Egger gave discordant results compared to all other methods (**Table 1**, **Supplementary Figure 5** and **Supplementary Table 9**). Interestingly, 17 of the 18 genes did not pass significance after multiple testing correction using the other tested methods: only ABO passed Bonferroni significance and only when using the IVW method (**Table 1**, **Supplementary Figure 5** and **Supplementary Table 9**). In 13 genes, a causal effect could not be estimated by: MR-Egger, LDA-MR-Egger and MR-PRESSO because there were too few IVs. Furthermore, MR-PRESSO did not make a causal estimate in the remaining 5 genes as it identified too many outliers (**Table 1**, **Supplementary Figure 5** and **Supplementary Table 9**)."*

Question 3

Q3 I don't think that the coloc analysis supporting Supplemental Figure 2 was run appropriately. One of the assumptions of coloc is the data contain independent signals with a causal variant. If IV selection for MR only used marginal GWAS results, then it would have been fair to only use coloc on the marginal GWAS. However, given that the GCAT-COJO was used for IV selection, the correct comparison should have also used the conditional beta & SE for coloc instead of the marginal results. It is also concerning that by adding simulated causal variants their data suggest that coloc increases the ability to detect causal variants (Supplemental Figure 2 A-D). Given the assumption of independent signals, adding variants should have decreased the true positive rate for coloc.

A3.1 We thank the Reviewer for a careful look at our coloc results. We have performed the coloc estimation according to the practices described in the coloc guide [1], which requires a marginal p value per SNP in the region without the need for any conditional analysis. An extended implementation of coloc is now available that is expected to be more accurate in the case of multiple independent variants [2]. This implementation uses conditional effect sizes (coloc-cond) as well as a LD-based pruning procedure that masks all variants in LD with the conditioned variant(s) (coloc-masked). In the case of multiple independent variants, the conditional or masking procedure is iteratively repeated, and the highest PP4 from all iterations is used as a statistic.

We have applied these three (coloc, coloc-cond and coloc-masked) methods to all our simulations. We are presenting performance of the different approaches in 2 ways: i) Using the area under the receiver operator characteristic (AUC) and ii) detection rate bar-plots for power and false positive rates in different scenarios, by using a coloc PP4 > 0.9 to declare a finding significant. We now report on these methods in Supplementary Figures 2 and 3 and in Supplementary Tables 5 and 6. The new Supplementary Figure 3 representing detection rates of the coloc methods is shown below. (Figure 1 in this document)

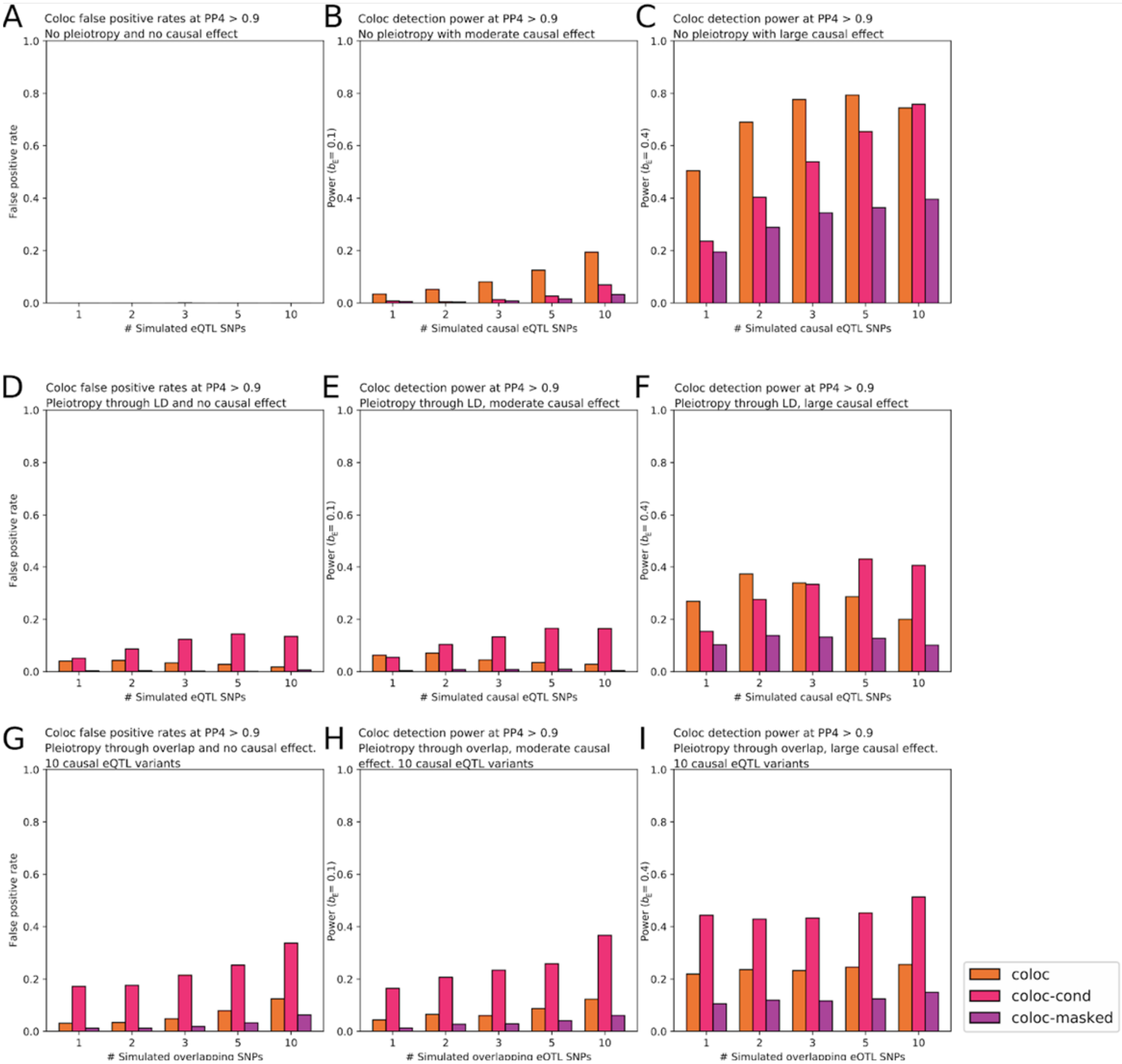

*Figure 1: This figure shows detection performance of coloc variations based on simulations representing no pleiotropy (**A-C**), pleiotropy through linkage disequilibrium (LD) scenarios (**D-F**) when 1, 3, 5 or 10 causal SNPs were simulated and increasing levels of pleiotropy through overlap combined with 10 causal variants(**G-I**) (**Methods**). (**A, D, G**) False positive rates (at coloc PP4 > 0.9) when no causal effect is simulated (b_E=0). (**B, E, H**) Detection power when a moderate causal effect is simulated (at coloc PP4 > 0.9) (b_E=0.1). (**C, F, I**) Detection power when a large causal effect is simulated (at coloc PP4 > 0.9) (b_E=0.4). Extended results can be found in **Supplementary Table 6**.*

As expected by the reviewer, the conditional method (coloc-cond) improves the power of coloc when multiple causal variants are simulated especially in pleiotropic scenarios. The masking procedure (coloc-masked) has lower power than the other coloc approaches, although the discriminative ability is very similar to the original coloc (Supplementary Figure 3) (Supplementary Table 5). It is worth noting that according to our simulation scheme, for the null scenario of no causal relationships and no pleiotropy, the outcome is completely independent of the genetic variants for the exposure, and thus the false positive rate for coloc is very close to zero. When there is no causal relationship with the exposure, but an effect of pleiotropy, the outcome is dependent on the pleiotropic effects, and thus a spurious association with

the variants associated with the exposure drives the false positive rates. The false positive rate is higher for the coloc-cond method, we believe this is due to an enhanced association at pleiotropic variants due to conditioning on only the variants associated with the exposure.
Next to the updated Supplementary Figures 2 and 3 and corresponding Supplementary Tables 5 and 6, we have updated the methods on pages 26 and 27, lines 579-594, and the results section on pages 8 and 9, lines 175-198. The results section now reads:

*"Finally, we compared MR-link to the coloc package using the area under the receiver operator characteristic curve (AUC) metric as well as FPRs and power (calculated using coloc PP4 > 0.9 as a threshold) (**Methods**). We used the AUC metric because coloc provides posterior probabilities of causal variant sharing and not p values (**Methods**). As coloc assumes that the exposure and the outcome share only one causal variant, we also included the newly implemented coloc variations (coloc-cond and coloc-masked) in our comparison. These variations are expected to perform better in scenarios with multiple causal variants[32]. When comparing MR-link to the coloc variations through the AUC metric, we find that MR-link consistently outperforms coloc and coloc-masked in all scenarios, and coloc-cond in pleiotropic scenarios. In non-pleiotropic scenarios, MR-link and coloc-cond have approximately the same performance (**Supplementary Figure 2**) (**Supplementary Table 5**). As expected, coloc-cond has better discriminative performance compared to the original coloc when multiple causal variants are simulated (**Supplementary Figure 2**) (**Supplementary Table 5**).*
*To illustrate detection rates in standard coloc settings as they may be used in a real-world analysis, we determined power and FPR for all coloc variations at a PP4 threshold of > 0.9 (**Supplementary Figure 3**) (**Supplementary Table 6**). In the non-pleiotropic case, coloc and coloc-cond have the best detection power (up to 0.79 for coloc and 0.76 for coloc-cond), combined with near zero FPRs (max: 0 for coloc and 0.0006 for coloc-cond) while coloc-masked has lower power (up to 0.40) with a zero FPR (**Supplementary Figure 3A-C**) (**Supplementary Table 6**). In simulations of pleiotropy through LD, all coloc methods have increased FPRs (medians: 0.026 for coloc, 0.142 for coloc-cond and 0.0037 for coloc-masked) with a decrease in power relative to the non-pleiotropic simulations (max: 0.37 for coloc, 0.43 for coloc-cond and 0.14 for coloc-masked) (**Supplementary Figure 3D-F**) (**Supplementary Table 6**). These patterns were even more apparent in cases of pleiotropy through overlap (**Supplementary Figure 3G-I**) (**Supplementary Table 6**). This comparison through FPRs and power indicates again that MR-link has superior discriminative ability over coloc variations, especially in the presence of pleiotropy."*

A3.2 Furthermore, we have also investigated the reasons behind the increasing discriminative ability of coloc with the increasing number of causal variants simulated, as we agree that it seems counterintuitive with the single causal variant assumption of coloc. This can be attributed to the characteristics of our simulation schemes; we have elaborated this below.

The simulations we have performed in the original version of the manuscript selected causal variants for the exposure randomly across the region and selected their effect size on the exposure randomly as well. This setting resulted in sets of causal variants that i) were not always in LD with one another and ii) had differing effect sizes resulting in differences in variance explained. We believe this way of simulating is more biologically plausible than simulating variants with equal variance explained, as two variants are unlikely to have the exact same effect on a phenotype. The difference in variance explained of each causal variant and the low LD between them explains our observations; Giambartolomei et al. report that the

single variant assumption is not strongly violated if variants do not have equal variance explained (section named 'Dealing with several independent associations for the same trait' of [3]): "the presence of additional associations that explain a smaller fraction of the variance of the trait, for example additional and independently associated variants, have a negligible impact on coloc computations."

Our simulations will rarely result in an equal variance explained or highly linked causal variants, and therefore we do not see a decrease in discriminative ability when analyzing scenarios with increasing numbers of causal variants.
To illustrate this, we have simulated increased LD between the (simulated) causal eQTL variants (min pairwise $r^2 > 0.75$) to increase the probability that 2 causal variants have similar variance explained, and the effect of the violation of the multiple causal variant assumption is more pronounced. We compare coloc in our original simulations to high LD simulations in Figure 2 in this document.
When there is no pleiotropy simulated and the causal effect is large ($b_{\mathrm{E}} = 0.4$, Figure 2C), the power of coloc is lower in the high LD case than in the original simulations, indicating that the single variant assumption of coloc is violated more strongly in the high LD case. This behaviour is not transferrable to the simulations with the moderate causal effect ($b_{\mathrm{E}}=0.1$, Figure 2B) as we see an increase in power in the high LD simulations. This increase in power is likely due to multiple causal eQTLs with different variance explained that jointly make a small region more significant (as the increased LD can make the eQTL effect more pronounced). This increase in significance in a region makes the coloc test more powerful.
When simulating pleiotropy through LD (Figure 2D-F), we see that the single variant assumption is more strongly violated in the high LD variation: false positives are higher and power is lower (relative to the false positive rate) when adding more and more causal variants. The reason this effect is more pronounced in the pleiotropy through LD region is probably due the large significant effect in the regions through the addition of the pleiotropic effects.

In summary, these simulation show that i) the performance of coloc is decreased in high-LD scenarios compared to our original simulations when the causal effect is large and thus each single variant explains a larger fraction of the outcome variability, and ii) presence of pleiotropy decreases power when number of causal variants increases, a pattern not observed in absence of pleiotropy. We re-iterate that scenarios with absence of pleiotropy and a large number of causal variants are unlikely to exists in real data, as pleiotropy is expected to affect the majority of traits. This indicates that in real data scenarios the coloc performance is expected to decrease as the number of variants increases.

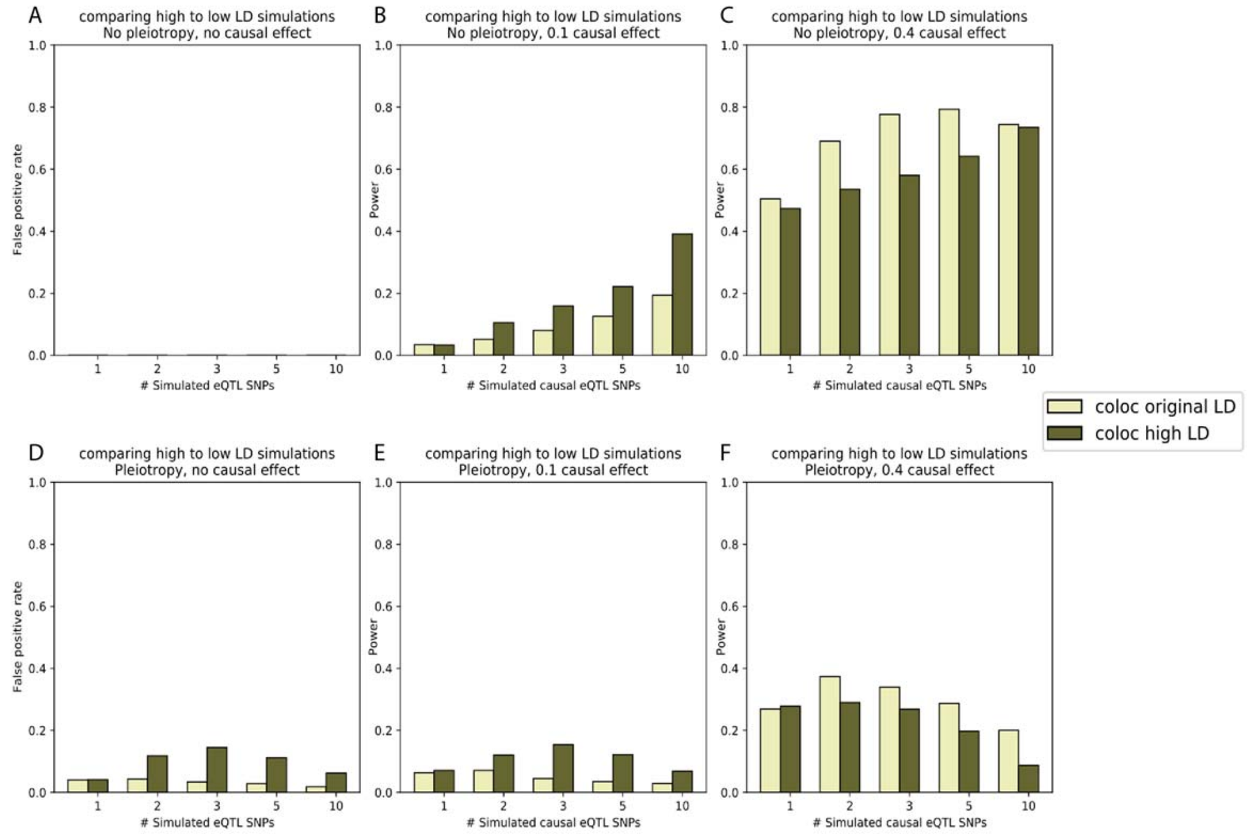

*Figure 2: This figure shows the performance of coloc in our simulations where the causal variants are randomly selected in a 1Mb region (The original simulation scheme of our manuscript, light yellow bars), compared to simulations where the causal variants are selected to be in pairwise 0.75 R^2 LD (dark brown bars). A coloc positive is defined as having a PP4 > 0.9. Panels **A-C** depict simulations with no pleiotropy, whereas the panels **D-F** depicts scenarios with pleiotropy through LD. The left column (panels **A** and **D**) show false positive rates when there are no causal effects. The middle column (panels **B** and **E**) represents scenarios with a moderate causal effect and the right column (panels **E** and **F**) shows a scenario with a large causal effect.*

We have decided to not include this additional figure and simulation schemes into the manuscript, as we think that it mostly highlights the behavior of variant colocalization methods in certain circumstances, which is out of scope for our manuscript focused on Mendelian Randomization methods.

[1] https://cran.r-project.org/web/packages/coloc/vignettes/vignette.html, see section named "(Approximate) Bayes Factor colocalisation analyses", here a linear regression is fit on the dataset per SNP using either the lm() function of R, or the more intuitively named function single.snp.tests() from the snpStats R package (https://www.bioconductor.org/packages/release/bioc/html/snpStats.html) .

[2] Wallace C (2020) Eliciting priors and relaxing the single causal variant assumption in colocalisation analyses. PLOS Genetics 16(4): e1008720. https://doi.org/10.1371/journal.pgen.1008720

[3] Giambartolomei C, Vukcevic D, Schadt EE, Franke L, Hingorani AD, et al. (2014) Bayesian Test for Colocalisation between Pairs of Genetic Association Studies Using Summary Statistics. PLOS Genetics 10(5): e1004383. https://doi.org/10.1371/journal.pgen.1004383

Question 4

Q4• The manuscript relies almost exclusively on simulated data. I question the utility and validity of these simulated data given that their method is able to predict with an AUC of 1.00 under non-pleiotropy conditions (Supplemental Figure 2 A-D).

A4. Simulated data is essential to explore performance of a statistical method across a wide range of different scenarios, for which otherwise individual level data would be difficult to gather.
We acknowledge that simulations are never fully representative of real data. We designed these simulations to reflect our understanding of the genetic architecture of gene expression, and based on insights that we gained by looking at the large blood transcriptomic data sets that we have access to. Our manuscript heavily relies on simulated data, and we therefore have complemented this with two applications to real data.

In some of the simulated scenarios, the AUC of certain methods reached very high values, near 1.0. Interestingly, the previously discussed coloc-cond method also identifies AUCs that are rounded to 1.0. We remark that these simulation scenarios are some of the most extreme examples for causal effects. For most genes it's unlikely that there are more than 3 causal variants and that none of these have a pleiotropic effect and the causal effect is high (b_E=0.4). In scenarios where the causal effect is lower or there is pleiotropy, the AUC for our method is much lower. We acknowledge that the AUC curve also isn't directly related to a specific cut-off on either PP4 for coloc or p value for MR-link, and thus may be less directly interpretable than classical power and false positive ratio estimates, as those presented in our Figure 3. We have therefore added, as discussed in the answer of Q3, a figure that shows the performance of coloc methods using false positive rate and power, in the same style as Figure 3 (Supplementary Figure 6) and the data of these analyses is listed in Supplementary Table 3.

Question 5

For a method that claims to identify disease causal genes with such precision, and with the availability of resources such as UKBiobank, I believe the method should be benchmarked using some of the 'gold standard' disease causal gene lists that are available (e.g. rare disease, successful drug targets, metabolomics, etc).

A5. We agree with the reviewer that UKBiobank is a great resource for MR-link applications, but unfortunately we do not currently have access to individual level data of UKBiobank. We would like to remark that in contrast to other 2-samples MR methods, MR-link requires individual level data of the outcome and therefore we cannot explore this further for our manuscript given time constraint for revisions. Furthermore, the current MR-link implementation focuses on quantitative traits and we have not yet explored performance of MR-link on a binary outcome. Therefore we prefer to avoid its application to either common or rare diseases, limiting possibilities for benchmarking our method in "gold standard" disease causal gene lists.

We have however explored additional applications of MR-link to proteomics QTLs (pQTLs) data that could be jointly analyzed with the LDL-C levels in the Lifelines cohort available to us. We expect this pQTLs dataset to highlight genes that are well known in the causal pathways of this lipoprotein metabolism, such as ApoB and ApoE.

We have used pQTL summary statistics of 3,283 cardiometabolic proteins provided by Sun et al. [1]. Levels of these proteins were measured in plasma using the so-called SOMAmer measurements; these are measurements based on the protein binding to specially constructed oligonucleotides named SOMAmers.

After extracting genotype variants that overlap with variants available in the Lifelines cohort, we found 471 proteins with at least one significant ($p < 5 \times 10^{-8}$) pQTL. We have run MR-link on these pQTLs summary statistics combined with the Lifelines cohort genotype data and LDL-C measurements, and identified one protein that passes the Bonferroni threshold: ApoE isoform ApoE3, with two instrumental variables. Of note, another isoform of APOE was measured: ApoE2, which did not pass our stringent Bonferroni threshold, but it was the 6th most significant protein in the list. In both cases, we observed a positive causal effect of ApoE on LDL-C, which is consistent with current knowledge. Furthermore, our observations are consistent with the known higher impact of E3 and E4 isoforms compared to E1 and E2. Unfortunately, there were no pQTLs available for APOB, therefore, it could not be tested with MR-link. We also noticed that ABO was measured as protein, pQTLs were available but a causal estimate was not significant.

We are sharing the table with the full results as an attachment to this letter for the reviewer's interest, while in the manuscript we describe the results within the text, with a new paragraph at page 12-13, lines 269-286 that reads as follows:

*"**MR-link confirms ApoE changes affect LDL-C levels***
To assess the effectiveness of MR-link in proteomics measurements, we combined the aforementioned LDL-C measurements in the Lifelines cohort with cis-pQTL summary statistics of 471 plasma protein measurements (measured using the SOMAscan platform in a cohort of 3301 individuals) (Methods). One protein passes the Bonferroni multiple testing threshold (p < 1.05 x 10-4): ApoE3, an isoform of ApoE (causal effect: 0.40 (+/- 0.13), p=4.65 x 10-5, SOMAmer ID: APOE.2937.10.2). pQTLs were also available for ApoE2 (SOMAmer ID: APOE.5312.49.3), another isoform of ApoE but the causal effect was weaker and did not pass the Bonferroni threshold (causal effect= 0.56 (+/- 0.24), p=0.002)44. These results are in line with the well-known causal relationship between increased ApoE plasma levels and LDL-C, and the widely described stronger impact of the E3 isoform compared to the E2 isoform. Interestingly, MR-link did not estimate BGAT, the protein product of ABO, to be significant in this dataset (SOMAmer ID: ABO.9253.52.3, p=0.18) We compared the IVs identified for BGAT (rs9411463 and rs72775494) with those used in the ABO blood eQTL analysis and found that only one IV for the BGAT protein was in LD (rs9411463) with any of the four IVs for ABO expression in BIOS. This scenario is in line with the overall patterns observed in the proteomics study - only a small fraction of eQTLs in blood also affect protein levels, but our results could also reflect targeting of the SOMAmer to a specific ABO protein isoform. Unfortunately, further isoform information for BGAT was not available in the original study.
"

[1] Sun, Benjamin B., Joseph C. Maranville, James E. Peters, David Stacey, James R. Staley, James Blackshaw, Stephen Burgess, et al. (2018) Genomic Atlas of the Human Plasma Proteome. Nature 558 (7708): 73–79. https://doi.org/10.1038/s41586-018-0175-2.

Further changes to the manuscript in this resubmission

- *We have further elaborated the p value calibration steps in Supplementary Note 1 and have added an analysis when the assumptions of p value calibration are violated*
- *We have updated the colours of arrows in Figure 4 to avoid confusion for colour-blind readers*

somamer_name	ensembl_name	beta	se	n_ivs	calibrated p value
APOE.2937.10.2	ENSG00000130203	0.40001	0.12619	2	4.65264E-05
FCRL3.4440.15.2	ENSG00000160856	0.40456	0.14111	1	0.000210551
FCGR2A.3309.2.2	ENSG00000143226	-0.01904	0.00769	1	0.001220147
LMNB1.3889.64.2	ENSG00000113368	0.0566	0.02362	1	0.001710255
FAH.11424.4.3	ENSG00000103876	-0.06435	0.02718	2	0.001921805
APOE.5312.49.3	ENSG00000130203	0.56503	0.24775	1	0.002726039
LILRB2.5633.65.3	ENSG00000131042	0.11284	0.05018	1	0.00309371
PRTN3.3514.49.2	ENSG00000196415	0.03741	0.01784	1	0.005506988
APOL1.11510.31.3	ENSG00000100342	0.05726	0.03211	2	0.016632917
CBR1.12381.26.3	ENSG00000159228	-0.0625	0.03591	2	0.019153948
PRTN3.13720.95.3	ENSG00000196415	0.03055	0.01761	1	0.019495904
ITIH1.7955.195.3	ENSG00000055957	-0.06812	0.04088	1	0.024280511
NELL1.6544.33.3	ENSG00000165973	0.04866	0.03043	1	0.029894734
CLIC5.12475.48.3	ENSG00000112782	-0.051	0.03272	1	0.033873972
NQO1.9837.60.3	ENSG00000181019	-0.05593	0.03668	2	0.037483802
TMEM132A.7871.16.3	ENSG00000006118	-0.04401	0.02997	3	0.044266507
CX3CL1.2827.23.2	ENSG00000006210	0.0687	0.04693	1	0.044885089
LILRB5.7015.8.3	ENSG00000105609	0.05027	0.03455	2	0.046034944
SERPINA10.6583.67.3	ENSG00000140093	0.02655	0.01829	1	0.046468756
ENTHD2.7947.19.3	ENSG00000167302	-0.0434	0.03034	1	0.049347672
IL18R1.3446.7.2	ENSG00000115604	-0.04053	0.02932	3	0.056624657
CCL14.2900.53.3	ENSG00000213494	0.07595	0.05651	1	0.063006698
SCG3.7957.2.3	ENSG00000104112	-0.06531	0.04898	2	0.064842914
TNXB.5698.60.3	ENSG00000168477	-0.04326	0.03302	4	0.069125343
NMRAL1.13988.67.3	ENSG00000153406	0.04962	0.03793	1	0.069458762
F10.3077.66.2	ENSG00000126218	0.05738	0.04392	1	0.069792749
CACNA2D3.8885.6.3	ENSG00000157445	0.04414	0.0373	1	0.096602634
IDUA.3169.70.2	ENSG00000127415	-0.05685	0.04818	9	0.097417204
PF4V1.5663.18.3	ENSG00000109272	-0.06263	0.05309	1	0.097479965
TDGF1.5810.25.3	ENSG00000241186	-0.03099	0.02631	3	0.097982576
PLA2G2A.2692.74.2	ENSG00000188257	-0.0193	0.01645	1	0.09905371
TCN1.11232.46.3	ENSG00000134827	-0.06941	0.05915	2	0.09905371
MMP12.4496.60.2	ENSG00000110347	0.04183	0.03614	1	0.103123684
AKR1C1.12618.50.3	ENSG00000187134	-0.03565	0.03083	2	0.103444134
LGALS4.2982.82.2	ENSG00000171747	0.07406	0.06434	1	0.104858372
SERPINF1.9211.19.3	ENSG00000132386	0.04544	0.03956	1	0.105503498
GPNMB.5080.131.3	ENSG00000136235	0.06212	0.05432	1	0.106862909
POFUT1.5634.39.3	ENSG00000101346	-0.0228	0.01997	1	0.107382433
NPTX1.9256.78.3	ENSG00000171246	0.04384	0.03849	1	0.108033118
B3GAT3.6897.38.3	ENSG00000149541	0.04558	0.04102	1	0.115884843
C1QC.14100.63.3	ENSG00000159189	0.04881	0.04399	5	0.116352912
THSD1.5621.64.3	ENSG00000136114	0.03613	0.03258	1	0.116553722
KLK12.3199.54.2	ENSG00000186474	0.03021	0.02731	1	0.117358222
TNFRSF1A.2654.19.1	ENSG00000067182	-0.04338	0.03933	1	0.118232032
TIMP4.6462.12.3	ENSG00000157150	-0.04407	0.03997	1	0.118299346
SERPINF1.7735.17.3	ENSG00000132386	0.04199	0.03818	1	0.119108198
SIGLEC9.3007.7.2	ENSG00000129450	-0.01226	0.01116	1	0.119445811

IFI16.12893.159.3	ENSG00000163565	0.03125	0.02874	1	0.122841039
DEFB1.6629.3.3	ENSG00000164825	-0.05378	0.04972	3	0.124620234
CPA4.9267.2.3	ENSG00000128510	0.03276	0.03057	5	0.127652402
PLA2R1.10916.44.3	ENSG00000153246	0.02537	0.02374	2	0.128622761
IL1RAP.2630.12.2	ENSG00000196083	-0.05468	0.05127	4	0.129317519
ART3.10970.3.3	ENSG00000156219	-0.04941	0.04667	2	0.131829971
PTGDS.10514.5.3	ENSG00000107317	0.05167	0.04899	1	0.133092829
CLPS.5749.53.3	ENSG00000137392	0.04296	0.04082	1	0.133796323
RNASE4.5644.60.3	ENSG00000258818	-0.03795	0.0361	1	0.134219072
GPNMB.8240.207.3	ENSG00000136235	0.05718	0.05464	1	0.135773335
MBL2.3000.66.1	ENSG00000165471	-0.04307	0.04245	5	0.146399171
CNTFR.14101.2.3	ENSG00000122756	-0.03812	0.03802	1	0.150643451
NTN1.6649.51.3	ENSG00000065320	-0.03757	0.03748	2	0.150717022
MPO.2580.83.2	ENSG00000005381	0.04662	0.04725	1	0.156347206
UCMA.10977.55.3	ENSG00000165623	-0.05996	0.0615	3	0.160770672
ART3.7970.315.3	ENSG00000156219	-0.04844	0.05003	2	0.163264799
REG4.11102.22.3	ENSG00000134193	-0.05182	0.05372	3	0.164631258
CTSZ.4971.1.1	ENSG00000101160	-0.0331	0.03432	1	0.164631258
CCL23.2913.1.2	ENSG00000167236	-0.04263	0.04431	4	0.165544589
CHIT1.3600.2.3	ENSG00000133063	-0.06658	0.06922	3	0.165696995
IL1RAP.14048.7.3	ENSG00000196083	-0.05331	0.05545	3	0.165849452
CBR3.14091.42.3	ENSG00000159231	0.03269	0.0341	2	0.166918122
IL17RB.5084.154.3	ENSG00000056736	0.06815	0.07118	2	0.167453418
IGLL1.6485.59.3	ENSG00000128322	-0.05809	0.06082	1	0.168295891
FAM3B.9177.6.3	ENSG00000183844	-0.03737	0.03914	5	0.168449238
SEMA6A.7945.10.3	ENSG00000092421	-0.08099	0.08487	1	0.168679356
SERPING1.4479.14.2	ENSG00000149131	0.04901	0.05147	2	0.169524127
IL6R.4139.71.2	ENSG00000160712	-0.01075	0.0113	1	0.169908636
BST1.4535.50.2	ENSG00000109743	-0.04088	0.04321	8	0.171836068
ABO.9253.52.3	ENSG00000175164	0.08926	0.09621	2	0.179155908
TIE1.2844.53.2	ENSG00000066056	0.04916	0.05323	1	0.180806837
OAS1.10361.25.3	ENSG00000089127	-0.04215	0.04565	1	0.181043149
IGFBP7.3320.49.2	ENSG00000163453	0.03997	0.0434	1	0.181910622
GFRA2.2515.14.3	ENSG00000168546	0.07713	0.08401	1	0.183096057
VEGFA.2597.8.3	ENSG00000112715	0.02942	0.03215	1	0.184443058
AMICA1.8232.90.3	ENSG00000160593	0.02972	0.03263	3	0.186191762
CCL25.2705.5.2	ENSG00000131142	-0.0339	0.03743	5	0.18826644
CRISP2.9282.12.3	ENSG00000124490	-0.04524	0.05016	2	0.189868257
SECTM1.13093.6.3	ENSG00000141574	-0.0553	0.06137	1	0.190349803
IGFBP3.2571.12.3	ENSG00000146674	0.03711	0.04129	1	0.191314281
MAPK13.5006.71.1	ENSG00000156711	-0.04098	0.04561	1	0.191314281
CTRB1.5671.1.3	ENSG00000168925	0.03827	0.0429	3	0.194218787
GPX7.8345.27.3	ENSG00000116157	0.03343	0.03762	1	0.195677261
SIRPG.9241.40.3	ENSG00000089012	0.03166	0.03568	1	0.196245564
BCAM.2816.50.2	ENSG00000187244	-0.18454	0.2082	1	0.196651878
NTNG1.5637.81.3	ENSG00000162631	0.01496	0.01693	1	0.197791256
LILRB1.5090.49.2	ENSG00000104972	-0.03492	0.03994	5	0.201962995
NAGK.3894.15.2	ENSG00000124357	-0.05067	0.05832	1	0.204432436

GLRX2.12486.8.3	ENSG00000023572	-0.00625	0.00727	1	0.208739903
PTGR1.13543.7.3	ENSG00000106853	0.04198	0.04885	4	0.208739903
SPOCK3.9906.21.3	ENSG00000196104	0.04768	0.05567	3	0.210072182
CSF2RB.10512.13.3	ENSG00000100368	0.02307	0.02754	1	0.218895701
NT5C.12560.9.3	ENSG00000125458	-0.02431	0.02904	1	0.219234591
COL11A2.11278.4.3	ENSG00000204248	0.02721	0.03283	2	0.22306101
MXRA7.8005.1.3	ENSG00000182534	0.03092	0.03732	1	0.223317012
SAA1.4336.2.1	ENSG00000173432	-0.02173	0.02627	2	0.223914792
IL6R.8092.29.3	ENSG00000160712	-0.00932	0.01131	1	0.225369112
H6PD.7161.25.3	ENSG00000049239	-0.03473	0.04213	1	0.225369112
CCL25.14068.29.3	ENSG00000131142	-0.04289	0.05235	4	0.227772395
IL15RA.14054.17.3	ENSG00000134470	0.03567	0.04452	1	0.236785144
SERPINA4.14105.5.3	ENSG00000100665	-0.03332	0.04165	5	0.237309252
SOD3.8463.2.3	ENSG00000109610	0.01115	0.01395	1	0.237746353
UNC5C.5139.32.3	ENSG00000182168	-0.02509	0.03164	1	0.240990644
ST3GAL6.6947.4.3	ENSG00000064225	-0.03693	0.0469	5	0.243722034
GLCE.7808.5.3	ENSG00000138604	-0.02085	0.0265	4	0.24425208
C1QTNF5.7810.20.3	ENSG00000223953	-0.03231	0.04184	1	0.251898874
SLAMF7.5487.7.3	ENSG00000026751	-0.02345	0.0304	2	0.252256789
FUT5.4549.78.2	ENSG00000130383	0.03254	0.04257	4	0.255936931
MFGE8.4455.89.2	ENSG00000140545	0.02493	0.03261	1	0.256026953
SWAP70.13552.7.3	ENSG00000133789	-0.04777	0.06255	1	0.256297093
BCAN.3461.58.1	ENSG00000132692	-0.02193	0.02879	2	0.257378777
PCSK1.13388.57.3	ENSG00000175426	0.01273	0.01694	1	0.263178095
FRZB.13740.51.3	ENSG00000162998	-0.02875	0.03839	4	0.264362323
GZMB.14041.13.3	ENSG00000100453	-0.04285	0.05762	1	0.267286319
NRP1.3214.3.2	ENSG00000099250	0.03727	0.05018	1	0.267744346
PDCD5.12517.52.3	ENSG00000105185	0.04041	0.05454	2	0.268753102
LILRA6.7059.14.3	ENSG00000244482	-0.02672	0.03645	7	0.273172586
FGF7.14031.18.3	ENSG00000140285	0.03336	0.04576	1	0.275485766
FAM3B.5618.50.3	ENSG00000183844	-0.03133	0.04303	2	0.276042087
CDON.4541.49.2	ENSG00000064309	0.02453	0.03392	1	0.278830416
RET.3220.40.2	ENSG00000165731	0.02629	0.03637	1	0.279016703
CNTN2.3296.92.2	ENSG00000184144	0.0242	0.03364	2	0.280882312
MANSC1.9557.5.3	ENSG00000111261	0.02687	0.03734	1	0.280882312
KLK7.3378.49.2	ENSG00000169035	0.02858	0.0398	4	0.28172346
GUCA2B.6223.5.3	ENSG00000044012	0.03737	0.05225	1	0.283408783
NAAA.3173.49.2	ENSG00000138744	0.04216	0.05907	2	0.284252956
XXYLT1.6375.75.3	ENSG00000173950	-0.02833	0.03983	1	0.285756198
RPN1.10490.3.3	ENSG00000163902	-0.02053	0.02896	1	0.287168382
SERPINA4.3449.58.2	ENSG00000100665	-0.03151	0.04452	6	0.287639733
IGFLR1.7244.16.3	ENSG00000126246	-0.02764	0.04018	2	0.299428787
KDELC2.8296.117.3	ENSG00000178202	-0.01551	0.02267	1	0.301828803
PPIL1.9884.8.3	ENSG00000137168	-0.01691	0.02472	1	0.301924966
RELT.5115.31.3	ENSG00000054967	-0.03655	0.05344	1	0.301924966
MMP10.8479.4.3	ENSG00000166670	0.02384	0.03493	1	0.30279099
TNFAIP6.5036.50.1	ENSG00000123610	0.04345	0.06461	1	0.308978485
SPINK6.5731.1.3	ENSG00000178172	-0.04526	0.06818	2	0.314336656

CXCL1.2985.35.1	ENSG00000163739	0.01096	0.01657	1	0.315706578
PSAPL1.8814.33.3	ENSG00000178597	0.02349	0.03569	3	0.317962474
PCOLCE2.6081.52.3	ENSG00000163710	0.01992	0.0306	1	0.322494076
ENTPD1.7999.23.3	ENSG00000138185	-0.02793	0.04294	1	0.322691702
VEGFC.3132.1.1	ENSG00000150630	-0.01564	0.0242	1	0.32536454
MLN.5631.83.3	ENSG00000096395	-0.01805	0.02795	2	0.32586051
ASPN.6451.64.3	ENSG00000106819	0.04585	0.07172	4	0.330038998
SEMA3G.5628.21.3	ENSG00000010319	-0.04297	0.06771	1	0.333037144
CFHR5.3666.17.4	ENSG00000134389	-0.02156	0.03434	3	0.337454812
CTSH.8465.52.3	ENSG00000103811	-0.01865	0.02976	2	0.338361415
HYAL1.8309.12.3	ENSG00000114378	-0.02745	0.04402	1	0.340278689
CXCL11.3038.9.2	ENSG00000169248	-0.03714	0.05974	2	0.341593118
UGT1A6.7891.45.3	ENSG00000167165	0.03048	0.04907	2	0.341896749
ASAH2.3212.30.3	ENSG00000188611	0.01688	0.02728	1	0.343416598
CCL23.3028.36.2	ENSG00000167236	-0.0324	0.05242	4	0.343923842
FCN2.3313.21.2	ENSG00000160339	-0.02016	0.03274	1	0.345549134
LILRB2.5091.28.3	ENSG00000131042	-0.0177	0.02879	4	0.346159447
CA3.3799.11.2	ENSG00000164879	-0.02917	0.04747	1	0.346362985
COLEC10.6558.5.3	ENSG00000184374	0.03024	0.04949	1	0.348605226
TIRAP.9839.148.3	ENSG00000150455	-0.03334	0.05519	1	0.353415867
FAM20A.6433.57.3	ENSG00000108950	-0.02532	0.04246	1	0.35856412
CFHR5.7885.17.3	ENSG00000134389	-0.01956	0.03282	2	0.358874018
PDGFRB.3459.49.2	ENSG00000113721	0.0315	0.05318	1	0.361564561
POSTN.6645.53.3	ENSG00000133110	-0.02342	0.03958	2	0.361979248
ARSB.3172.28.2	ENSG00000113273	0.03031	0.05128	1	0.362394138
QPCTL.8866.53.3	ENSG00000011478	0.02819	0.04773	1	0.362601659
MTRF1L.11134.30.3	ENSG00000112031	0.02543	0.04311	1	0.363224524
LAMC2.9580.5.3	ENSG00000058085	-0.0254	0.04372	4	0.369478232
NCAM2.6507.16.3	ENSG00000154654	-0.02359	0.04075	1	0.370839213
NCR3.3003.29.2	ENSG00000204475	-0.02281	0.03941	1	0.371048785
BPI.4126.22.1	ENSG00000101425	-0.0229	0.03958	2	0.37115359
MANSC4.9578.263.3	ENSG00000205693	0.01966	0.03412	1	0.372937216
FCRL6.6617.12.3	ENSG00000181036	0.03333	0.05788	1	0.373147295
VTN.13125.45.3	ENSG00000109072	0.00644	0.01122	1	0.374619274
CPNE1.5346.24.3	ENSG00000214078	0.0391	0.06832	9	0.375566861
CCBL2.12682.5.3	ENSG00000137944	0.03465	0.06094	1	0.37820445
MIA.2687.2.1	ENSG00000261857	-0.01859	0.03281	3	0.379579139
GNMT.14006.36.3	ENSG00000124713	0.01671	0.0295	1	0.379684973
IL1RL2.2994.71.2	ENSG00000115598	0.02289	0.04045	1	0.380108438
TNFRSF6B.5070.76.3	ENSG00000243509	-0.03181	0.0573	1	0.388086188
APCS.2474.54.5	ENSG00000132703	-0.02429	0.04382	2	0.388620592
FHIT.9826.135.3	ENSG00000189283	0.01944	0.03539	1	0.392263027
DLL1.5349.69.3	ENSG00000198719	-0.01994	0.03646	1	0.394197371
DNAJC30.7866.11.3	ENSG00000176410	0.02856	0.05267	1	0.397430517
ICOSLG.9303.9.3	ENSG00000160223	0.02588	0.0482	4	0.401433997
SEMA3E.5363.51.3	ENSG00000170381	0.02397	0.04478	6	0.40262762
ASPH.6998.106.3	ENSG00000198363	-0.02424	0.04537	1	0.403496689
GHR.2948.58.2	ENSG00000112964	-0.02764	0.05185	1	0.404257801

FSTL1.13112.179.3	ENSG00000163430	0.01752	0.03287	1	0.404366583
B4GALT1.13381.49.3	ENSG00000086062	0.01697	0.03221	1	0.409056426
DNAJB11.7110.2.3	ENSG00000090520	0.03108	0.05932	1	0.411245896
CXCL16.2436.49.4	ENSG00000161921	-0.03391	0.06511	3	0.413660301
SIGLEC12.10037.98.3	ENSG00000254521	-0.03103	0.06034	6	0.418728912
GFRA1.3314.74.2	ENSG00000151892	-0.01724	0.03368	1	0.420498362
CHI3L1.11104.13.3	ENSG00000133048	-0.02281	0.04481	2	0.422714867
POMGNT2.6359.50.3	ENSG00000144647	0.02092	0.04132	1	0.424825385
S100A4.14116.129.3	ENSG00000196154	0.02823	0.05623	1	0.428167394
NUDT9.9482.110.3	ENSG00000170502	0.02482	0.04956	1	0.429172296
SIGLEC14.8248.222.3	ENSG00000254415	-0.02715	0.05476	9	0.433090353
SPARCL1.13707.27.3	ENSG00000152583	0.02952	0.06021	1	0.437587866
FAM213A.13423.94.3	ENSG00000122378	0.01969	0.0403	1	0.439054101
TPSB2.3403.1.2	ENSG00000197253	0.01553	0.03182	6	0.43939278
LSAMP.2999.6.2	ENSG00000185565	-0.02406	0.04965	1	0.442106496
CTSV.3364.76.2	ENSG00000136943	-0.02729	0.05632	1	0.442106496
KLK14.8620.56.3	ENSG00000129437	-0.02152	0.04444	3	0.442446246
STX7.8274.64.3	ENSG00000079950	0.01737	0.0364	1	0.448012405
PEAR1.8275.31.3	ENSG00000187800	-0.02784	0.05883	1	0.451207402
ART4.6576.1.3	ENSG00000111339	0.01575	0.03358	1	0.454756922
TNC.4155.3.2	ENSG00000041982	0.00883	0.01889	1	0.455789835
RTN4R.5105.2.3	ENSG00000040608	-0.01735	0.03748	2	0.459817039
WFIKKN2.3235.50.2	ENSG00000173714	0.01656	0.03619	2	0.464092271
AKR1A1.4192.10.2	ENSG00000117448	-0.00724	0.01586	1	0.465134982
CHST9.11646.4.3	ENSG00000154080	-0.02012	0.04431	1	0.467107532
ERLEC1.8957.72.3	ENSG00000068912	-0.02107	0.04708	1	0.472464643
GALP.9398.30.3	ENSG00000197487	0.01958	0.04389	1	0.473866875
CD59.11514.196.3	ENSG00000085063	0.01697	0.03806	1	0.473983816
GPC5.4991.12.1	ENSG00000179399	0.01241	0.02803	2	0.476559967
CPM.7768.10.3	ENSG00000135678	0.01896	0.04288	1	0.477146377
TXNDC5.11212.7.3	ENSG00000239264	0.02287	0.05176	1	0.477381036
SELP.4154.57.2	ENSG00000174175	0.01422	0.03226	1	0.478202774
POSTN.6650.20.3	ENSG00000133110	-0.01902	0.0432	3	0.47867264
SMPDL3A.14086.11.3	ENSG00000172594	-0.0089	0.02036	1	0.48149643
IL1RL1.4234.8.2	ENSG00000115602	-0.01946	0.04461	4	0.482203611
EREG.4956.2.1	ENSG00000124882	-0.04282	0.0985	1	0.483501393
SVEP1.11109.56.3	ENSG00000165124	0.01949	0.04493	3	0.484209975
LEPR.5400.52.3	ENSG00000116678	-0.01647	0.03818	3	0.486457084
RARRES1.8398.277.3	ENSG00000118849	0.01666	0.03923	3	0.492156178
ENTPD5.4437.56.3	ENSG00000187097	-0.01656	0.03909	1	0.49298996
EPHB2.8225.86.3	ENSG00000133216	0.01452	0.03479	1	0.49848604
RNASE6.5646.20.3	ENSG00000169413	-0.01372	0.03338	3	0.504011653
ESAM.2981.9.3	ENSG00000149564	-0.01732	0.04232	1	0.505578617
CCL16.4913.78.1	ENSG00000161573	-0.01427	0.03512	2	0.508114889
S100A6.13090.17.3	ENSG00000197956	0.01757	0.04326	1	0.508477721
TEK.3773.15.4	ENSG00000120156	-0.01367	0.03392	1	0.511142373
IL23R.5088.175.3	ENSG00000162594	-0.01577	0.03932	1	0.512841627
SPARCL1.4467.49.2	ENSG00000152583	0.01668	0.04159	1	0.512963109

EMILIN3.8773.172.3	ENSG00000183798	0.03729	0.0934	3	0.514543661
CCL27.2192.63.10	ENSG00000213927	-0.01846	0.04638	1	0.515761105
F10.4878.3.1	ENSG00000126218	0.01922	0.04841	3	0.516492254
ERAP2.8960.3.3	ENSG00000164308	-0.02308	0.05826	1	0.51734591
IL15RA.3445.53.2	ENSG00000134470	0.02213	0.05626	1	0.519911072
ERAP1.4964.67.1	ENSG00000164307	0.02104	0.05366	6	0.521012357
CD55.5069.9.3	ENSG00000196352	0.02005	0.05137	2	0.522605151
LPO.4801.13.3	ENSG00000167419	0.0191	0.04908	1	0.523709275
IL18R1.14079.14.3	ENSG00000115604	-0.01464	0.03773	3	0.524814562
PRSS22.4534.10.2	ENSG00000005001	-0.0117	0.03018	1	0.524937444
PSG4.5649.83.3	ENSG00000243137	0.01641	0.04261	2	0.527274933
PGM1.9173.21.3	ENSG00000079739	-0.01935	0.05068	1	0.530358517
BPIFB1.11246.3.3	ENSG00000125999	0.01569	0.04124	1	0.531718161
DPP7.8346.9.3	ENSG00000176978	0.01658	0.04398	2	0.534690758
B4GALT6.10832.24.3	ENSG00000118276	-0.01584	0.04202	1	0.534814798
STC1.4930.21.1	ENSG00000159167	-0.01653	0.04395	1	0.535683487
DLK1.6496.60.3	ENSG00000185559	-0.01708	0.04547	1	0.536056002
PTPRU.8337.65.3	ENSG00000060656	0.01409	0.03754	1	0.536304417
ESD.4984.83.1	ENSG00000139684	-0.00617	0.01647	1	0.536801424
ACP1.3858.5.1	ENSG00000143727	0.01229	0.03293	7	0.538293848
CCDC126.6388.21.3	ENSG00000169193	0.01498	0.04073	2	0.543408894
FUT8.8244.16.3	ENSG00000033170	0.01304	0.03601	3	0.548548721
ENPP7.4435.66.2	ENSG00000182156	0.00994	0.0275	2	0.54930298
CRISPLD2.5691.2.3	ENSG00000103196	-0.01514	0.04224	1	0.552073178
ESAM.7841.84.3	ENSG00000149564	-0.01308	0.03655	1	0.552703774
CCL22.3508.78.3	ENSG00000102962	-0.0256	0.07182	3	0.553966088
TNFRSF11A.8256.57.3	ENSG00000141655	-0.01468	0.04146	1	0.556242023
UROS.11248.43.3	ENSG00000188690	0.01792	0.05079	1	0.557381814
EPHB2.5077.28.3	ENSG00000133216	0.01375	0.03908	1	0.558269159
HPGDS.12549.33.3	ENSG00000163106	-0.01784	0.05078	2	0.558903429
CPB1.6356.3.3	ENSG00000153002	-0.01241	0.03599	1	0.56513926
GRN.4992.49.1	ENSG00000030582	0.0108	0.03159	1	0.567950878
OBP2B.5680.54.3	ENSG00000171102	0.01874	0.05552	1	0.572053708
AGER.4125.52.2	ENSG00000204305	-0.01047	0.03134	1	0.575398841
GXYLT1.8229.1.3	ENSG00000151233	0.0189	0.05678	2	0.576559203
SPINK2.13405.61.3	ENSG00000128040	-0.0146	0.04401	2	0.577849963
THBS2.3339.33.1	ENSG00000186340	0.01491	0.04497	2	0.577849963
GLTPD2.7948.129.3	ENSG00000182327	-0.0189	0.05702	2	0.577979125
SIGLEC7.2742.68.2	ENSG00000168995	-0.01179	0.03601	1	0.581861194
TNFRSF1B.3152.57.1	ENSG00000028137	-0.01844	0.05644	1	0.582639291
GSTA1.12446.49.3	ENSG00000243955	-0.02447	0.07628	1	0.588493005
KDR.3651.50.5	ENSG00000128052	0.01367	0.04261	2	0.588493005
KLK11.2831.29.1	ENSG00000167757	-0.01526	0.04789	3	0.590582012
EPHB2.8348.4.3	ENSG00000133216	0.01291	0.04062	1	0.591235657
CLEC12A.11187.11.3	ENSG00000172322	-0.01095	0.03464	5	0.593198974
PMEL.6472.40.3	ENSG00000185664	0.01151	0.03681	1	0.596610545
ICAM1.4342.10.3	ENSG00000090339	-0.0168	0.05383	5	0.59713636
FAP.5029.3.1	ENSG00000078098	0.00948	0.03041	1	0.59753089

SNCA.8458.16.3	ENSG00000145335	-0.01044	0.03372	1	0.599637489
NDNF.6604.59.3	ENSG00000173376	0.01211	0.03919	1	0.600296645
FUT3.4548.4.2	ENSG00000171124	-0.00869	0.02817	2	0.60082426
SMOC1.13118.5.3	ENSG00000198732	0.01177	0.03817	3	0.600956204
FCN1.3613.62.5	ENSG00000085265	-0.00959	0.03144	3	0.604127708
SNCA.8458.111.3	ENSG00000145335	-0.01083	0.03556	1	0.604657198
IL12RB2.3815.14.1	ENSG00000081985	0.01796	0.059	1	0.604789612
GNLY.3195.50.2	ENSG00000115523	0.00895	0.02984	2	0.609434336
MAN1A2.9077.10.3	ENSG00000198162	-0.01349	0.04505	2	0.609833386
CECR1.6077.63.3	ENSG00000093072	0.013	0.04358	1	0.610898242
RGMA.5483.1.3	ENSG00000182175	-0.01325	0.04491	1	0.614366293
SVEP1.11178.21.3	ENSG00000165124	0.01377	0.04682	4	0.615301906
CRP.4337.49.2	ENSG00000132693	0.01434	0.04903	1	0.616907701
PPT1.9244.27.3	ENSG00000131238	-0.0127	0.04364	2	0.618515889
ARFIP1.13488.3.3	ENSG00000164144	-0.00942	0.03264	1	0.620932672
HFE2.3332.57.1	ENSG00000168509	-0.01615	0.05604	1	0.621335996
PTHLH.2962.50.2	ENSG00000087494	-0.01283	0.04507	1	0.624972711
FCGR2B.3310.62.1	ENSG00000072694	-0.01376	0.0489	4	0.628215671
CD300A.5630.48.3	ENSG00000167851	-0.01381	0.04907	1	0.628351006
PDCD1LG2.3004.67.2	ENSG00000197646	-0.00673	0.02402	1	0.629569789
CBLN4.5688.65.3	ENSG00000054803	-0.01002	0.0358	1	0.629976356
CD209.3029.52.2	ENSG00000090659	-0.01509	0.05407	2	0.63078995
COCH.7227.75.3	ENSG00000100473	0.00969	0.03511	1	0.634050487
CHRDL2.6086.15.3	ENSG00000054938	0.01683	0.06159	2	0.636775162
TREM1.9266.1.3	ENSG00000124731	-0.01148	0.04211	3	0.637593911
OAF.6414.8.3	ENSG00000184232	0.01137	0.0417	1	0.637593911
GSTO1.12436.84.3	ENSG00000148834	-0.0124	0.04554	3	0.637866966
PLG.3710.49.2	ENSG00000122194	0.02139	0.07876	2	0.638549906
PCBD1.11313.100.3	ENSG00000166228	0.0096	0.03583	1	0.642382409
HAVCR1.9021.1.3	ENSG00000113249	0.01371	0.05121	1	0.642793846
FAM3D.13102.1.3	ENSG00000198643	-0.01765	0.06653	1	0.645265782
GP1BA.4990.87.1	ENSG00000185245	-0.01443	0.05443	1	0.645403279
C1RL.9348.1.3	ENSG00000139178	-0.00795	0.03002	1	0.645678326
KYNU.4559.64.2	ENSG00000115919	-0.01109	0.04195	1	0.64622863
HBZ.6919.3.3	ENSG00000130656	0.01116	0.04307	1	0.651885589
EPHA2.4834.61.2	ENSG00000142627	0.01525	0.05896	1	0.652300688
SERPINA12.6551.94.3	ENSG00000165953	0.01233	0.04773	1	0.652715947
GGH.9370.69.3	ENSG00000137563	-0.00919	0.03582	4	0.654517269
LRRC15.6557.50.3	ENSG00000172061	0.02042	0.07986	1	0.65548847
COLEC11.4430.44.3	ENSG00000118004	0.01278	0.05005	2	0.655766118
HINT1.5900.11.2	ENSG00000169567	0.01792	0.07134	1	0.660357753
PTN.3045.72.2	ENSG00000105894	-0.00925	0.03685	1	0.660497203
WISP1.13692.154.3	ENSG00000104415	-0.00901	0.03592	3	0.660776156
CAPN2.14684.17.3	ENSG00000162909	0.01158	0.04678	1	0.664269241
GNPTG.10666.7.3	ENSG00000090581	-0.01558	0.06334	1	0.665949999
TMEM190.10442.1.3	ENSG00000160472	0.00333	0.01388	1	0.672840813
SPINT1.2828.82.2	ENSG00000166145	0.01054	0.04411	1	0.67368767
PLCG1.4563.61.2	ENSG00000124181	-0.02822	0.11818	1	0.673828879

ATP1B2.7218.87.3	ENSG00000129244	0.01272	0.05374	2	0.676090783
QDPR.11257.1.3	ENSG00000151552	0.00714	0.03037	1	0.677648654
NQO2.9754.33.3	ENSG00000124588	0.00983	0.04197	5	0.678924985
MATN2.3325.2.2	ENSG00000132561	0.01058	0.04575	2	0.682193755
FCRL4.8973.23.3	ENSG00000163518	0.0072	0.03169	3	0.686758558
IL22RA2.5087.5.3	ENSG00000164485	0.01031	0.04601	1	0.689908413
IMPAD1.9231.23.3	ENSG00000104331	-0.01055	0.04736	3	0.6914869
APMAP.10605.22.3	ENSG00000101474	-0.01092	0.0493	1	0.69292396
DPT.4979.34.2	ENSG00000143196	-0.00866	0.03912	3	0.693067774
ASIP.5676.54.3	ENSG00000101440	0.00721	0.03292	1	0.695804027
IL22RA2.9456.34.3	ENSG00000164485	0.01114	0.05193	1	0.700863399
ADAM23.7049.2.3	ENSG00000114948	-0.00947	0.04436	4	0.702023298
TMEM132C.7173.141.3	ENSG00000181234	0.00959	0.04511	2	0.702894075
LRPAP1.3640.14.3	ENSG00000163956	-0.00863	0.04101	1	0.705510813
GRAMD1C.8842.16.3	ENSG00000178075	-0.0086	0.04137	2	0.708280143
TPST1.7928.183.3	ENSG00000169902	0.00871	0.04226	1	0.710179248
ADAMTS5.3168.8.2	ENSG00000154736	-0.00689	0.03388	1	0.713401144
GPC1.8697.38.3	ENSG00000063660	-0.00736	0.03637	1	0.714722119
FAM20B.7198.197.3	ENSG00000116199	0.00972	0.04865	1	0.717516467
SEMA5A.13132.14.3	ENSG00000112902	-0.00663	0.03329	3	0.718253096
SELL.4831.4.2	ENSG00000188404	-0.00831	0.04177	2	0.718695329
CEL.9796.4.3	ENSG00000170835	-0.01041	0.05292	1	0.721057163
MANEA.8014.359.3	ENSG00000172469	0.00914	0.04663	7	0.72194427
GRAMD1C.8336.267.3	ENSG00000178075	-0.00843	0.04513	1	0.732650824
AHSG.3581.53.3	ENSG00000145192	0.00732	0.03933	3	0.733548214
IL1RN.5353.89.2	ENSG00000136689	-0.00867	0.04752	1	0.7375965
CBLN1.9313.27.3	ENSG00000102924	-0.00625	0.03422	1	0.7375965
SERPINF2.3024.18.2	ENSG00000167711	-0.00617	0.03413	1	0.7398527
SIRPA.5430.66.3	ENSG00000198053	0.01151	0.06371	2	0.740003296
IL17RA.2992.59.2	ENSG00000177663	0.00786	0.04368	3	0.740605911
GDF15.4374.45.2	ENSG00000130513	0.00688	0.03868	3	0.743171138
NCAM1.4498.62.2	ENSG00000149294	-0.00802	0.04527	1	0.74407811
MTHFS.14107.1.3	ENSG00000136371	0.00967	0.05527	1	0.746652424
PATE4.8065.245.3	ENSG00000237353	0.00623	0.03587	1	0.748169895
LTF.2780.35.2	ENSG00000012223	0.00494	0.02869	1	0.750146143
PLXNC1.4564.2.2	ENSG00000136040	0.00948	0.05536	4	0.751059612
TCN2.5584.21.3	ENSG00000185339	-0.00568	0.03332	4	0.751821493
BOC.4328.2.2	ENSG00000144857	-0.00637	0.03764	1	0.753347054
C1QTNF3.7251.64.3	ENSG00000082196	-0.00997	0.05893	1	0.753499742
LILRA5.8766.29.3	ENSG00000187116	-0.01396	0.08303	1	0.754875024
CCL3.3040.59.1	ENSG00000006075	-0.00689	0.04117	1	0.755946044
IL11RA.3814.63.1	ENSG00000137070	0.00526	0.03158	1	0.756711788
OLA1.12659.13.3	ENSG00000138430	-0.00783	0.04738	1	0.758245109
IL12B.IL23A.10365.132.3	ENSG00000113302	0.00659	0.04007	2	0.759319893
CPZ.6493.9.3	ENSG00000109625	0.01144	0.06978	1	0.759934598
TAPBPL.6364.7.3	ENSG00000139192	-0.00309	0.01985	1	0.769979679
LIPN.8097.77.3	ENSG00000204020	0.00419	0.02811	2	0.778093871
CD274.5060.62.3	ENSG00000120217	-0.00548	0.03684	1	0.778250611

NDST1.6927.7.3	ENSG00000070614	0.00574	0.03869	1	0.77887784
XCL1.14078.69.3	ENSG00000143184	0.00577	0.03895	3	0.779348543
RSPO3.13094.75.3	ENSG00000146374	0.00557	0.03791	1	0.7807621
CD177.13116.25.3	ENSG00000204936	0.0151	0.10287	3	0.780919297
IDO1.9759.13.3	ENSG00000131203	0.0067	0.04753	3	0.788179832
ICAM5.8245.27.3	ENSG00000105376	0.00546	0.03886	1	0.788813945
IL27.EBI3.2829.19.2	ENSG00000197272	0.0088	0.063	1	0.789765954
PENK.9076.25.3	ENSG00000181195	0.00729	0.05302	2	0.792628073
CTSF.9212.22.3	ENSG00000174080	-0.00461	0.03403	1	0.795179927
PTGFRN.12727.7.3	ENSG00000134247	0.00582	0.04329	1	0.796458619
MCL1.10358.33.3	ENSG00000143384	0.00607	0.04783	1	0.806108867
CRELD1.7628.40.3	ENSG00000163703	-0.0054	0.04618	5	0.818983482
TPST2.8024.64.3	ENSG00000128294	0.00355	0.03136	1	0.82359491
ROR2.7861.9.3	ENSG00000169071	0.00702	0.06282	2	0.825744845
NID2.3633.70.5	ENSG00000087303	-0.00455	0.04075	2	0.825744845
DSC2.13126.52.3	ENSG00000134755	-0.00574	0.05326	1	0.830894974
CFI.2567.5.6	ENSG00000205403	0.0036	0.03427	1	0.83440289
LMAN2L.8013.9.3	ENSG00000114988	0.00455	0.04534	1	0.84079107
PSG9.9335.28.3	ENSG00000183668	-0.00621	0.06244	2	0.841804617
CD33.3166.92.1	ENSG00000105383	0.00308	0.03099	4	0.842142767
TMEM2.8992.1.3	ENSG00000135048	0.00297	0.03003	1	0.84281952
ANGPTL1.11142.11.3	ENSG00000116194	-0.00504	0.05277	1	0.847233359
CST7.3302.58.1	ENSG00000077984	-0.00426	0.04482	3	0.847744335
ALDH3A1.11480.1.3	ENSG00000108602	0.00297	0.03173	1	0.849791793
LILRA5.7787.25.3	ENSG00000187116	-0.00229	0.02578	1	0.856486142
AGRP.2813.11.2	ENSG00000159723	0.00084	0.00961	1	0.858385679
FLRT2.13122.19.3	ENSG00000185070	0.00295	0.03459	1	0.861157848
CXCL5.2979.8.2	ENSG00000163735	-0.00375	0.04497	1	0.863941142
TFF1.9185.15.3	ENSG00000160182	-0.00271	0.0338	1	0.868312914
FAM177A1.8039.41.3	ENSG00000151327	-0.00362	0.04633	1	0.871125896
ADAMTS13.3175.51.5	ENSG00000160323	0.00446	0.05761	3	0.872183863
CNTN4.3298.52.2	ENSG00000144619	-0.00236	0.03127	2	0.874836319
PPIE.5238.26.3	ENSG00000084072	0.00269	0.03625	2	0.876610703
NAGPA.11208.15.3	ENSG00000103174	0.00156	0.02133	1	0.878211883
DNAJA4.9744.139.3	ENSG00000140403	0.00346	0.05032	1	0.88465796
FCER2.3291.30.2	ENSG00000104921	0.00226	0.03374	2	0.886821844
LHB.8376.25.4	ENSG00000104826	-0.00536	0.08055	1	0.887544879
PAM.5620.13.3	ENSG00000145730	-0.00318	0.0499	2	0.891719624
SIRPB1.6247.9.3	ENSG00000101307	-0.0026	0.04079	6	0.891719624
SERPINE2.3217.74.2	ENSG00000135919	0.00271	0.04267	1	0.891901815
MRC2.3041.55.2	ENSG00000011028	-0.00302	0.04788	1	0.892631155
CREG1.9357.4.3	ENSG00000143162	0.00268	0.0441	1	0.895924786
CA6.3352.80.3	ENSG00000131686	0.00243	0.04052	3	0.897394838
MAPK3.2855.49.2	ENSG00000102882	-0.00322	0.05442	1	0.898499941
CHST11.7779.86.3	ENSG00000171310	0.0023	0.03979	1	0.90034675
ACP5.3232.28.2	ENSG00000102575	-0.00202	0.0353	2	0.901087237
COLEC12.5457.5.2	ENSG00000158270	0.0018	0.03186	1	0.902571283
LCT.9017.58.3	ENSG00000115850	0.00041	0.00732	2	0.902757079

GALNT16.8923.94.3	ENSG00000100626	-0.00217	0.03954	2	0.904805169
ICAM5.5124.62.3	ENSG00000105376	0.00231	0.0428	1	0.9059257
SPOCK2.5491.12.3	ENSG00000107742	-0.00198	0.03668	1	0.9059257
CPXM1.6255.74.3	ENSG00000088882	0.00264	0.04942	1	0.906861334
SPON1.4297.62.3	ENSG00000152268	0.00208	0.03955	1	0.907986356
PLEKHA7.12731.12.3	ENSG00000166689	0.00235	0.0481	1	0.91383962
FUT10.7156.2.3	ENSG00000172728	0.00243	0.05159	4	0.916315353
S100A12.5852.6.3	ENSG00000163221	-0.00269	0.06229	1	0.922466395
ITIH5.8233.2.3	ENSG00000123243	-0.00138	0.03221	2	0.922659977
IGF2R.3676.15.3	ENSG00000197081	0.00179	0.04347	2	0.925574093
APOA5.11318.20.3	ENSG00000110243	0.00349	0.09321	1	0.931265936
MANBA.6382.17.3	ENSG00000109323	0.00121	0.03365	1	0.933446116
ROR1.2590.69.4	ENSG00000185483	0.00092	0.02609	1	0.935039443
HSP90B1.6393.63.3	ENSG00000166598	0.00137	0.03898	3	0.935239078
HS6ST1.5465.32.3	ENSG00000136720	0.00136	0.03967	1	0.936238849
IGDCC4.9793.145.3	ENSG00000103742	-0.00172	0.05018	4	0.936439125
LILRA4.8299.66.3	ENSG00000239961	0.00025	0.00796	1	0.94127917
CTSB.3061.61.2	ENSG00000164733	0.00111	0.03542	2	0.94127917
SPINT3.7926.13.3	ENSG00000101446	0.00133	0.04498	2	0.943929091
ESM1.3805.16.2	ENSG00000164283	0.00121	0.04338	1	0.946806719
TNFSF12.5939.42.3	ENSG00000239697	-0.00102	0.04	2	0.95075461
CA10.13666.222.3	ENSG00000154975	-0.00124	0.04975	1	0.951802197
SEMA4D.5737.61.3	ENSG00000187764	-0.00082	0.03655	5	0.955605732
DKK3.3607.71.6	ENSG00000050165	-0.00097	0.0428	1	0.955605732
SYT11.7089.42.3	ENSG00000132718	0.00131	0.05858	1	0.955818587
QSOX1.6217.23.3	ENSG00000116260	-0.00124	0.05503	4	0.955818587
MICB.5102.55.3	ENSG00000204516	0.00086	0.04272	13	0.959679959
SIGLEC14.5125.6.3	ENSG00000254415	0.00109	0.05487	1	0.960112667
CCL7.4886.3.1	ENSG00000108688	0.00071	0.03626	1	0.960546145
DCBLD2.9338.2.3	ENSG00000057019	0.00087	0.04652	3	0.962287969
ISLR2.13124.20.3	ENSG00000167178	0.00072	0.0416	1	0.964704718
LYZ.4920.10.1	ENSG00000090382	-0.00039	0.03154	2	0.973280093
FKBP7.9288.7.3	ENSG00000079150	0.00044	0.0372	4	0.974439284
OSMR.10892.8.3	ENSG00000145623	0.00037	0.03334	1	0.976076457
GNRH2.10708.3.3	ENSG00000125787	0.00042	0.03861	1	0.976311774
VWC2.11121.56.3	ENSG00000188730	-0.00035	0.04238	2	0.981592599
FSTL4.9350.3.3	ENSG00000053108	-0.0003	0.03655	1	0.981592599
AMY1A.7918.114.3	ENSG00000237763	0.00018	0.03582	3	0.988171823
GP5.7185.29.3	ENSG00000178732	0.00005	0.03907	1	0.996362831
CROT.13929.27.3	ENSG00000005469	0	0.06932	1	1

REVIEWERS' COMMENTS:

Reviewer #2 (Remarks to the Author):

I applaud the authors for their effort to improve this manuscript. These changes have addressed all my questions. I believe the method is novel and will be of interest to the community. I recommend to publish.

REVIEWERS' COMMENTS:

Reviewer #2 (Remarks to the Author):

I applaud the authors for their effort to improve this manuscript. These changes have addressed all my questions. I believe the method is novel and will be of interest to the community. I recommend to publish.

We thank the Reviewer for their kind words, as well as their helpful criticism in previous iterations of the review process.
